# First DMAP-mediated direct conversion of Morita–Baylis–Hillman alcohols into γ-ketoallylphosphonates: Synthesis of γ-aminoallylphosphonates

**DOI:** 10.3762/bjoc.12.290

**Published:** 2016-12-30

**Authors:** Marwa Ayadi, Haitham Elleuch, Emmanuel Vrancken, Farhat Rezgui

**Affiliations:** 1 Université de Tunis El Manar, Faculté des Sciences de Tunis, Laboratoire de Chimie Organique Structurale LR99ES14, Campus Universitaire, 2092 Tunis, Tunisie; 2 Institut Charles Gerhardt UMR 5253 CNRS-UM-ENSCM, 8 rue de l'Ecole Normale 34296 Montpellier Cedex 5, France

**Keywords:** allylic substitution, γ-aminoallylphosphonate, Arbuzov reaction, γ-ketoallylphosphonate, organophosphorus chemistry

## Abstract

An efficient synthesis of a series of γ-ketoallylphosphonates through a direct conversion of both primary and secondary Morita–Baylis–Hillman (MBH) alcohols by trialkyl phosphites with or without DMAP, used as additive, and under solvent-free conditions, is described herein for the first time. Subsequently, a highly regioselective Luche reduction of the primary phosphonate **2a** (R = H) gave the corresponding γ-hydroxyallylphosphonate **5** that further reacted with tosylamines in the presence of diiodine (15 mol %) as a catalyst, affording the corresponding S_N_2-type products **6a–d** in 63 to 70% isolated yields. Alternatively, the alcohol **5** produced the corresponding acetate **7** which, mediated by Ce(III), was successfully converted into the corresponding γ-aminoallylphosphonates **8a–d**.

## Introduction

Phosphonates and their derivatives are an important class of substances that have a wide range of applications in numerous areas such as medicinal [1–3] and agricultural chemistry [4–5]. Among them, multifunctional derivatives have been exploited as valuable building blocks in natural product syntheses, e.g., calyculins A and B as potent serine-threonine protein phosphatase inhibitors [6], as well as ligands in enantioselective reactions [7]. Furthermore, allylphosphonates are important bioactive compounds that exhibit interesting antimicrobial and antimalarial properties [8–9], as well as useful substrates for the synthesis of valuable organic compounds [10–12].

Historically, the Michaelis−Arbuzov rearrangement [13] is the most widely and generally high yielding strategy for phosphonate synthesis. The current three step protocol involves firstly the mesylation of corresponding alcohols and then the conversion of the intermediate mesylates into their halides. Further an Arbuzov reaction of alkyl phosphites with such halides affords the phosphonates.

**Scheme 1:**
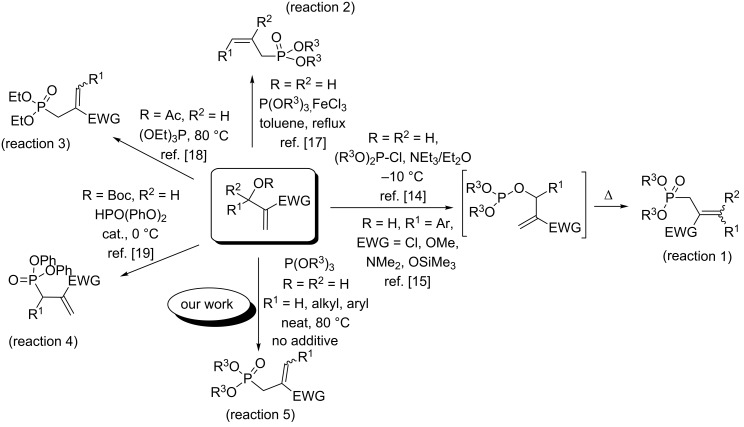
Synthesis of allylphosphonates from acyclic MBH adducts.

Interestingly, a direct conversion of common allyl alcohols into the corresponding phosphonates, with or without catalyst, was previously reported. Accordingly, Bodalski and co-workers [14] and then Swamy’s research group [15] described a direct route to allylphosphonates by treatment of Morita–Baylis–Hillman alcohols with chlorophosphites without any additive, followed by thermal Arbuzov rearrangement of the intermediate allyl phosphites (Scheme 1, reaction 1).

Recently, an efficient protocol for the conversion of common allyl and benzyl alcohols into the corresponding phosphonates through their treatment with triethyl phosphite and ZnI_2_, was described [16]. Similarly, Das and co-workers [17] have directly converted acyclic Morita–Baylis–Hillman (MBH) alcohols into the corresponding allylphosphonates upon their treatment with trialkyl phosphite in the presence of FeCl_3_ (Scheme 1, reaction 2).

On the other hand, treatment of MBH acetates with triethyl phosphite, without any additive, provided, after thermal Arbuzov rearrangement, a variety of diethyl allylphosphonates (Scheme 1, reaction 3) [18]. Moreover, the asymmetric allylic substitution of MBH carbonates with diphenyl phosphonate using chiral thiourea phosphite as catalyst, afforded the related allylphosphonates (Scheme 1, reaction 4) [19].

**Scheme 2:**
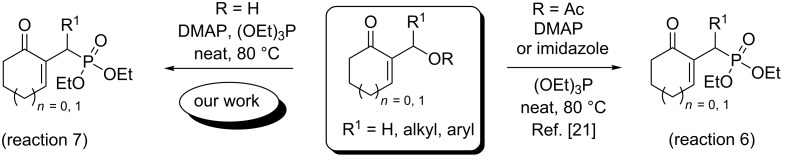
Synthesis of γ-ketoallylphosphonates from cyclic MBH adducts.

We have previously reported a direct nucleophilic allylic substitution of cyclic MBH alcohols by β-dicarbonyl compounds [20]. In addition, in our recent work [21], we have described an efficient protocol for the synthesis of a new series of allylphosphonates in high yields and selectivity, using cyclic MBH acetates as starting materials, in the presence of DMAP or imidazole as additives (Scheme 2, reaction 6).

Moreover, it has been demonstrated that the presence of a nitrogen atom and a phosphonate moiety in multifunctional compounds may improve their synthetic and biological potentials. Aminophosphonates and their derivatives [22–26] are thus recognized as promising compounds and a new class of drugs that are widely used in a variety of commercial applications. Indeed, they are known to influence various biochemical processes in plants, modifying or inhibiting them, or to have biological activities as for example antibiotics, antibacterial, anti-cancer or antithrombotic agents [22–26].

In this context, α-aminophosphonates have particularly attracted considerable attention owing to their biological activities [27–33] since they are considered as important surrogates for α-amino carboxylic acids, peptide mimics as well as versatile intermediates for the design of potential anticancer agents.

β-Aminophosphonates present also an important place among the various compounds containing both a P–C bond and an amino group. Indeed, they are known for their important role in medicinal chemistry [34–36] as anti-HIV agents, enzyme inhibitors and antibacterial agents. In addition, α- and β-aminophosphonates have been widely described in literature. However, γ-aminophosphonates have received much less attention. These derivatives were originally isolated from microorganisms [37–38], e.g., the fosmidomycin [39] is as an antimalarial drug that was isolated from broths of bacteria of the genus Streptomyces [40]. The γ-aminophosphonates were prepared through numerous synthetic methods [41–45]. For instance, they were prepared by reductive amination of γ-aminophosphonyl ketones using sodium borohydride [41], or by conjugate addition of diethyl methylphosphonite to 2-cyclohexenone followed by Bucherer–Bergs amino acid synthesis [42]. Another synthetic approach for a series of α-fluorinated-γ-aminophosphonates has been reported through a palladium-catalyzed hydrogenation of α-fluorovinylphosphonates [43].

In continuation of our interest [21] in the construction of carbon–phosphorus bonds using both cyclic and acyclic MBH adducts, we report herein, for the first time, a direct and facile access to γ-ketoallylphosphonates from primary and secondary MBH alcohols, with or without DMAP used as additive, and under solvent-free conditions (Scheme 1, reaction 5 and Scheme 2, reaction 7). Taking into consideration the importance of aminophosphonates (vide infra), we developed in the second part of this study an efficient conversion of these γ-ketoallylphosphonates into related γ-aminoallylphosphonates.

## Results and Discussion

In our first attempt, a mixture of cyclic MBH alcohol **1a** and triethyl phosphite was reacted in toluene without any additive. After stirring the reaction mixture at 110 °C for 72 h, the starting materials were completely recovered (Table 1, entry 1).

**Table 1:** Optimization of the reaction conditions for the γ-ketophosphonate **2a** from **1a**^a^.

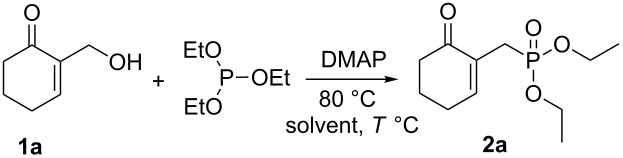					

Entry	DMAP (equiv)	Solvent	*T* (°C)	*t* (h)	Yield **2a** (%)^b^

1	0	toluene	110	72	none^c^
2	0.5	toluene	110	72	30
3	1	toluene	110	120	45
4	1	THF	66	120	trace
5	0	none	rt/80	8	none^c^
6	0.5	none	80	4	35
**7**	**1**	**none**	**80**	**1**	**75**

^a^Reaction conditions: MBH adduct **1a** (1 mmol), triethyl phosphite (2 mmol), DMAP (0–1 equiv) and solvent (5 mL) at the indicated temperature. ^b^Yields of isolated pure compounds after column chromatography. ^c^No reaction detected based on TLC and ^1^H NMR analysis.

Under the previous conditions and in the presence of 0.5 equiv of DMAP, a sluggish reaction occurred, affording the desired phosphonate **2a** within 72 h in a 30% yield (Table 1, entry 2). Using 1 equiv of DMAP in refluxing toluene required a longer reaction time of 120 h to completely convert the allyl alcohol **1a** into the phosphonate **2a** in 45% yield (Table 1, entry 3). The nucleophilic substitution of alcohol **1a** with triethyl phosphite in THF at reflux was significantly less effective (Table 1, entry 4). The TLC and ^1^H NMR analyses of the crude reaction mixture conducted without any additive and under solvent-free conditions, at room temperature or at 80 °C, indicated that no reaction occurred and the starting materials were completely recovered (Table 1, entry 5).

However, when 0.5 equiv of DMAP was employed under the previous conditions, the phosphonate **2a** was isolated within 4 h in 35% yield (Table 1, entry 6). After screening several amounts of DMAP, under solvent-free conditions at 80 °C, we observed that the best yield (75%) was obtained using 1 equiv of DMAP (Table 1, entry 7). Under these reaction conditions, the allylic phosphonate **2a** was produced as the sole product within 1 h and its structure was confirmed by ^1^H, ^13^C and ^31^P NMR analysis [21].

A series of γ-ketoallylphosphonates **2a–f** was prepared from different cyclic MBH alcohols and triethyl phosphite using the optimized reaction conditions (Table 2).

**Table 2:** Direct conversion of cyclic MBH alcohols **1a–f** into γ-ketoallylphosphonates **2a–f**^a^.

Entry	MBH alcohol	γ-Ketoallyl- phosphonate	*t* (h)	Yield (%)^b^

**1**	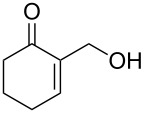 **1a**	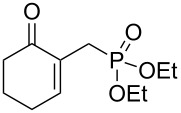 **2a** [21]	1	75
**2**	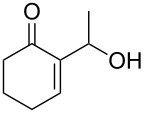 **1b**	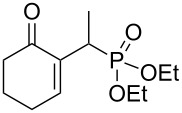 **2b** [21]	1.5	77
**3**	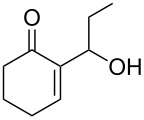 **1c**	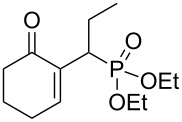 **2c** [21]	1	80
**4**	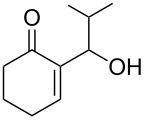 **1d**	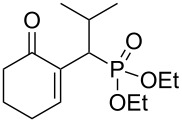 **2d** [21]	2	84
**5**	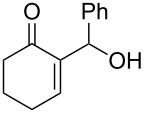 **1e**	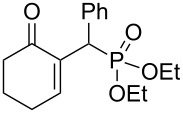 **2e** [21]	4	64
**6**	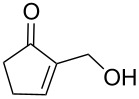 **1f**	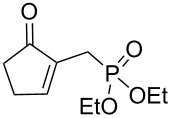 **2f** [21]	4	58

^a^Reaction conditions: MBH alcohols (1 mmol), trialkyl/triaryl phosphite (2 mmol) and DMAP (1 equiv) at 80 °C. ^b^Yields of isolated pure compounds after column chromatography.

These adducts were obtained from primary (**1a** and **1f**) or secondary (**1b–e**) MBH alcohols (R = linear/branched alkyl or aryl). The total conversion of alcohols **1a–f** into phosphonates **2a–f** was complete within 1–4 h in 58–84% yields. Our results suggested that this reaction worked well with six-membered cyclic MBH alcohols **1a–e** (Table 2, entries 1–5), as well as five-membered cyclic MBH alcohol **1f** (Table 2, entry 6).

**Scheme 3:**
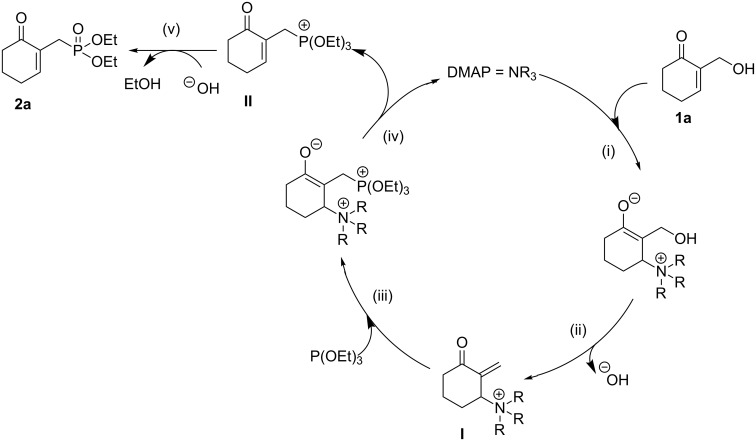
Proposed mechanism for DMAP-mediated direct nucleophilic α-substitution of MBH alcohol **1a**.

A putative reaction pathway could start from a first β-conjugate addition of DMAP onto the allylic alcohol **1a** (i), followed by the elimination of the hydroxide ion that would afford the intermediate **I** (ii). Similarly, a second β’-conjugate addition of triethyl phosphite onto **I** (iii), followed by the release of DMAP would provide the phosphonium intermediate **II** (iv). Finally, the hydroxide ion is expected to react with **II** via an Arbuzov rearrangement to provide the desired γ-ketoallylphosphonate **2a** (v) (Scheme 3).

**Scheme 4:**
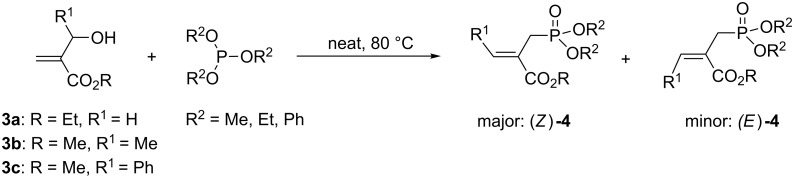
Direct conversion of acyclic MBH alcohols **3a–c** into γ-ketoallylphosphonates **4a–f**.

We next investigated the scope and the limitations of this synthetic method. We have thus examined, the behavior of the acyclic MBH alcohol **3a** [46–47] towards triethyl phosphite under the above conditions (1 equiv of DMAP, solvent-free conditions, 80 °C, Scheme 4).

We were pleased to note that the nucleophilic substitution reaction worked also well and gave the allylphosphonate **4a** in 90% yield within 1 h (Table 3, entry 1). More interestingly, repeating this reaction without DMAP and under solvent-free conditions at 80 °C gave, in only 30 min, the phosphonate **4a** in 92% yield (Table 3, entry 2). Encouraged by these results, the behavior of a variety of acyclic MBH alcohols **3a–c** towards trialkyl/triaryl phosphites was examined to determine the substrate scope of the present procedure (Table 3).

**Table 3:** Direct conversion of acyclic MBH alcohols **3a–c** into γ-ketoallylphosphonates **4a–f^a^**.

Entry	MBH alcohol	Phosphite reagent/DMAP (equiv)	γ- Ketoallylphosphonate	*t* (min)	Yield (%)^b^	Ratio *Z*/*E*^c^

**1**	**3a**	P(OEt)_3_/1	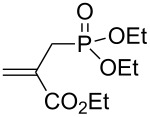 **4a** [18–48]	60	90	N/A^d^
**2**	**3a**	P(OEt)_3_/0	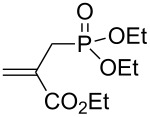 **4a** [18–48]	30	92	N/A^d^
**3**	**3a**	P(OMe)_3_/0	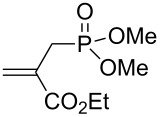 **4b** [18–48]	30	60	N/A^d^
**4**	**3a**	P(OPh)_3_/0	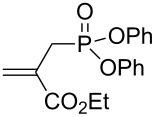 **4c**	30	62	N/A^d^
**5**	**3b**	P(OEt)_3_/0	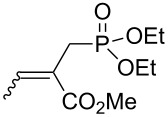 **4d** [18–48]	30	92	87:13
**6**	**3b**	P(OMe)_3_/0	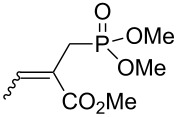 **4e** [18–48]	30	56	94:06
**7**	**3c**	P(OEt)_3_/0	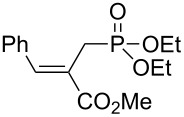 **4f** [18–48]	30	82	100:0

^a^Reaction conditions: MBH alcohols (1 mmol), trialkyl/triaryl phosphite (2 mmol) at 80 °C. ^b^Yields of isolated pure compounds after column chromatography. ^c^Estimated from the integrated intensities of the ^31^P NMR signal of the crude products. ^d^Means not applicable.

As shown in entries 3 and 4 (Table 3), the substitution of the primary alcohol **3a** with a trialkyl/triaryl phosphites gave the corresponding phosphonates **4b** and **4c** in 60–62% yields.

The reaction of secondary alcohols **3b** and **3c** with trialkyl phosphites was also surveyed. Under the previous reaction conditions (solvent-free, 80 °C, without any additive) an S_N_2’-type reaction followed by an Arbuzov rearrangement gave the corresponding primary allylphosphonates **4d–f** with a high *Z*-stereoselectivity (*Z*/*E* = 87–100/13–0) and in 56–92% yields (Table 2, entries 5–7). It seems that the *Z*/*E* ratios depend on the nature of the R allylic group. Indeed, when it is an alkyl group, i.e., R^1^ = Me, the phosphonates **4d** and **4e** were obtained in high *Z*-diastereoselectivities (de = 74–88%), whereas the phosphonate **4f** was provided with an excellent *Z*-diastereoselectivity (de = 100%) when R^1^ = Ph. In this work, the observed high *Z*-diastereoselectities are in good agreement with a previous report by Basavaiah and co-workers on the nucleophilic addition of triethyl phosphite onto Morita–Baylis–Hillman acetates [18–48].

We envisaged in the second part of this study a further functionalization of these allylphosphonates as the literature survey revealed that the aminophosphonates are useful molecules in organic synthesis and in biology (vide supra).

**Scheme 5:**
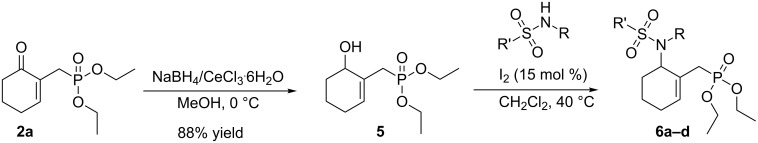
I_2_-Catalyzed direct synthesis of γ-tosylaminophosphonates **6** from alcohol **5**.

The synthesis of γ-tosylaminophosphonates was achieved in a two-step sequence. First, the γ-hydroxyphosphonate **5** was prepared and isolated in a good yield (88%) via a highly selective Luche reduction [49–50] of the γ-ketophosphonate **2a** using NaBH_4_ in the presence of CeCl_3_·6H_2_O in methanol at 0 °C. The TLC of the reaction mixture and analysis of ^1^H NMR and ^31^P NMR spectra showed the exclusive formation of the 1,2-adduct **5** (Scheme 5). The literature survey revealed that γ-hydroxyphosphonates may display interesting biological activities [51–52].

The next step was to realize the nucleophilic substitution of the γ-hydroxyphosphonate **5a** with a variety of differently substituted tosylamines catalyzed by 15 mol % of iodine [53] in refluxing methylene chloride. Under these optimized conditions, the γ-tosylaminophosphonates **6a–d** were obtained in good yields (63–70%) (Table 4, entries 1–4).

**Table 4:** Synthesis of γ-tosylaminophosphonates **6a–d** from alcohol **5**.

Entry	Tosylamine	γ-Tosylaminophosphonate	*t* (h)	Yield (%)^a^

**1**	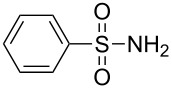	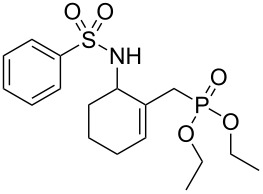 **6a**	25	69
**2**	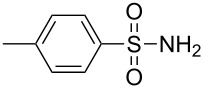	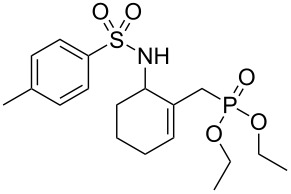 **6b**	12	63
**3**	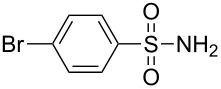	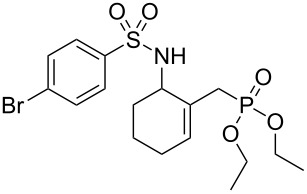 **6c**	20	68
**4**	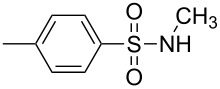	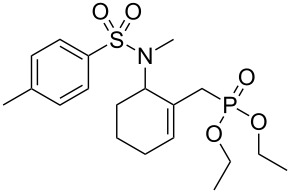 **6d**	4	70

^a^Yields of isolated pure compounds after column chromatography.

**Scheme 6:**
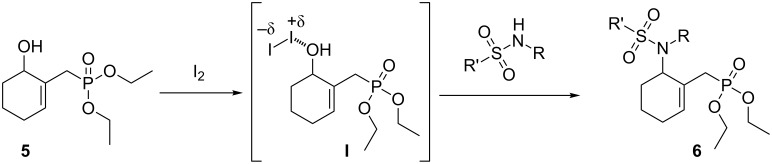
Proposed mechanism for I_2_-catalyzed direct nucleophilic substitution of γ-hydroxyallylphosphonate **5** with tosylamines.

Scheme 6 illustrates a possible mechanism for the I_2_-catalyzed substitution reaction of γ-hydroxyallylphosphonate **5** with tosylamines. This could first involve an activation of the hydroxy moiety of adduct **5** through a coordination of the iodine catalyst with the hydroxy group to give an iodine-coordinated intermediate **I** that would subsequently undergo a nucleophilic attack by the tosylamine. The release of diiodine and elimination of a molecule of water is then expected to afford the desired γ-tosylaminoallylphosphonates **6**.

**Scheme 7:**
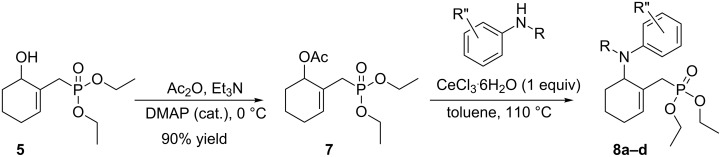
Ce(III)-mediated conversion of acetate **7** into γ-aminophosphonates **8a–d**.

However, this reaction failed using primary, secondary amines or aniline as nucleophiles. Therefore, an alternative synthesis of γ-aminophosphonates **8** has been developed. Following our previous report [54], we have first converted the MBH alcohol **5** into its acetate **7** in 90% yields using a mixture of Ac_2_O and DMAP as reagents (Scheme 7).

The MBH acetate **7** was further converted into the γ-aminophosphonates **8a–c** within short reactions times of 1–2 h and in 60–81% isolated yields using anilines, and 1 equiv of cerium(III) chloride hexahydrate in refluxing toluene (Table 5, entries 1–3). The nucleophilic allylic substitution of the acetate **7** worked also with β-naphthylamine and gave the corresponding γ-aminophosphonate **8d** in only a modest yield (Table 5, entry 4).

**Table 5:** Synthesis of γ-aminophosphonates **8a–d** from acetate **7**.

Entry	Aromatic amine	γ-Aminophosphonate	*t* (h)	Yield (%)^a^

**1**	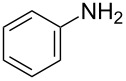	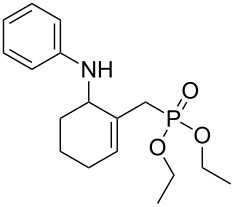 **8a**	2	60
**2**	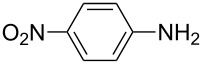	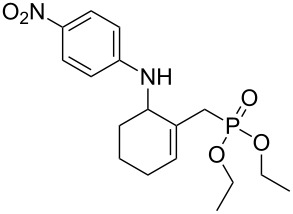 **8b**	1	81
**3**	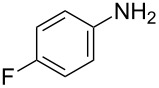	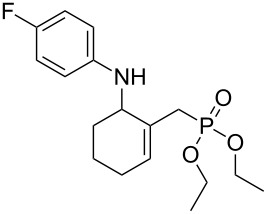 **8c**	2	66
**4**	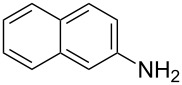	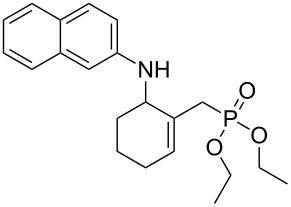 **8d**	4	40

^a^Yields of isolated pure compounds after column chromatography.

## Conclusion

We have developed a mild, direct and convenient procedure for the conversion of both cyclic and acyclic MBH alcohols, with trialkyl and triaryl phosphites, into γ-ketoallylphosphonates under solvent-free conditions, in the presence or absence of the promoter DMAP. The corresponding products have been further involved in two alternative efficient synthetic routes for γ-amino- and γ-tosylaminophosphonates that were readily obtained in high yields and selectivities.

## Supporting Information

File 1 Experimental procedures and characterization for synthesized compounds.

File 2 Spectral data for synthesized compounds.
