# Supplementary material for: First DMAP-mediated direct conversion of Morita–Baylis–Hillman alcohols into γ-ketoallylphosphonates: Synthesis of γ-aminoallylphosphonates
Source: Beilstein J Org Chem. 2016 Dec 30;12:2906–15. doi: 10.3762/bjoc.12.290 (PMC5238584; doi:10.3762/bjoc.12.290)
Supplement: File 1 — Experimental procedures and characterization for synthesized compounds. [file Beilstein_J_Org_Chem-12-2906-s001.pdf]

Supporting Information File 1

for

First DMAP-mediated direct conversion of Morita–Baylis–Hillman alcohols into γ-ketoallylphosphonates: Synthesis of γ-aminoallylphosphonates

Marwa Ayadi[1,2], Haitham Elleuch[1], Emmanuel Vrancken[2], and Farhat Rezgui*[1]

Address: [1]Université de Tunis El Manar, Faculté des Sciences de Tunis, Laboratoire de Chimie Organique Structurale LR99ES14, Campus Universitaire, 2092 Tunis, Tunisie and [2]Institut Charles Gerhardt UMR 5253 CNRS-UM-ENSCM, 8 rue de l'Ecole Normale 34296 Montpellier Cedex 5, France.

Email: Farhat Rezgui* - rez_far@yahoo.fr

*Corresponding author

Experimental procedures and characterization for synthesized compounds

Table of contents:

Analytical data ……………………………………………………………………………………………..Pages S2–S6

Analytical data

Materials and methods

^{1}H NMR and ^{13}C NMR spectra were recorded on a 300 MHz for ^{1}H and 75 MHz for ^{13}C in $CDCl_3$, using TMS as an internal standard (chemical shifts in δ values, *J* in Hz). ^{31}P NMR spectra were recorded on a 121 MHz using 85% H_3PO_4 as an internal standard. High resolution mass spectra (HRMS) were recorded as TOF-HRMS on a micro mass spectrometer. Analytical thin layer chromatography (TLC) was performed using silica gel 60 F254 precoated silica gel plates. Visualization was achieved by UV light (254 nm). Flash chromatography was performed using silica gel 60.

General method for the addition of trialkyl phosphite to acyclic MBH alcohols 3

A mixture of alcohol **3** (1 mmol) and trialkyl phosphite (2 mmol) was heated at 80 °C. After the reaction was finished, the reaction mixture was cooled and the crude product was purified by flash chromatography on silica gel (diethyl ether/methylene chloride = 8:2) to give the corresponding phosphonates **4a–f**.

Ethyl 2-(diphenoxyphosphoryl)methyl acrylate (4c)

Yellow oil (215 mg, 62% yield). IR (FT-IR) 1735, 1598, 1497, 1295, 1204, 939, 766 cm^{-1}; ^{1}H NMR (300 MHz, $CDCl_3$) δ 7.29 – 7.09 (m, 10H), 6.43 (d, *J* = 6.0 Hz, 1H), 5.96 (d, *J* = 6.0 Hz, 1H), 4.24 – 4.17 (m, 2H), 3.29 (d, $J_{H\text{-}P}$ = 21.0 Hz, 2H), 1.29 – 1.23 (m, 3H); ^{13}C NMR (75 MHz, $CDCl_3$) δ 165.8 (d, $^{3}J_{C\text{-}P}$ = 6.7 Hz), 150.7 (d, $^{2}J_{C\text{-}P}$ = 9.0 Hz), 131.1 (d, $^{2}J_{C\text{-}P}$ = 10.5 Hz), 129.3, 129.2 (d, $^{3}J_{C\text{-}P}$ = 9.7 Hz), 125.2 (d, $^{4}J_{C\text{-}P}$ = 0.7 Hz, 2C), 120.5 (d, $^{3}J_{C\text{-}P}$ = 4.5 Hz, 2C), 61.4, 29.2 (d, $^{1}J_{C\text{-}P}$ = 141.7 Hz), 14.1; ^{31}P NMR (121 MHz, $CDCl_3$) δ 18.0; HRMS (ESI+) m/z calcd for $[C_{18}H_{19}PO_5 + H]^+$ 347.1048, found 347.1049.

Reduction of γ-ketophosphonate 2a

A mixture of γ-ketophosphonate **2a** (5 mmol) and $CeCl_3 \cdot 6H_2O$ (5 mmol) in methanol was stirred at 0 °C. $NaBH_4$ (5 mmol) was thereafter added and the mixture was stirred at 0 °C for

15 minutes then half an hour at room temperature. After completion, the reaction was neutralized with 2 M aqueous HCl solution and extracted with CH_2Cl_2.the combined organic layers were dried over $MgSO_4$ and concentrated under vacuum. The crude product was chromatographed on a silica gel column using ether/CH_2Cl_2 as eluent.

Diethyl (6-hydroxycyclohex-1-en-1-yl)methylphosphonate (5)

Pale yellow oil (1092mg, 88% yield). IR (FT-IR) 3413, 2951, 1235, 1056, 1040, 962 cm^{-1}; ^{1}H NMR (300 MHz, $CDCl_3$) δ 5.71-5.70 (m, 1H), 4.16-4.07 (m, 5H), 2.78 (dd, *J* = 15.0, 21.0 Hz, 1H), 2.54 (dd, *J* = 15.0, 21.0 Hz, 1H), 2.04 (m, 2H), 1.77-1.73 (m, 3H), 1.56 (s, 1H), 1.35-1.26 (m, 7H); ^{13}C NMR (75 MHz, $CDCl_3$) δ 130.4 (d, $^3J_{C-P}$ = 10.5 Hz), 130.2 (d, $^2J_{C-P}$ = 10.5 Hz), 67.3, 62.5 (d, $^2J_{C-P}$ = 6.7 Hz), 62.0 (d, $^2J_{C-P}$ = 6.7 Hz), 32.5 (d, $^1J_{C-P}$ = 282.7 Hz), 31.5, 25.7 (d, $^4J_{C-P}$ = 2.2Hz), 18.1, 16.4 (d, $^3J_{C-P}$ = 3.0 Hz), 16.3 (d, $^3J_{C-P}$ = 3.0 Hz); ^{31}P NMR (121MHz, $CDCl_3$) δ 29.8; HRMS (ESI+) m/z calcd for $[C_{11}H_{21}PO_4+Na]^+$ 271.1075, found 271.1075.

General method for the preparation of γ-tosylaminophosphonates 6

MBH alcohol **5** (1 mmol) was taken in CH_2Cl_2 (5 mL) and I_2 (15 mol %) was added. The mixture was kept at room temperature. Sulfonamide (1.2 mmol) and 0.15 g of $MgSO_4$ were subsequently added and the mixture was stirred at 40°C. The reaction was monitored by TLC. After completion, the reaction was quenched with water (10 mL) and the mixture was extracted with methylene chloride. The extract was dried and concentrated and the residue was subjected to column chromatography (ether/petroleum ether) to obtain the pure γ-tosylaminophosphonates **6a–d**.

Diethyl ((6-(phenylsulfonamido)cyclohex-1-en-1-yl)methyl)phosphonate (6a)

Pale yellow crystal (267mg, 69% yield, mp 93°C). IR (FT-IR) 3126, 2954, 2887 1452, 1326, 1237, 1148, 1013, 962 cm^{-1}; ^{1}H NMR (300 MHz, $CDCl_3$) δ 7.97 - 7.94 (m, 2H), 7.57 - 7.47 (m, 3H), 6.54 (d, *J* = 9.0 Hz, 1H), 5.81 - 5.80 (m, 1H), 4.11 - 3.99 (m, 4H), 3.70 - 3.66 (m, 1H), 2.50 - 2.37 (m, 1H), 1.99 - 1.79 (m, 4H), 1.63 - 1.57 (m, 3H), 1.33 - 1.27 (m, 6H); ^{13}C NMR (75 MHz, $CDCl_3$) δ 140.5, 131.9 (d, $^3J_{C-P}$ = 9.7 Hz), 131.7, 128.3, 126.5, 125.4 (d, $^2J_{C-P}$ = 10.5 Hz), 61.9 (d, $^2J_{C-P}$ = 7.5 Hz), 61.4 (d, $^2J_{C-P}$ = 6.7 Hz), 51.7 (d, $^3J_{C-P}$ = 3.0 Hz), 30.0, 30.2 (d, $^1J_{C-P}$ = 136.5 Hz), 24.7 (d, $^4J_{C-P}$ = 3.0 Hz), 17.1, 15.8 (d, $^3J_{C-P}$ = 5.2 Hz), 15.8 (d, $^3J_{C-P}$ = 6.7 Hz); ^{31}P NMR (121 MHz, $CDCl_3$) δ 28.8; HRMS (ESI+) m/z calcd for $[C_{17}H_{26}NO_5PS+H]^+$ 388.1348, found 388.1346.

Diethyl ((6-(4-methylphenylsulfonamido)cyclohex-1-en-1-yl)methyl)phosphonate (6b)

Pale yellow crystal (253mg, 63% yield, mp 96°C). IR (FT-IR) 3126, 2947, 1333, 1237, 1148, 1029 cm^{-1}; ^{1}H NMR (300 MHz, $CDCl_3$) δ 7.83 (d, J = 6.0 Hz, 2H), 7.29 (d, J = 6.0 Hz, 2H), 6.35 (d, J = 6.0 Hz, 1H), 5.85 - 5.77 (m, 1H), 4.09 - 4.02 (m, 4H), 3.69 - 3.68 (m, 1H), 2.51 - 2.38 (m, 5H), 2.00 - 1.79 (m, 4H), 1.63 - 1.58 (m, 2H), 1.34 - 1.27 (m, 6H); ^{13}C NMR (75 MHz, $CDCl_3$) δ 142.8, 138.1, 132.4 (d, $^{3}J_{C-P}$ = 10.5 Hz), 129.46, 127.18, 126.0 (d, $^{2}J_{C-P}$ = 10.5 Hz), 62.4 (d, $^{2}J_{C-P}$ = 6.7 Hz), 61.9(d, $^{2}J_{C-P}$ = 6.7 Hz), 52.2 (d, $^{3}J_{C-P}$ = 3.0 Hz), 30.8 (d, $^{1}J_{C-p}$ = 136.5 Hz), 30.58, 25.2 (d, $^{4}J_{C-P}$ = 3.0 Hz), 21.4, 17.71, 16.4 (d, $^{3}J_{C-P}$ = 6.0 Hz), 16.3 (d, $^{3}J_{C-P}$ =6.0 Hz); ^{31}P NMR (121 MHz, $CDCl_3$) δ 28.5; HRMS (ESI+) m/z calcd for $[C_{18}H_{28}NO_5PS+H]^+$ 402.1504, found 402.1501.

Diethyl ((6-(4-bromophenylsulfonamido)cyclohex-1-en-1-yl)methyl)phosphonate (6c)

Pale yellow crystal (317mg, 68% yield, mp 92°C). IR (FT-IR) 3110, 2954, 1333, 1229, 1148, 1036, 962 cm^{-1}; ^{1}H RMN (300 MHz, $CDCl_3$) δ 7.82 (d, J = 9.0 Hz, 2H), 7.63 (d, J = 9.0 Hz, 2H), 6.66 (d, J = 9.0 Hz, 1H), 5.84 - 5.79 (m, 1H), 4.12 - 4.01 (m, 4H), 3.70 – 3.64 (m, 1H), 2.46 (dd, J = 15.0, 24.0 Hz, 1H), 2.00 - 1.79 (m, 4H), 1.65 - 1.55 (m, 3H), 1.35 - 1.27 (m, 6H); ^{13}C NMR (75 MHz, $CDCl_3$) δ 140.4, 132.8 (d, $^{3}J_{C-P}$ = 9.75 Hz), 132.1, 128.8, 127.0, 125.8 (d, $^{2}J_{C-P}$ = 10.5 Hz), 62.6 (d, $^{2}J_{C-P}$ = 6.7 Hz), 62.1 (d, $^{2}J_{C-P}$ = 6.7 Hz), 52.6 (d, $^{3}J_{C-P}$ = 3.0 Hz), 32.1 (d, $^{1}J_{C-P}$ = 136.5 Hz), 30.8, 25.3 (d, $^{4}J_{C-P}$ = 3.7 Hz), 17.8, 16.5 (d, $^{3}J_{C-P}$ = 6.7 Hz), 16.4 (d, $^{3}J_{C-P}$ = 6.0 Hz); ^{31}P NMR (121 MHz, $CDCl_3$) δ 28.9; HRMS (ESI+) m/z calcd for $[C_{17}H_{25}BrNO_5PS+H]^+$ 466.0453, found 466.0455.

Diethyl ((6-(*N*,4-dimethylphenylsulfonamido)cyclohex-1-en-1-yl)methyl)phosphonate (6d)

Brown oil (291mg, 70% yield). IR (FT-IR) 2954, 1341, 1252, 1162, 1043, 962, 664, 553 cm^{-1}; ^{1}H NMR (300 MHz, $CDCl_3$) δ 7.74 (d, J = 9.0 Hz, 2H), 7.30 (d, J = 9.0 Hz, 2H), 6.12 – 6.11 (m, 1H), 4.59 – 4.50 (m, 1H), 4.12 - 4.07 (m, 4H) , 2.64 (s, 3H), 2.42 (s, 3H), 2.37 (d, J = 21.0 Hz, 2H, H-7), 2.00 – 1.98 (m, 2H), 1.68 – 1.50 (m, 4H), 1.35 – 1.31 (m, 6H); ^{13}C NMR (75 MHz, $CDCl_3$) δ 143.1, 137.0, 133.3 (d, J_{C-P} = 9.7 Hz), 129.6, 127.2, 126.3 (d, $^{2}J_{C-P}$ = 9.7 Hz), 61.9 (d, $^{2}J_{C-P}$ = 2.2 Hz), 61.8 (d, $^{2}J_{C-P}$ = 2.2 Hz), 55.8 (d, $^{3}J_{C-P}$ = 5.2 Hz), 28.8 (d, $^{1}J_{C-P}$ = 138.7 Hz), 29.6, 26.7, 25.0 (d, $^{4}J_{C-P}$ = 2.2 Hz), 21.5, 20.2, 16.4 (d, $^{3}J_{C-P}$ = 6.0 Hz,); ^{31}P NMR (121 MHz, $CDCl_3$) δ 27.8; HRMS (ESI+) m/z calcd for $[C_{19}H_{30}NO_5PS+H]^+$ 416.1661, found 416.1661.

Acetylation of γ-hydroxyphosphonate 5

2-((Diethoxyphosphoryl)methyl)cyclohex-2-en-1-yl acetate (7) [1]

Colorless oil (1305 mg, 90% yield). IR (FT-IR) 2954, 1750, 1252, 1036, 962 cm^{-1}; ^{1}H NMR (300 MHz, $CDCl_3$) δ 5.93 (t, *J* = 3 Hz, 1H), 5.39 – 5.27 (m, 1H), 4.07 - 4.02 (m, 4H), 2.62 – 2.40 (m, 2H), 2.02 (s, 3H), 2.09 - 1.96 (m, 2H), 1.77 - 1.75 (m, 2H), 1.59-1.58 (m, 2H), 1.29 - 1.24 (m, 6H); ^{13}C NMR (75 MHz, $CDCl_3$) δ 170.6, 132.7 (d, $^{2}J_{C-P}$ = 11.2 Hz), 126.5 (d, $^{3}J_{C-P}$ = 10.5 Hz), 69.3 (d, $^{3}J_{C-P}$ = 3.0 Hz), 61.8 (d, $^{2}J_{C-P}$ = 6.7 Hz), 61.6 (d, $^{2}J_{C-P}$ = 6.7 Hz), 30.9 (d, $^{1}J_{C-P}$ = 138.0 Hz), 28.5, 25.2 (d, $^{4}J_{C-P}$ = 3.0 Hz), 21.2, 17.6, 16.3 (d, $^{3}J_{C-P}$ = 6.0 Hz); ^{31}P NMR (121 MHz, $CDCl_3$) δ 27.4; HRMS (ESI+) m/z calcd for $[C_{13}H_{23}PO_5+Na]^+$ 313.1181, found 313.1179.

General method for the preparation of γ-aminophosphonates 8

MBH acetate **7** (1 mmol) and $CeCl_3.6H_2O$ (1 mmol) was taken in 5 mL of toluene. The mixture was kept at room temperature. Aromatic amine (1.2 mmol) was added and the mixture was stirred at 110°C. The reaction was monitored by TLC. After completion, the reaction was quenched with water and the mixture was extracted with methylene chloride. The extract was dried, concentrated and the residue was subjected to column chromatography on silica gel (ether / petroleum ether) to obtain pure γ-aminophosphonates **8a-e**.

Diethyl (6-(phenylamino) cyclohex-1-en-1-yl)methylphosphonate (8a)

Yellow oil (194 mg, 60% yield). IR (FT-IR) 3349, 2947, 1616, 1505, 1244, 1036, 969 cm^{-1}; ^{1}H NMR (300 MHz, $CDCl_3$) δ 7.18 - 7.13 (m, 2H) , 6.68 - 6.63 (m, 3H), 5.89 - 5.85 (m, 1H), 4.33 (s, 1H), 4.12 - 4.01 (m, 5H), 2.74 (dd, *J*= 15.0, 21.0 Hz, 1H), 2.55 (dd, *J*= 15.0, 21.0 Hz, 1H), 2.16 - 2.01 (m, 2H), 1.88 - 1.57 (m, 4H), 1.33 - 1.23 (m, 6H); ^{13}C NMR (75 MHz, $CDCl_3$) δ 147.7, 130.5 (d, $^{3}J_{C-P}$ = 11.2 Hz), 129.2, 128.8 (d, $^{2}J_{C-P}$ = 9.7 Hz), 116.7, 112.9, 61.9 (d, $^{2}J_{C-P}$ = 6.0 Hz), 61.8 (d, $^{2}J_{C-P}$ = 5.0 Hz), 50.4 (d, $^{3}J_{C-P}$ = 3.0 Hz), 31.5 (d, $^{1}J_{C-P}$ = 137.2 Hz), 28.18, 25.5 (d, $^{4}J_{C-P}$ = 3.0 Hz), 17.8, 16.5 (d, $^{3}J_{C-P}$ = 3.0 Hz), 16.4 (d, $^{3}J_{C-P}$ = 3.0 Hz); ^{31}P NMR (121 MHz, $CDCl_3$) δ 28.6; HRMS (ESI+) m/z calcd for $[C_{17}H_{26}NO_3P+H]^+$ 324.1729, found 324.1733.

Diethyl (6-((4-nitrophenyl) amino) cyclohex-1-en-1-yl)methylphosphonate (8b)

Yellow crystal (298 mg, 81% yield, mp 107°C). IR (FT-IR) 3282, 2947, 1608, 1475, 1326, 1237, 1110 cm^{-1}; ^{1}H NMR (300 MHz, $CDCl_3$) δ 8.05 (d, *J* = 9.0 Hz, 2H), 6.60 (d, *J* = 9.0 Hz, 2H), 6.14 (d,

J = 9.0 Hz, 1H), 5.95 - 5.92 (m, 1H), 4.14 - 4.01 (m, 5H), 2.71 (dd, J = 15.0, 24.0 *Hz,* 1H), 2.41 (dd, J = 15.0, 18.0 Hz, 1H), 2.16 - 2.06 (m, 2H), 1.85 - 1.83 (m, 2H), 1.66 - 1.63 (m, 2H), 1.35 - 1.21 (m, 6H); ^{13}C NMR (75 MHz, $CDCl_3$) δ 153.1, 137.1, 131.9 (d, $^{2}J_{C-P}$ = 11.2 Hz), 126.7 (d, $^{3}J_{C-P}$ = 11.2 Hz), 126.3, 110.9, 62.2 (d, $^{2}J_{C-P}$ = 7.5 Hz), 62.0 (d, $^{2}J_{C-P}$ = 6.7 Hz), 51.0 (d, $^{3}J_{C-P}$ = 2.2), 31.5 (d, $^{1}J_{C-P}$ = 137.2 Hz), 28.34, 25.34 (d, $^{4}J_{C-P}$ = 3.0 Hz), 18.09, 16.3 (d, $^{3}J_{C-P}$ = 6.0 Hz), 16.2 (d, $^{3}J_{C-P}$ = 6.0 Hz);); ^{31}P NMR (121 MHz, $CDCl_3$) δ 28.7; HRMS (ESI+) m/z calcd for $[C_{17}H_{25}N_2O_5P+H]^+$ 369.1579, found 369.1582.

Diethyl (6-((4-fluorophenyl) amino)cyclohex-1-en-1-yl)methylphosphonate (8c)

Yellow oil (225 mg, 66% yield). IR (FT-IR) 3333, 2947, 1512, 1229, 1029, 969 cm^{-1}; ^{1}H NMR (300 MHz, $CDCl_3$) δ 6.86-6.80 (m, 2H), 6.59-6.55 (m, 2H), 5.85 (t, J = 3 Hz, 1H), 4.12-3.95 (m, 5H), 2.75 (dd, J = 15 Hz, 1H), 2.45 (dd, J = 15 Hz, 1H), 2.11-2.06 (m, 2H), 1.73-1.67 (m, 2H), 1.63-1.57 (m, 2H), 1.32-1.25 (m, 6H) ; ^{13}C NMR (75 MHz, $CDCl_3$) δ 155.6 (d, $^{1}J_{C-F}$ = 234.6 Hz), 144.2 (d, $^{4}J_{C-F}$ = 1.8 Hz), 130.2 (d, $^{3}J_{C-P}$ = 11.0 Hz), 129.2 (d, $^{2}J_{C-P}$ = 10.4 Hz), 115.4 (d, $^{2}J_{C-F}$ = 22.2 Hz), 113.9 (d, $^{3}J_{C-F}$ = 7.2 Hz), 61.8 (d, $^{2}J_{C-P}$ = 6.0 Hz), 61.7 (d, $^{2}J_{C-P}$ = 6.0 Hz), 51.6 (d, $^{3}J_{C-P}$ = 3.1 Hz), 31.7 (d, $^{1}J_{C-P}$ = 138.2 Hz), 28.4, 25.5 (d, $^{4}J_{C-P}$ = 2.9 Hz), 17.9, 16.3 (d, $^{3}J_{C-P}$ = 2.1 Hz), 16.2 (d, $^{3}J_{C-P}$ = 2.1 Hz); ^{31}P NMR (121 MHz, $CDCl_3$) δ 28.2; ^{19}F NMR (300 MHz, $CDCl_3$) δ -128.7 – -128.8 (m); HRMS (ESI+) m/z calcd for $[C_{17}H_{25}FNO_3P+H]^+$ 342.1634, found 342.1636.

Diethyl (6-(naphthalen-2-ylamino) cyclohex-1-en-1-yl)methylphosphonate (8d)

Yellow oil (149 mg, 40% yield). IR (FT-IR) 3378, 2947, 1594, 1542, 1415, 1237, 1029, 984, 783 cm^{-1}; ^{1}H NMR (300 MHz, $CDCl_3$) δ 8.01-7.98 (m, 1H), 7.75-7.72 (m, 1H), 7.40-7.14 (m, 4H), 6.66 (d, J = 7.6 Hz, 1H), 5.96 (t, J = 3 Hz, 1H), 4.25-3.99 (m, 5H), 2.77 (dd, J = 22.3, 15.1 Hz, 1H), 2.56 (dd, J = 20.7, 15.1 Hz, 1H), 2.15-1.94 (m, 3H), 1.88-1.80 (m, 1H), 1.68-1.61 (m, 2H), 1.31-1.19 (m, 6H); ^{13}C NMR (75 MHz, $CDCl_3$) δ 143.0, 134.9, 130.7 (d, $^{3}J_{C-P}$ = 10.8 Hz), 129.3 (d, $^{2}J_{C-P}$ = 10.5 Hz), 128.5, 126.6, 125.6, 124.5), 123.9, 120.8, 116.7, 104.2, 62.0 (d, $^{2}J_{C-P}$ = 6.6 Hz), 61.9 (d, $^{2}J_{C-P}$ = 6.9 Hz), 51.1 (d, $^{3}J_{C-P}$ = 3.2 Hz), 31.8 (d, $^{1}J_{C-P}$ = 138.2 Hz), 28.2, 25.8 (d, $^{4}J_{C-P}$ = 3.0 Hz), 18.7, 16.4 (d, $^{3}J_{C-P}$ = 5.8 Hz, 16.4 (d, $^{3}J_{C-P}$ = 5.6 Hz); ^{31}P NMR (121 MHz, $CDCl_3$) δ 28.6; HRMS (ESI+) m/z calcd for $[C_{21}H_{28}NO_3P+H]^+$ 374.1885, found 374.1883.

Reference

1. Rezgui, F.; El Gaïed, M. M. *J. Chem. Res., Synop.* **1999,** 510–511. doi:10.1039/A902012H
