# Supplementary material for: First DMAP-mediated direct conversion of Morita–Baylis–Hillman alcohols into γ-ketoallylphosphonates: Synthesis of γ-aminoallylphosphonates
Source: Beilstein J Org Chem. 2016 Dec 30;12:2906–15. doi: 10.3762/bjoc.12.290 (PMC5238584; doi:10.3762/bjoc.12.290)
Supplement: File 2 — Spectral data for synthesized compounds. [file Beilstein_J_Org_Chem-12-2906-s002.pdf]

Supporting Information File 2

for

First DMAP-mediated direct conversion of Morita–Baylis–Hillman alcohols into γ-ketoallylphosphonates: Synthesis of γ-aminoallylphosphonates

Marwa Ayadi[1,2], Haitham Elleuch[1], Emmanuel Vrancken[2], and Farhat Rezgui*[1]

Address: [1]Université de Tunis El Manar, Faculté des Sciences de Tunis, Laboratoire de Chimie Organique Structurale LR99ES14, Campus Universitaire, 2092 Tunis, Tunisie and [2]Institut Charles Gerhardt UMR 5253 CNRS-UM-ENSCM, 8 rue de l'Ecole Normale 34296 Montpellier Cedex 5, France.

Email: Farhat Rezgui* - rez_far@yahoo.fr

*Corresponding author

Spectral data for synthesized compounds

Table of Contents:

- ^{1}H NMR (300 MHz, $CDCl_3$)
- ^{13}C NMR (75 MHz, $CDCl_3$)
- ^{31}P NMR (121 MHz, $CDCl_3$)
- IR (FT-IR)
- HRMS (ESI+)

Ethyl 2-(diphenoxyphosphoryl)methylacrylate (4c)

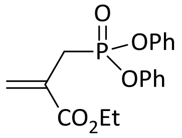

4c

^{1}H NMR (300 MHz, $CDCl_3$):

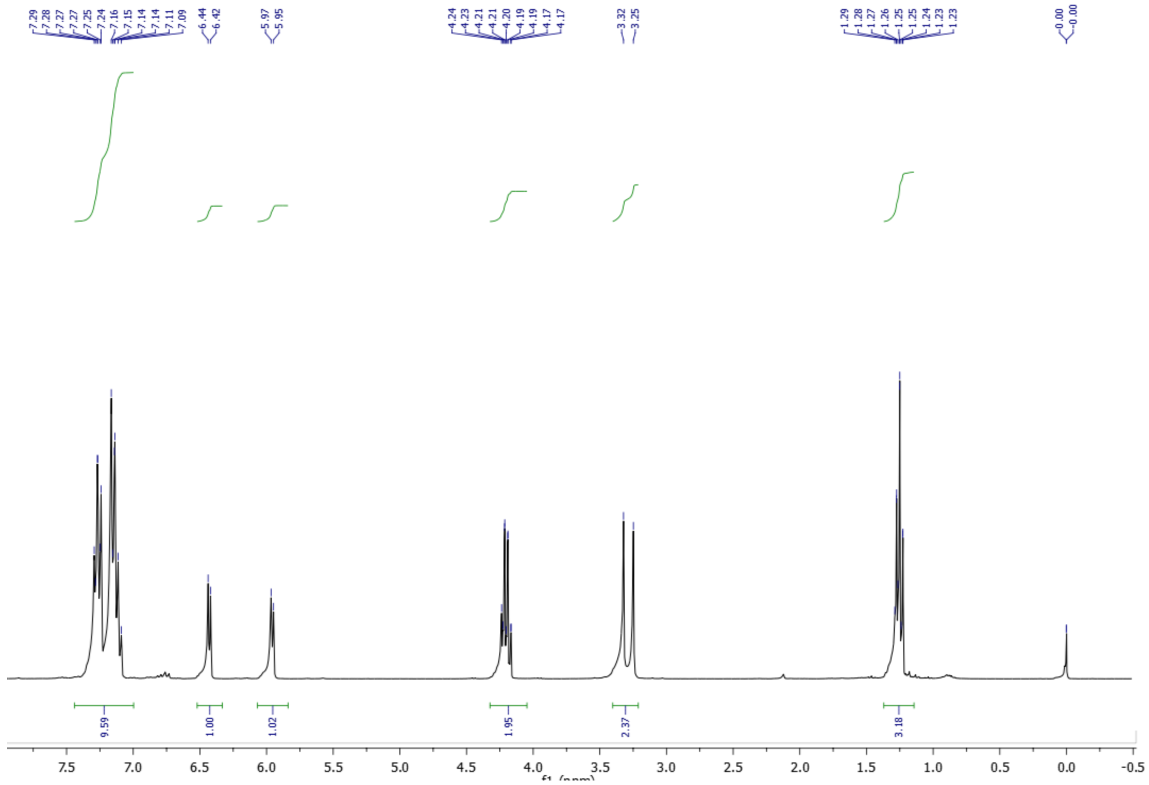

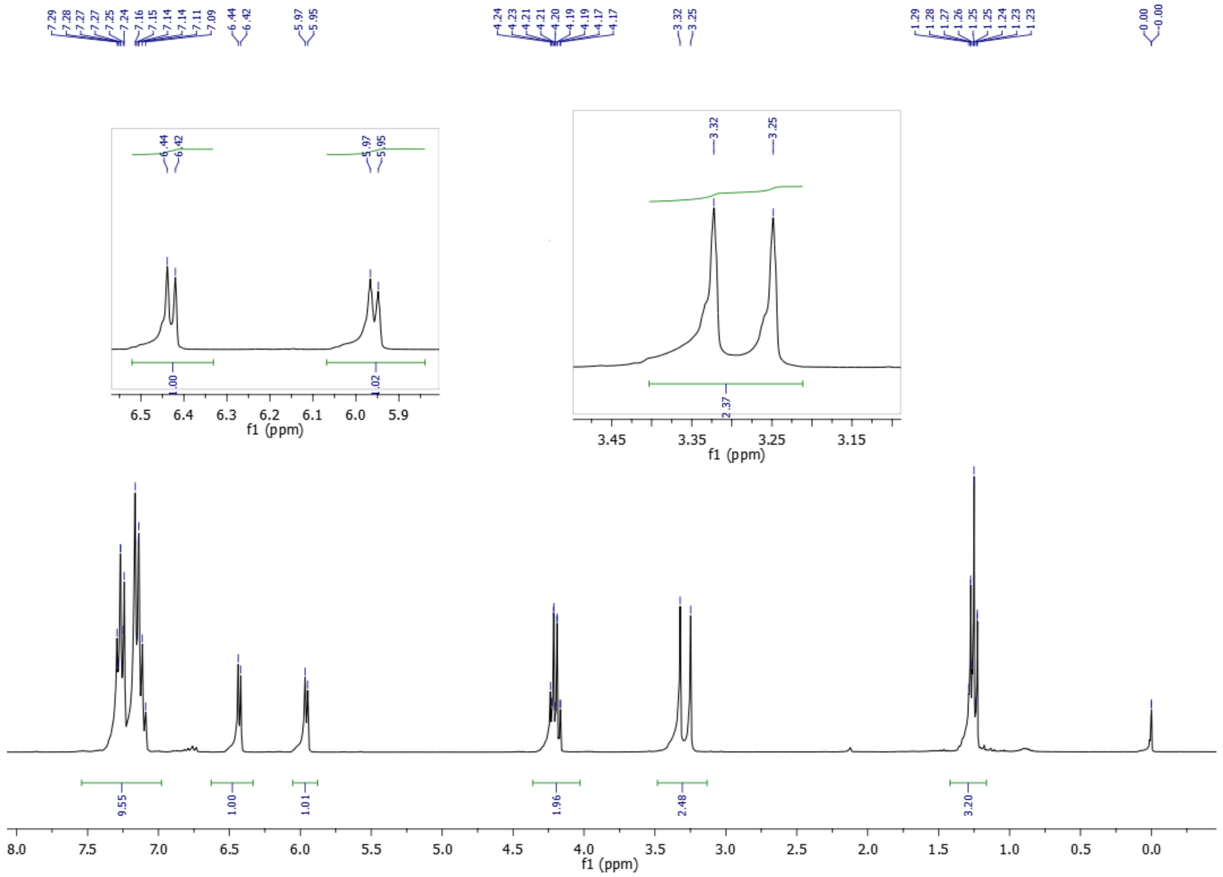

^{13}C NMR (75 MHz, $CDCl_3$):

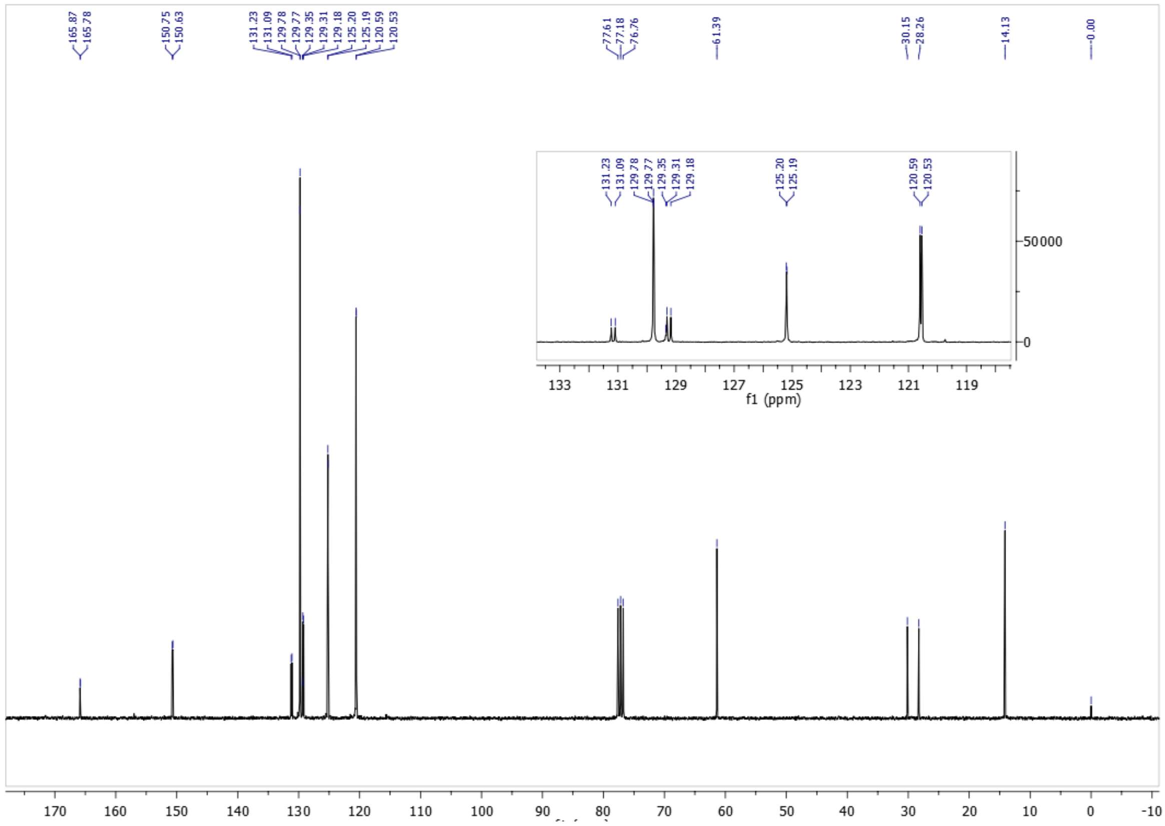

^{31}P NMR (121 MHz, $CDCl_3$):

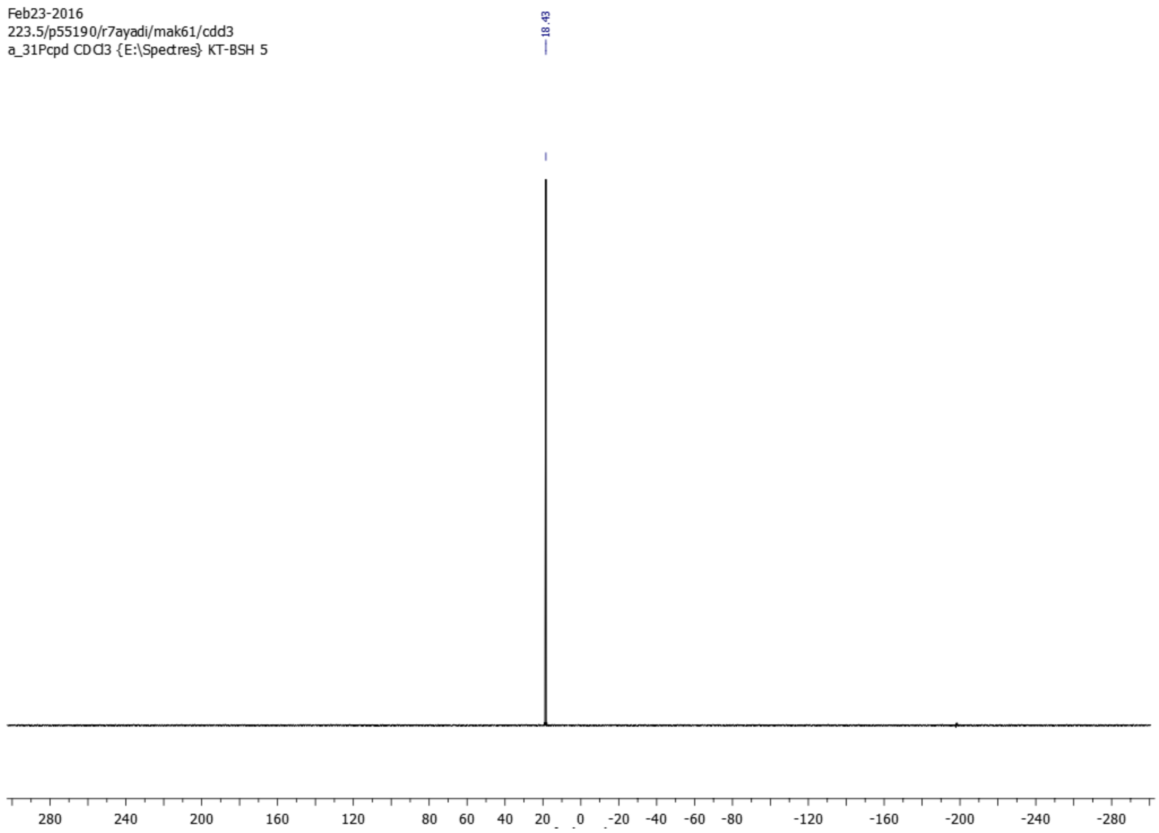

IR (FT-IR):

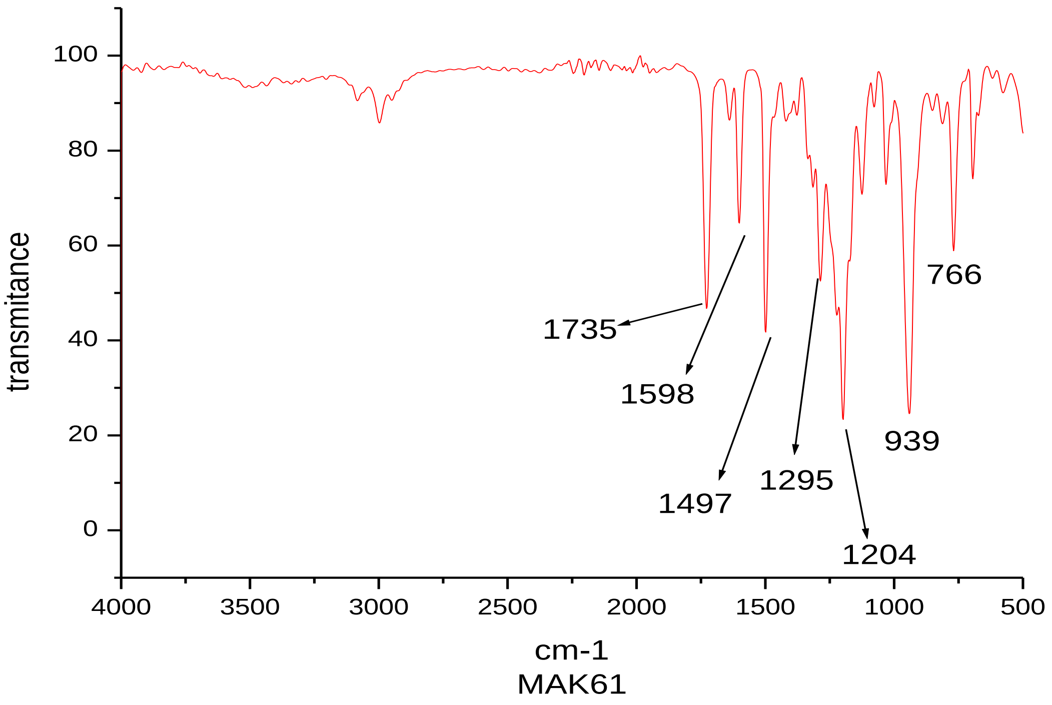

HRMS (ESI+):

Single Mass Analysis
Tolerance = 1.0 PPM / DBE: min = -10.0, max = 50.0
Element prediction: Off
Number of isotope peaks used for i-FIT = 3

Monoisotopic Mass, Even Electron Ions
938 formula(e) evaluated with 1 results within limits (up to 50 best isotopic matches for each mass)
Elements Used:
C: 0-100 H: 0-100 N: 0-20 O: 0-20 P: 1-1

SYNAPT G2-S#UEB205 MAK61 17-May-2016
Y-JOM16051703 3 (0.141) Cm (3:4) 1: TOF MS ES+
6.73e+007

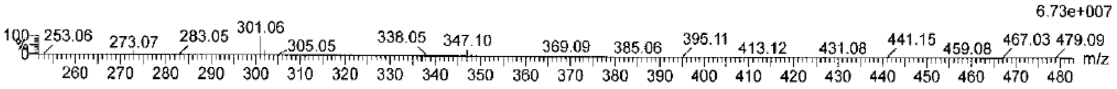

Minimum:				-10.0				
Maximum:		1.0	1.0	50.0				
Mass	Calc. Mass	mDa	PPM	DBE	i-FIT	Norm	Conf(%)	Formula
347.1049	347.1048	0.1	0.3	9.5	3197.6	n/a	n/a	C18 H20 O5 P

Diethyl (6-hydroxycyclohex-1-en-1-yl)methyl phosphonate (5)

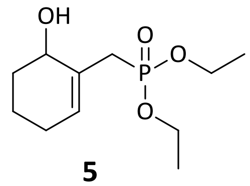

5

^{1}H NMR (300 MHz, CDCl$_3$):

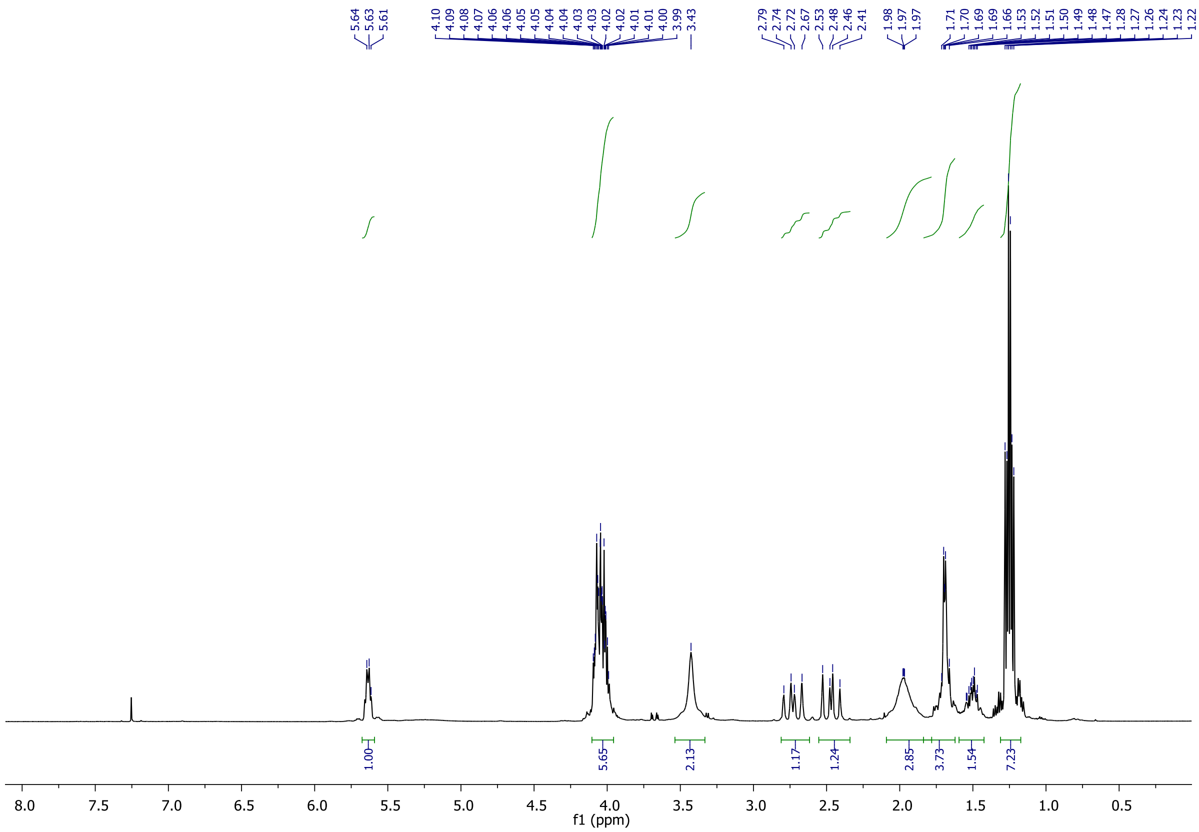

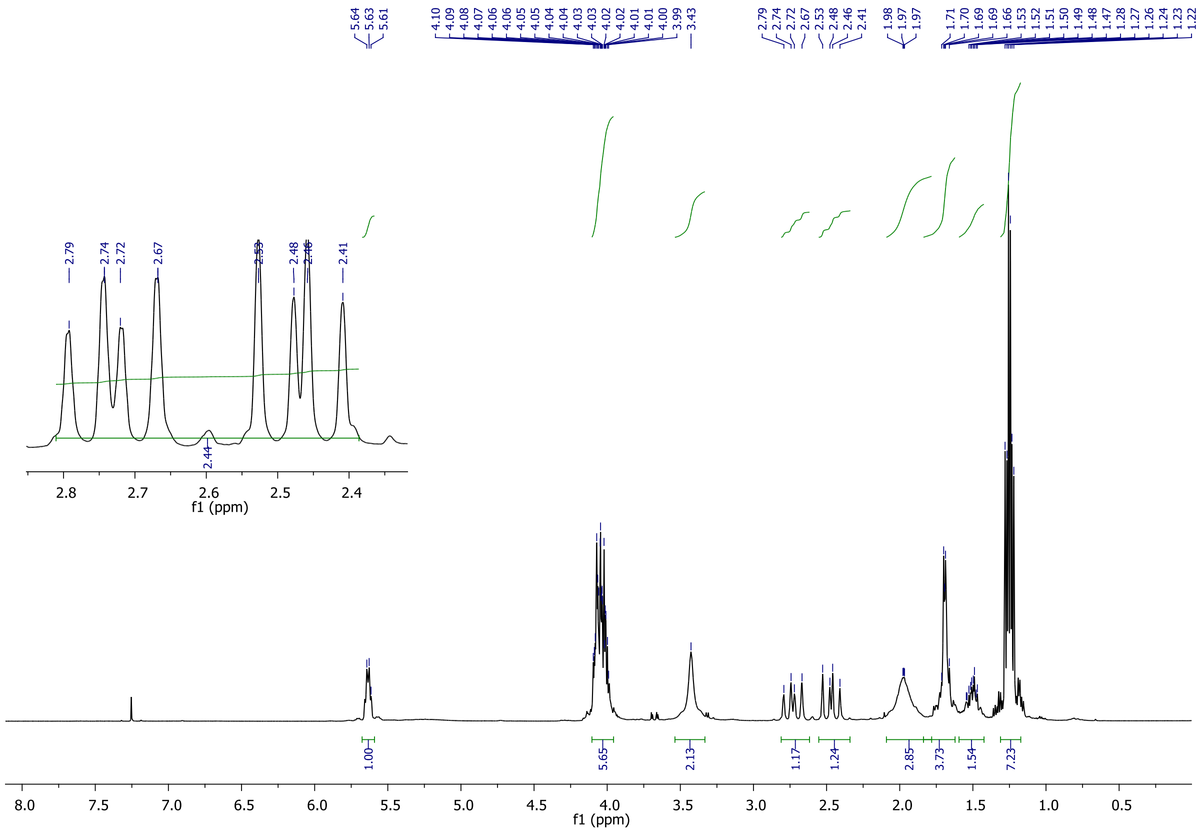

^{13}C NMR (75 MHz, $CDCl_3$):

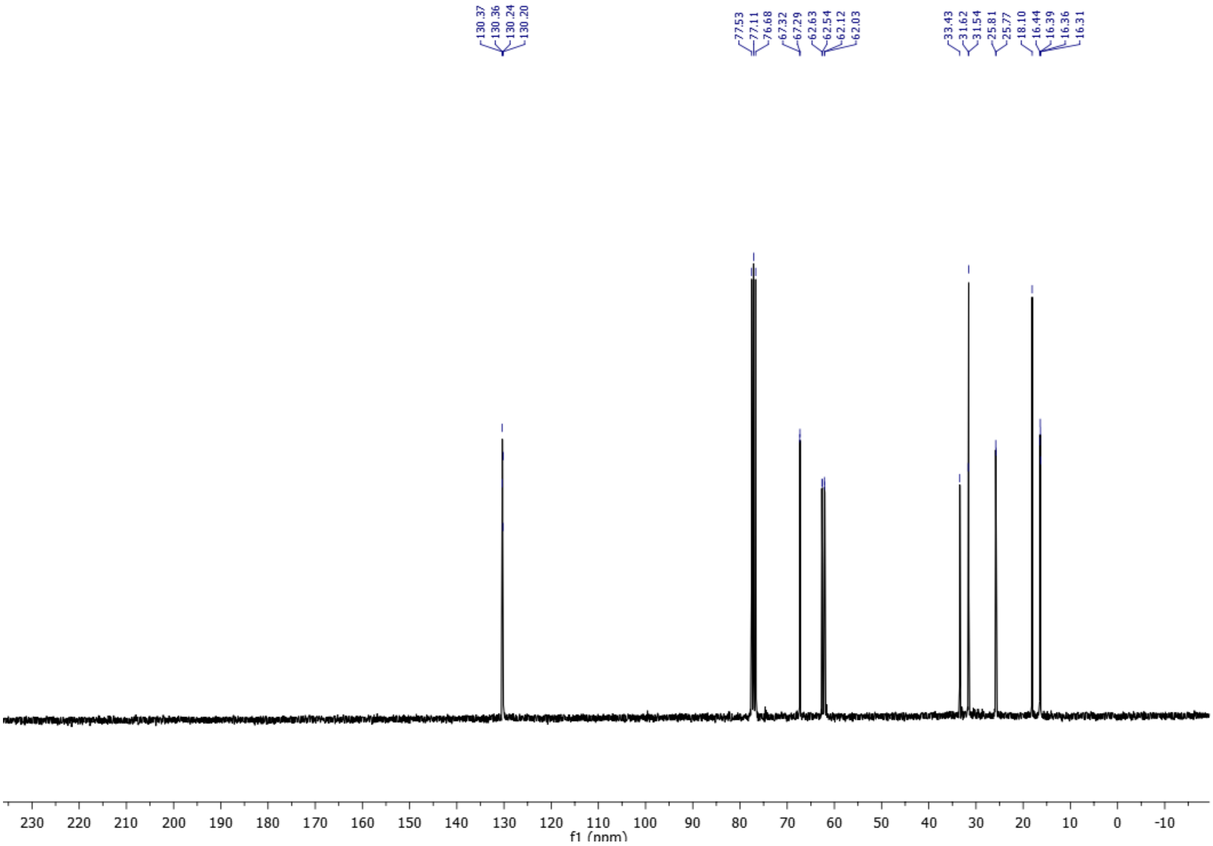

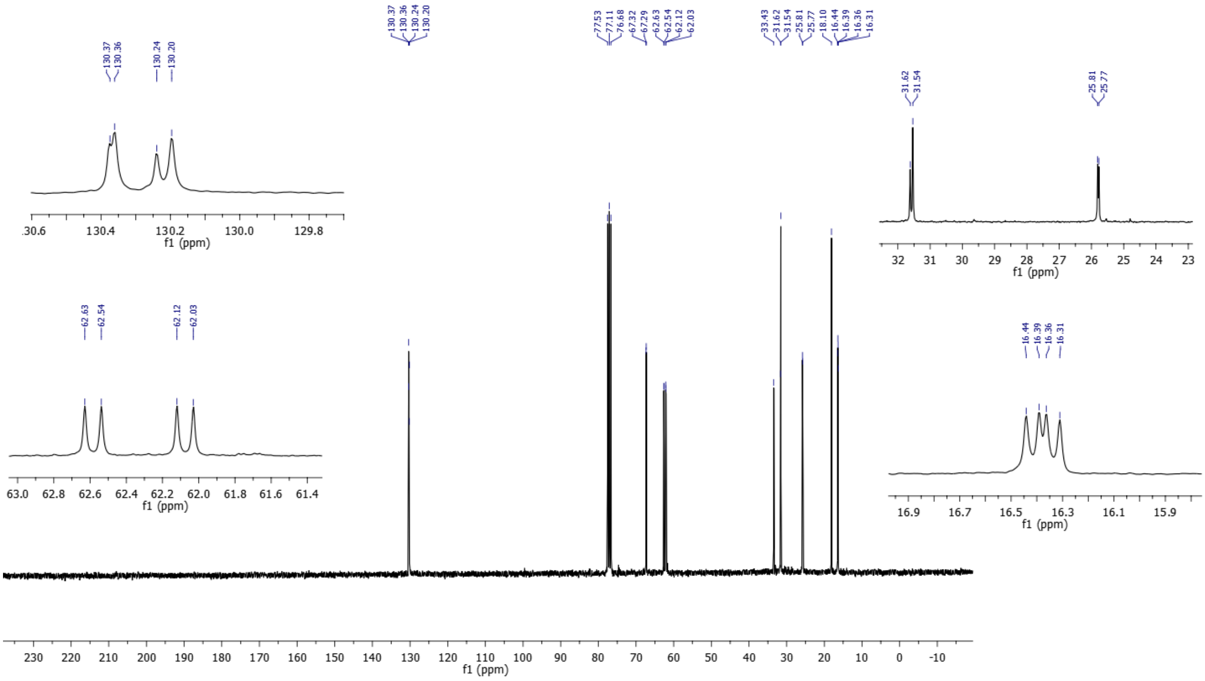

^{31}P NMR (121 MHz, $CDCl_3$):

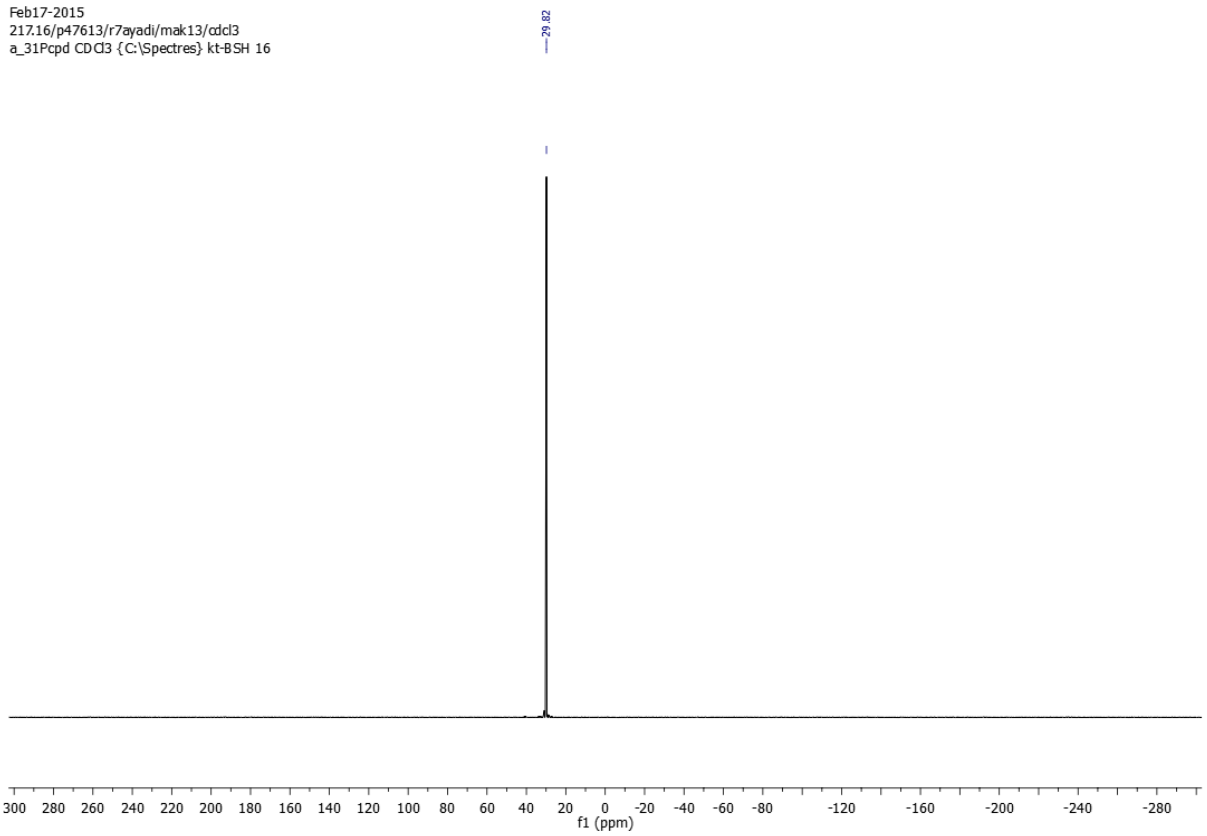

IR (FT-IR):

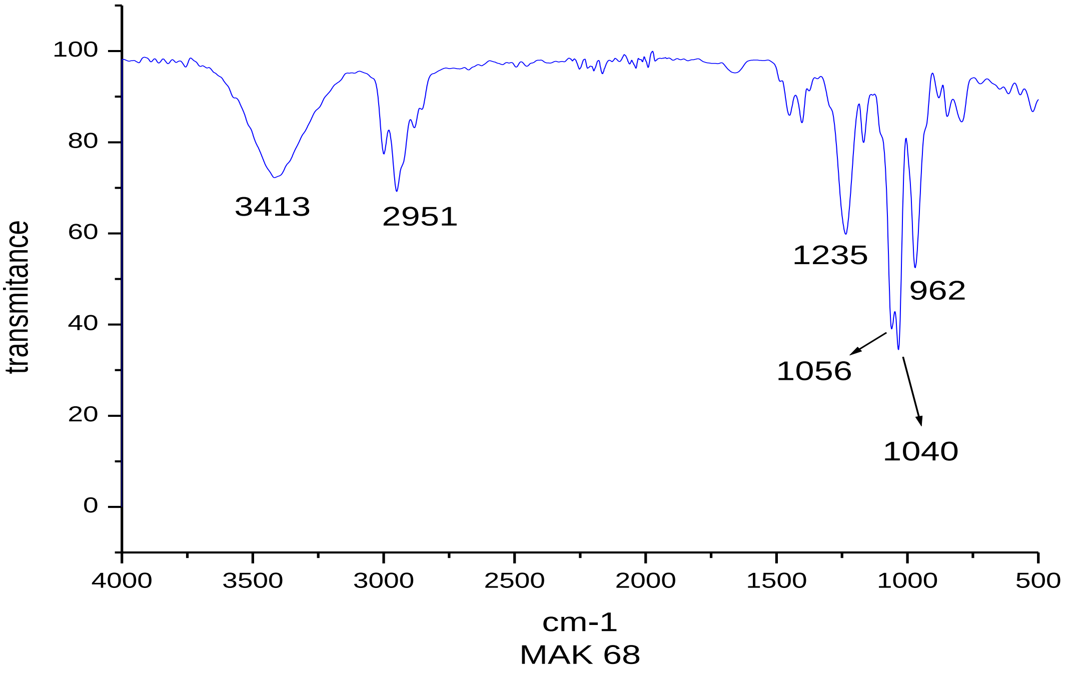

HRMS (ESI+):

Single Mass Analysis
Tolerance = 1.0 PPM / DBE: min = -10.0, max = 50.0
Element prediction: Off
Number of isotope peaks used for i-FIT = 3

Monoisotopic Mass, Even Electron Ions
892 formula(e) evaluated with 2 results within limits (up to 50 best isotopic matches for each mass)
Elements Used:
C: 0-100 H: 0-100 N: 0-20 O: 0-20 P: 1-1 Na: 0-1

SYNAPT G2-S#UEB205 MAK68 17-May-2016
Y-JOM16051705 3 (0.141) Cm (3:4) 1: TOF MS ES+
1.00e+007

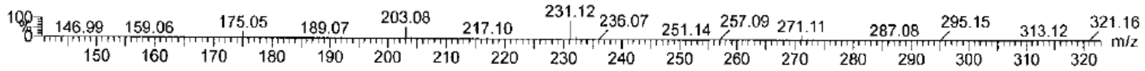

Mass	Calc. Mass	mDa	PPM	DBE	i-FIT	Norm	Conf(%)	Formula
Minimum:				-10.0				
Maximum:		1.0	1.0	50.0				
271.1075	271.1075	0.0	0.0	1.5	2958.9	0.445	64.08	C11 H21 O4 P Na
	271.1072	0.3	1.1	5.5	2959.5	1.024	35.92	C9 H16 N6 O2 P

Diethyl ((6-(phenylsulfonamido)cyclohex-1-en-1-yl)methyl)phosphonate (6a)

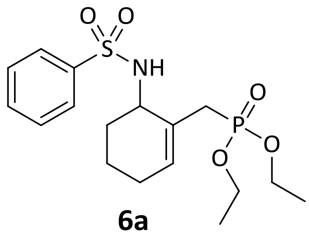

6a

^{1}H NMR (300 MHz, CDCl$_3$):

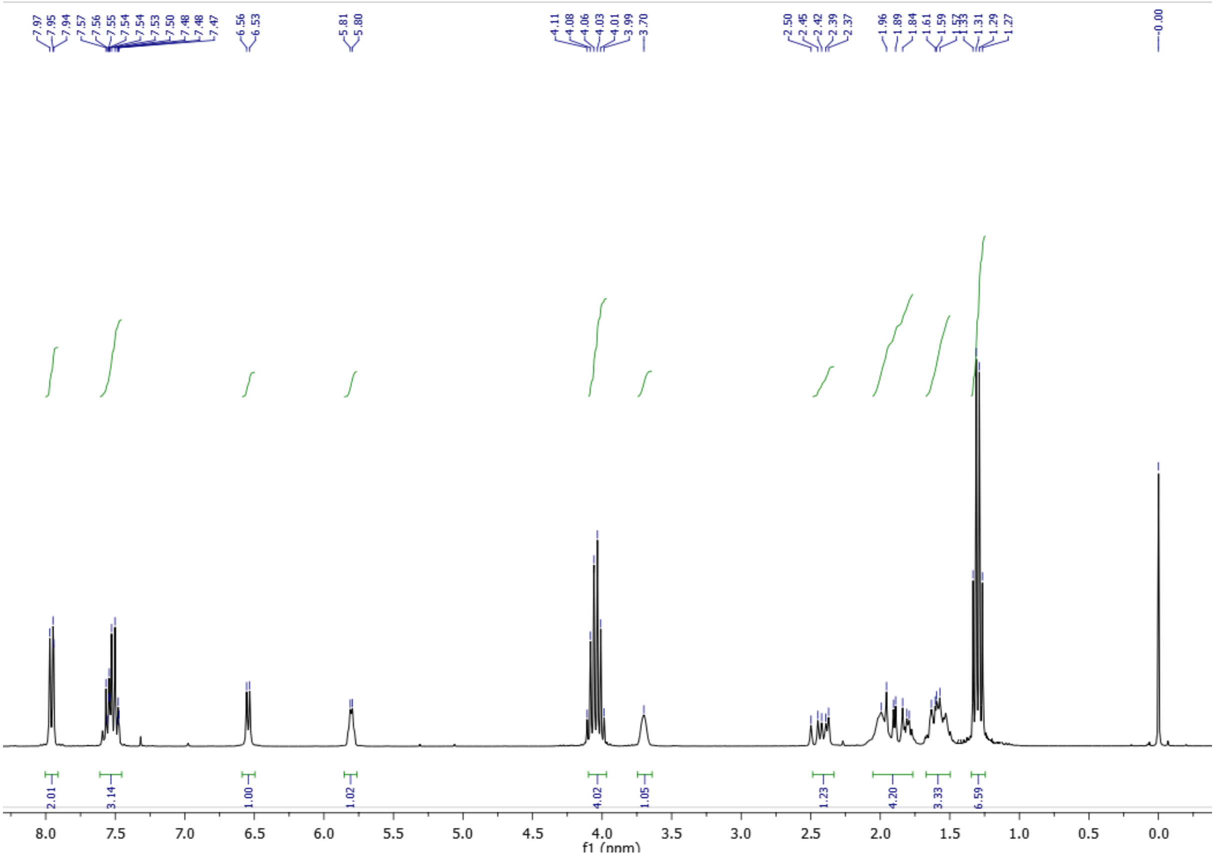

^{13}C NMR (75 MHz, $CDCl_3$):

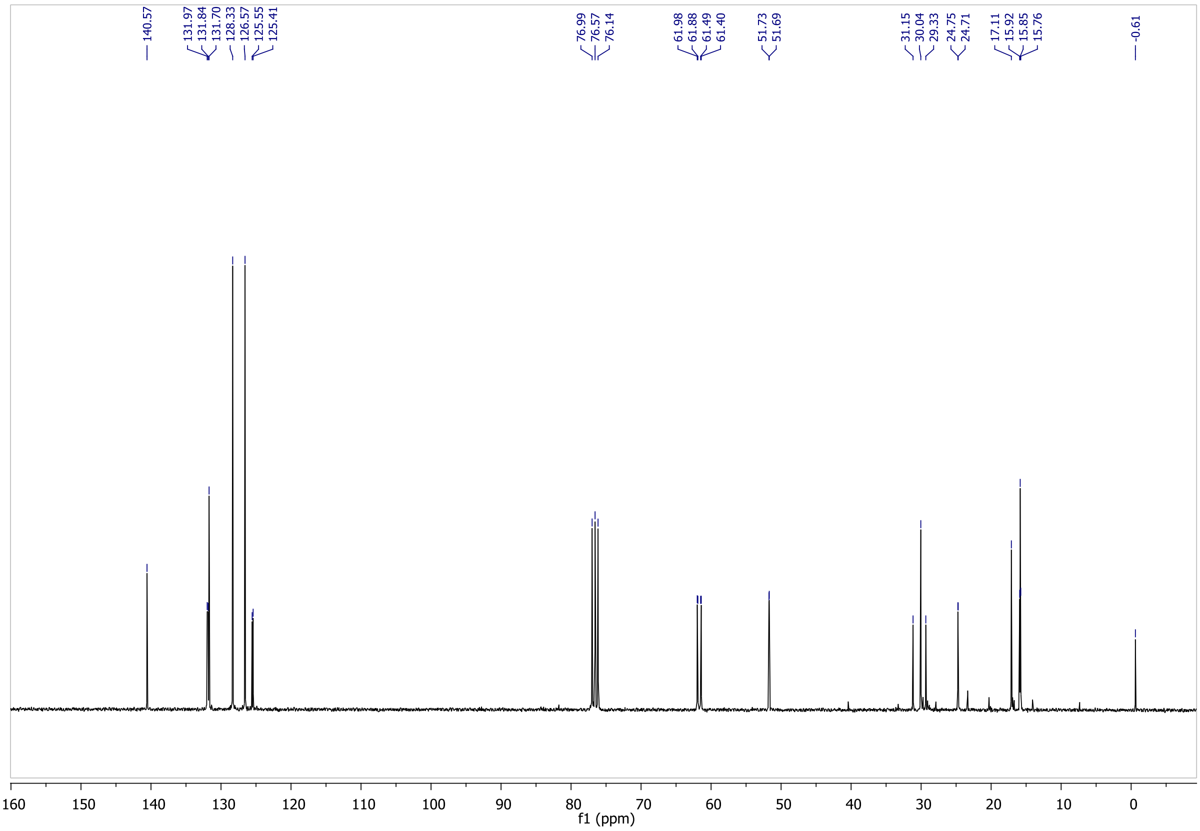

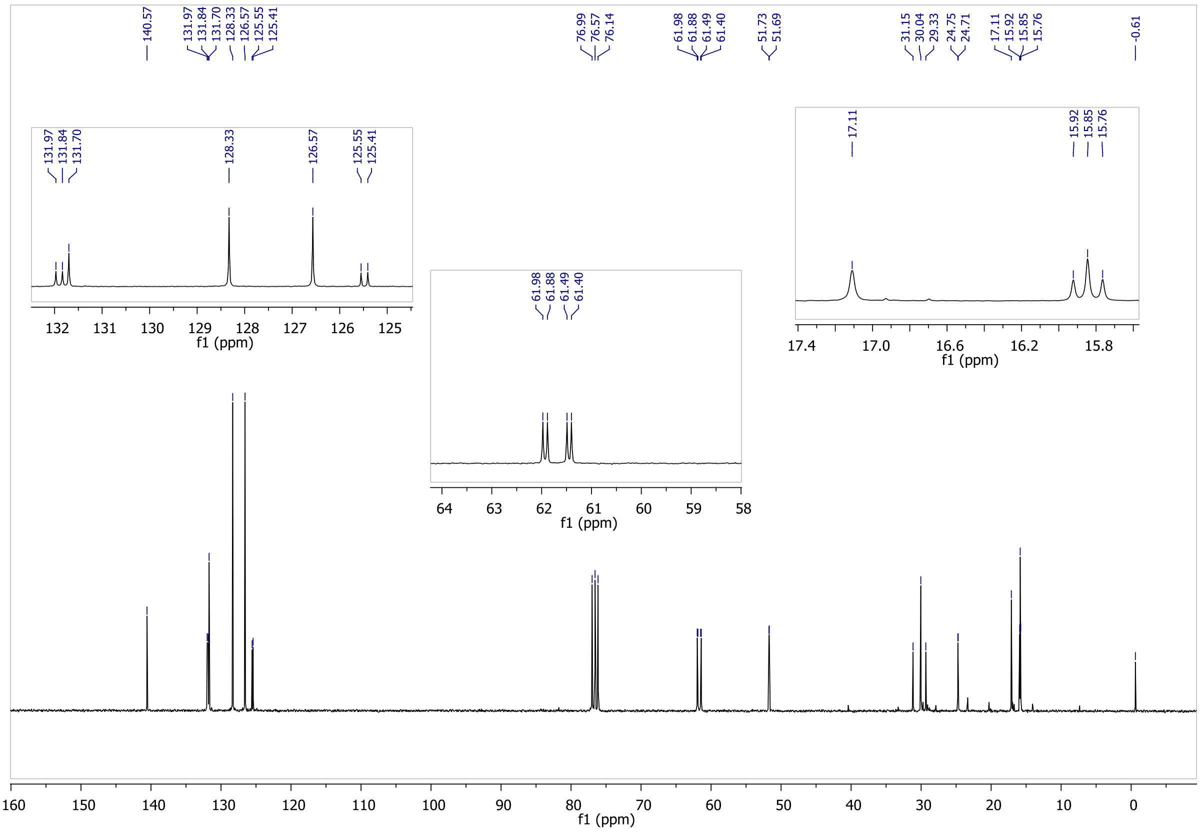

^{31}P NMR (121 MHz, $CDCl_3$):

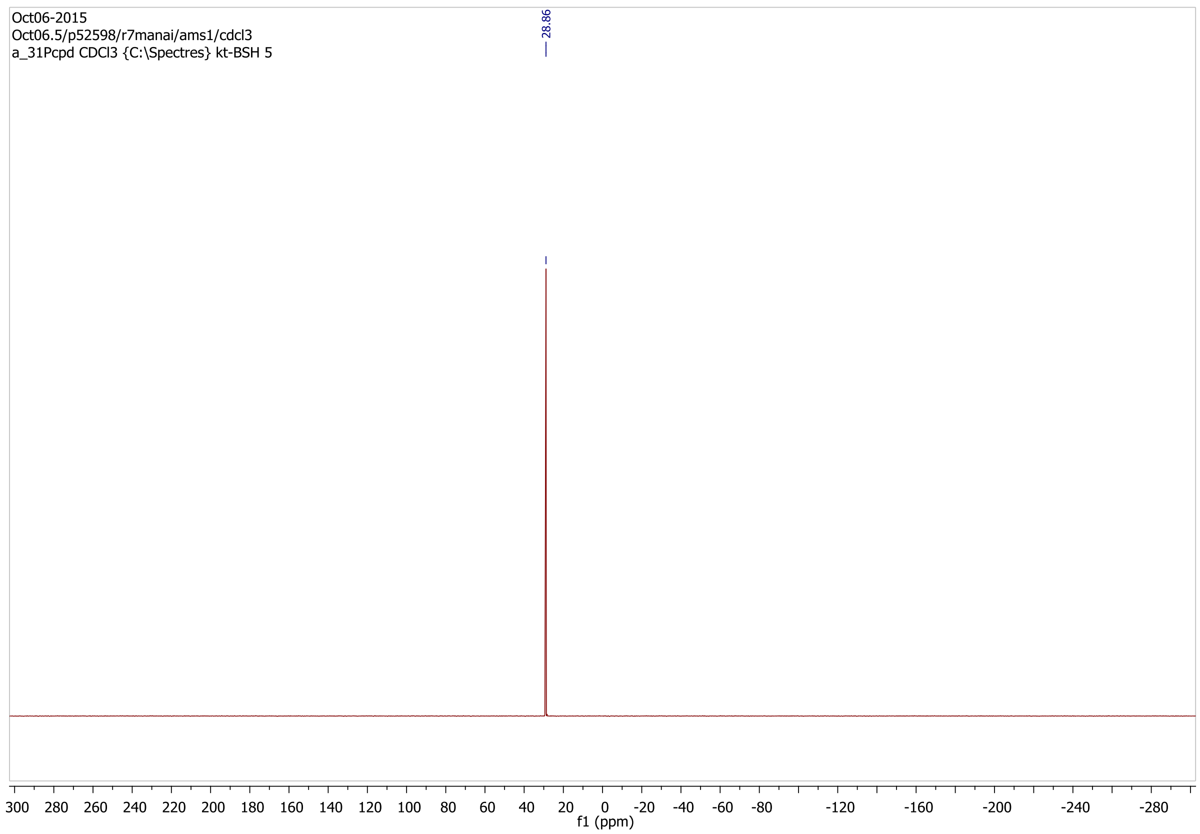

IR (FT-IR):

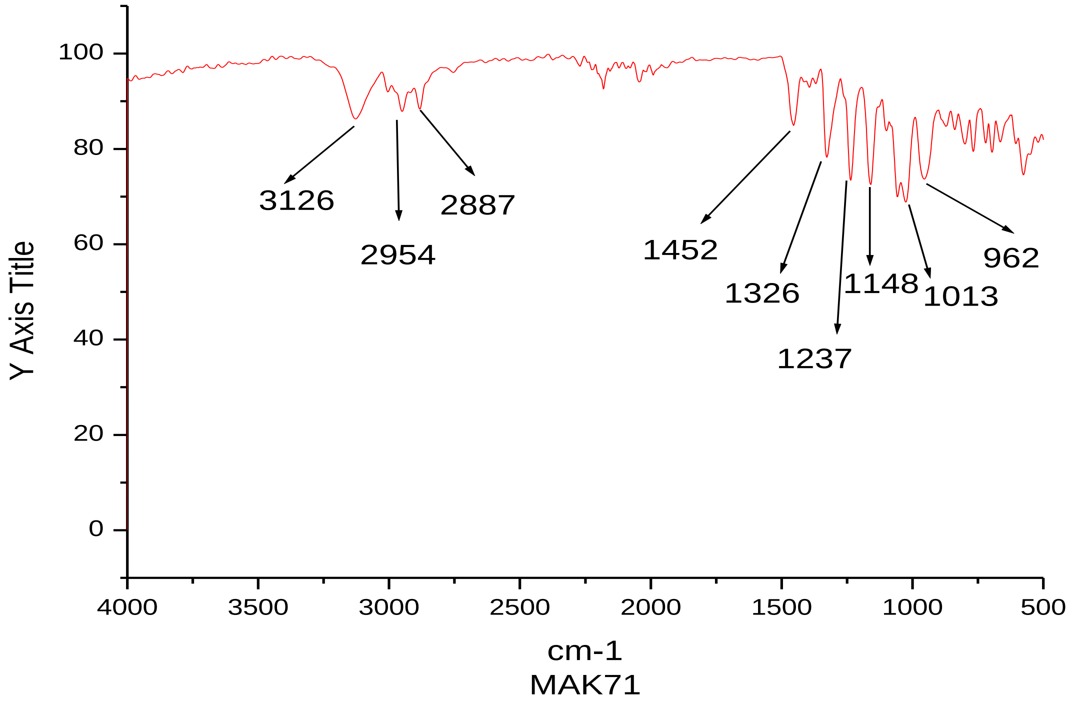

HRMS (ESI+):

Elemental Composition Report **Page 1**

Single Mass Analysis
Tolerance = 1.0 PPM / DBE: min = -10.0, max = 50.0
Element prediction: Off
Number of isotope peaks used for i-FIT = 3

Monoisotopic Mass, Even Electron Ions
1022 formula(e) evaluated with 1 results within limits (up to 50 best isotopic matches for each mass)
Elements Used:
C: 0-100 H: 0-100 N: 0-20 O: 0-20 S: 1-1 P: 1-1

SYNAPT G2-S#UEB205 MAK71 17-May-2016
Y-JOM16051709 3 (0.141) Cm (3) 1: TOF MS ES+
1.04e+006

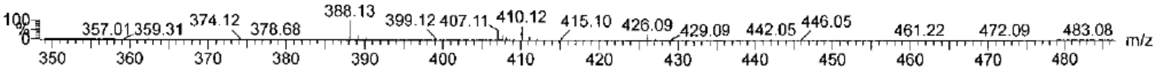

Minimum:				-10.0				
Maximum:		1.0	1.0	50.0				
Mass	Calc. Mass	mDa	PPM	DBE	i-FIT	Norm	Conf(%)	Formula
388.1346	388.1348	-0.2	-0.5	5.5	1793.5	n/a	n/a	C17 H27 N O5 S P

Diethyl ((6-(4-methylphenylsulfonamido)cyclohex-1-en-1-yl)methyl)phosphonate (6b)

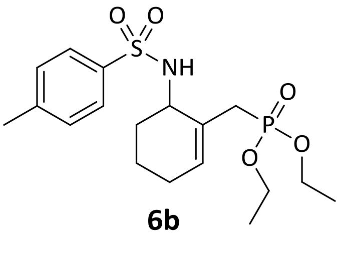

6b

^{1}H NMR (300 MHz, $CDCl_3$):

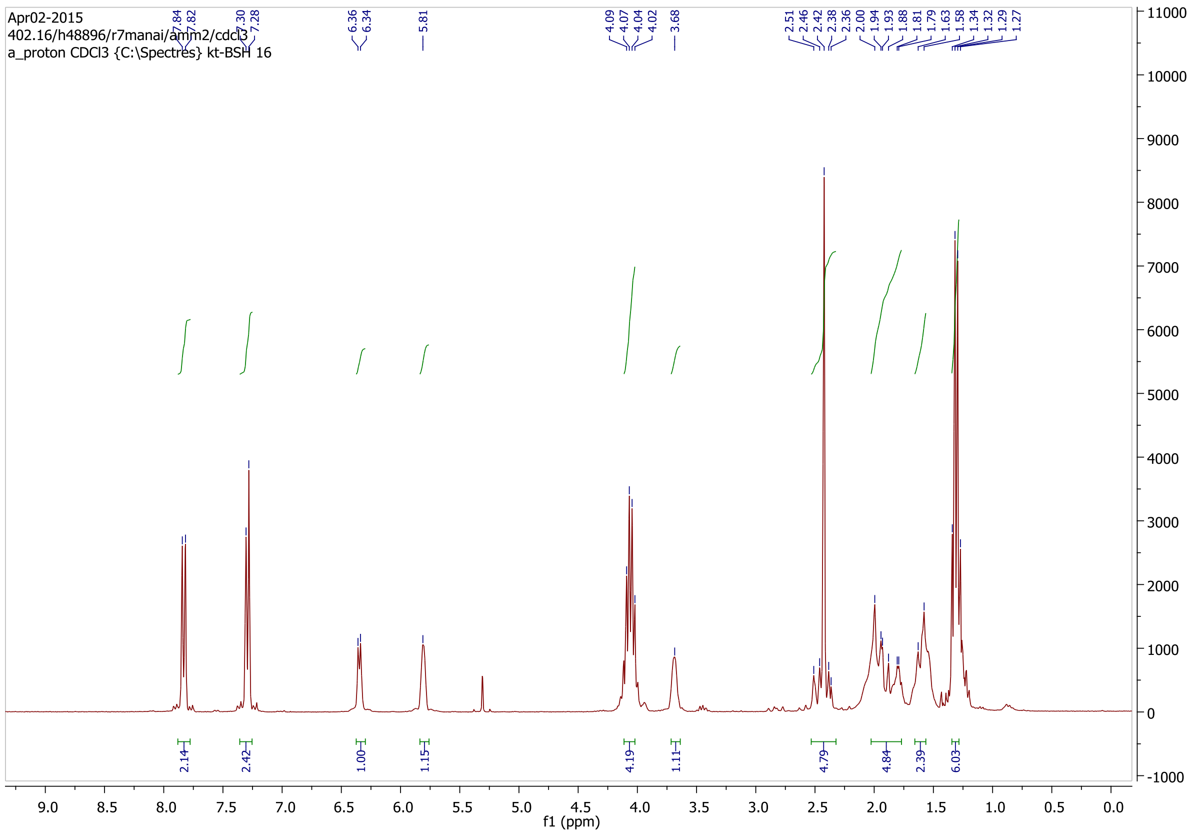

^{13}C NMR (75 MHz, $CDCl_3$):

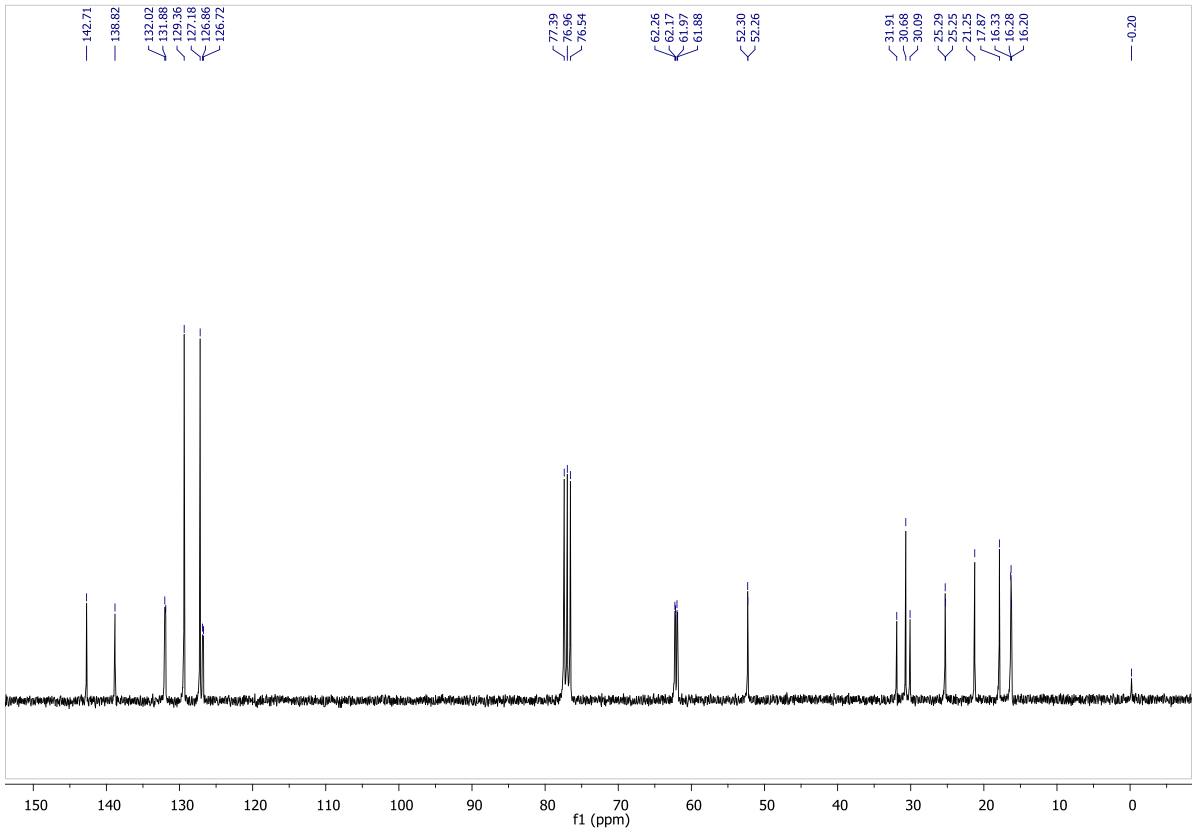

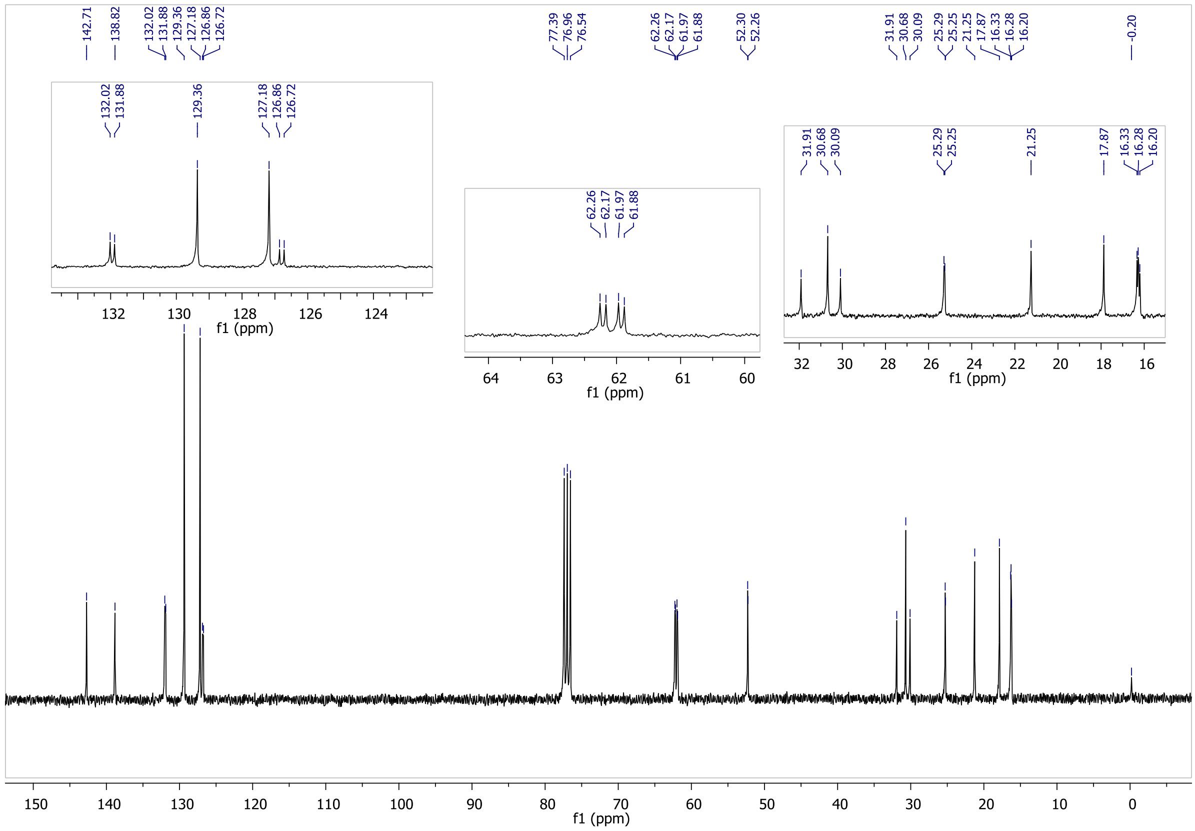

^{31}P NMR (121 MHz, $CDCl_3$):

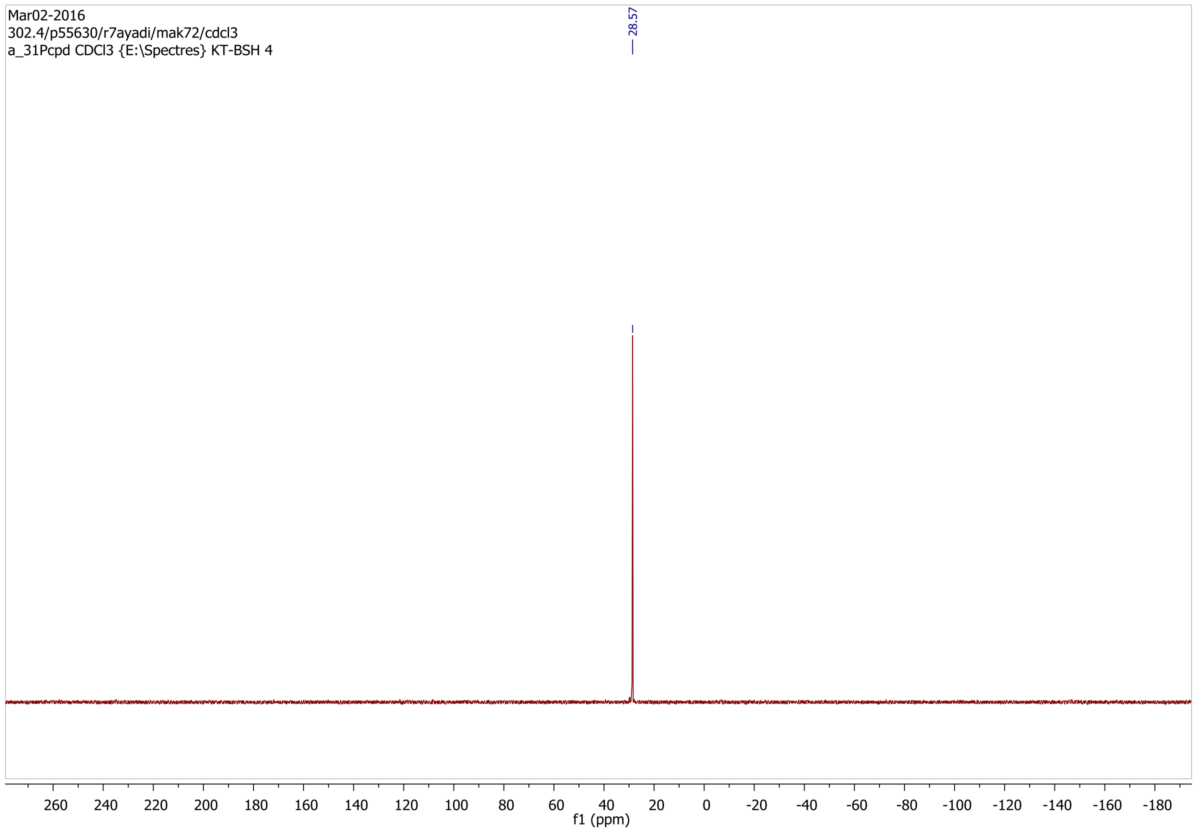

IR (FT-IR):

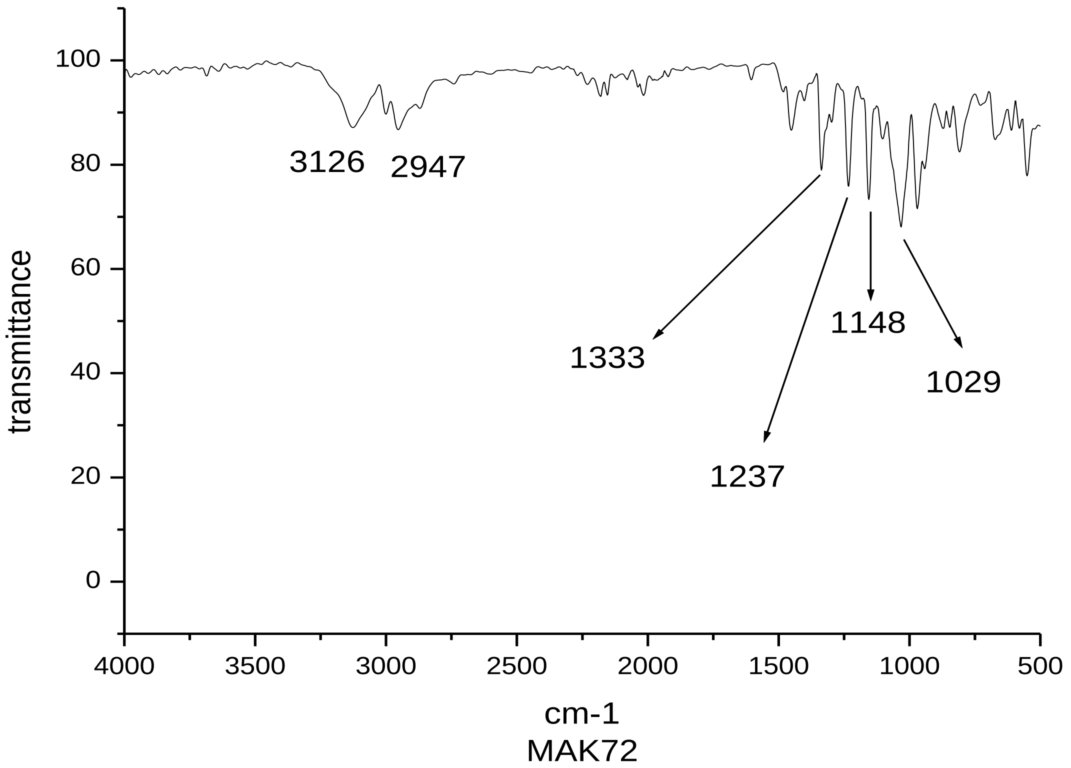

HRMS (ESI+):

Elemental Composition Report **Page 1**

Single Mass Analysis
Tolerance = 1.0 PPM / DBE: min = -10.0, max = 50.0
Element prediction: Off
Number of isotope peaks used for i-FIT = 3

Monoisotopic Mass, Even Electron Ions
1123 formula(e) evaluated with 1 results within limits (up to 50 best isotopic matches for each mass)
Elements Used:
C: 0-100 H: 0-100 N: 0-20 O: 0-20 S: 1-1 P: 1-1

SYNAPT G2-S#UEB205 MAK72 17-May-2016
Y-JOM16051708 3 (0.141) Cm (3) 1: TOF MS ES+
9.32e+005

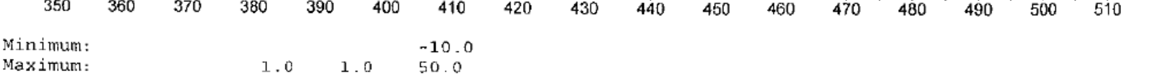

Minimum:				-10.0				
Maximum:		1.0	1.0	50.0				
Mass	Calc. Mass	mDa	PPM	DBE	i-FIT	Norm	Conf(%)	Formula
402.1501	402.1504	-0.3	-0.7	5.5	1676.5	n/a	n/a	C18 H29 N O5 S P

Diethyl ((6-(4-bromophenylsulfonamido)cyclohex-1-en-1-yl)methyl)phosphonate (6c)

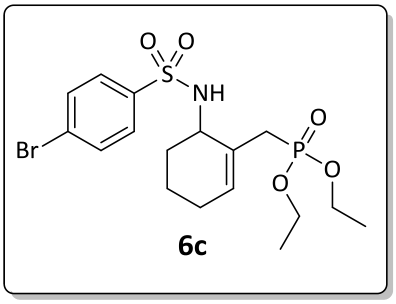

^{1}H RMN (300 MHz, $CDCl_3$) :

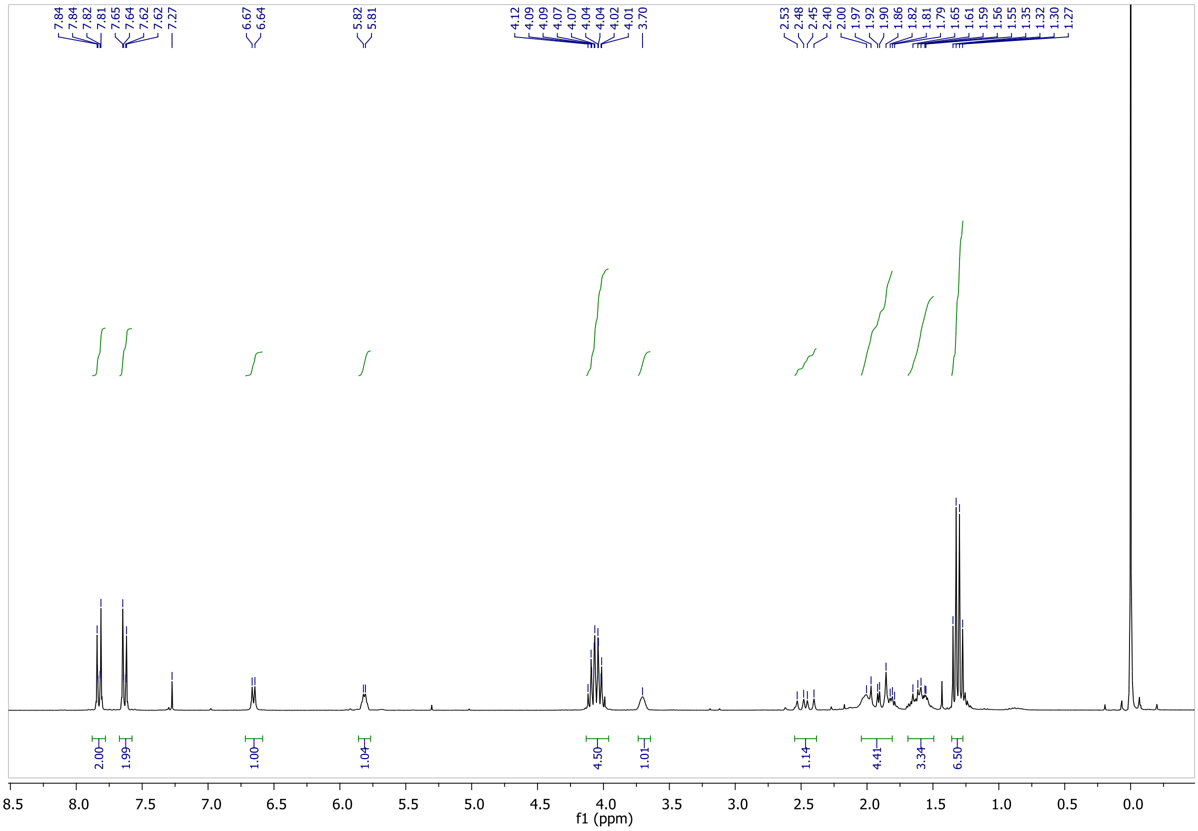

^{13}C NMR (75 MHz, CDCl$_3$) :

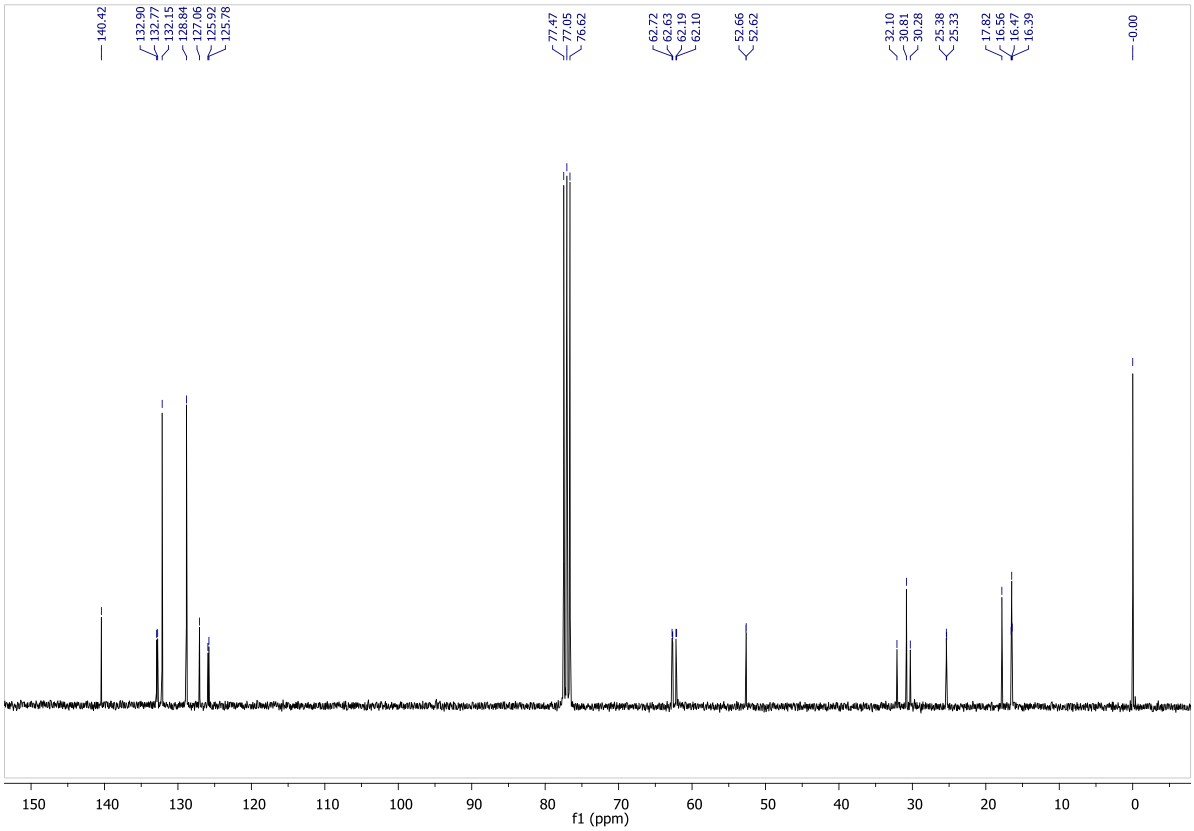

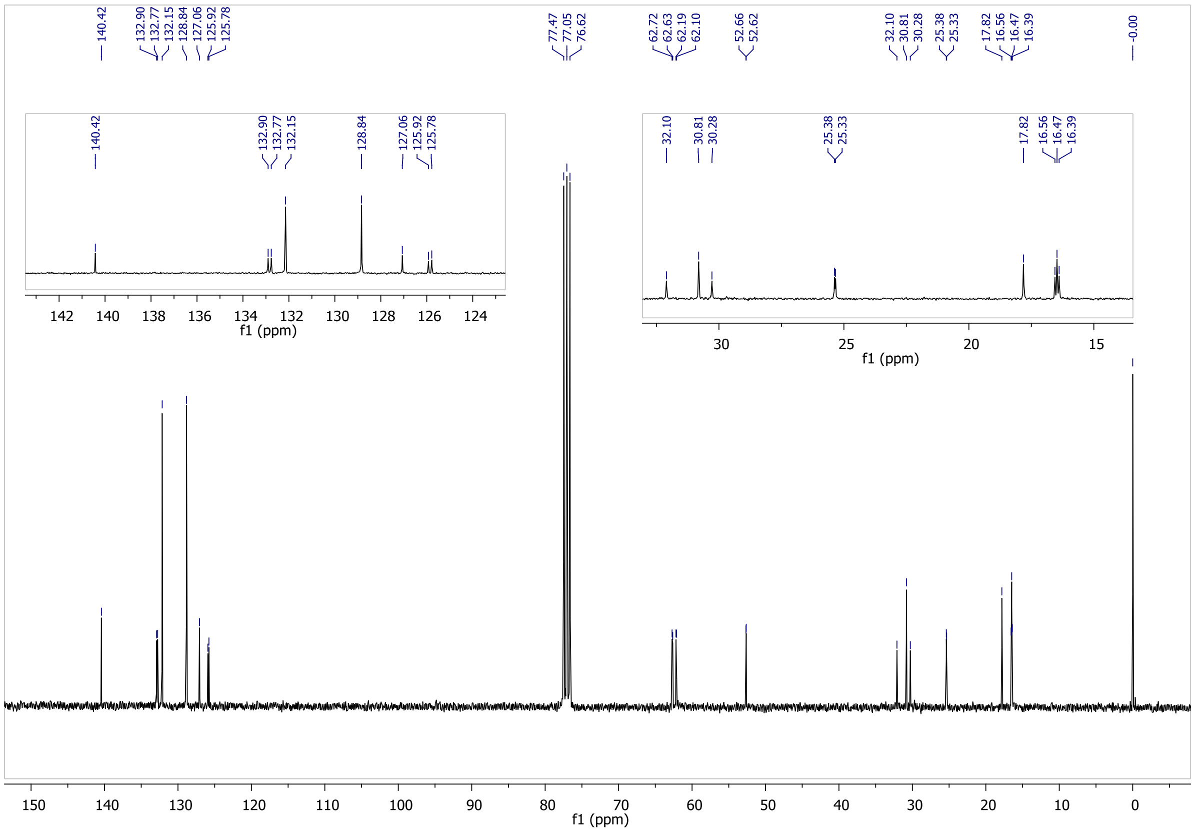

^{31}P NMR (121 MHz, $CDCl_3$):

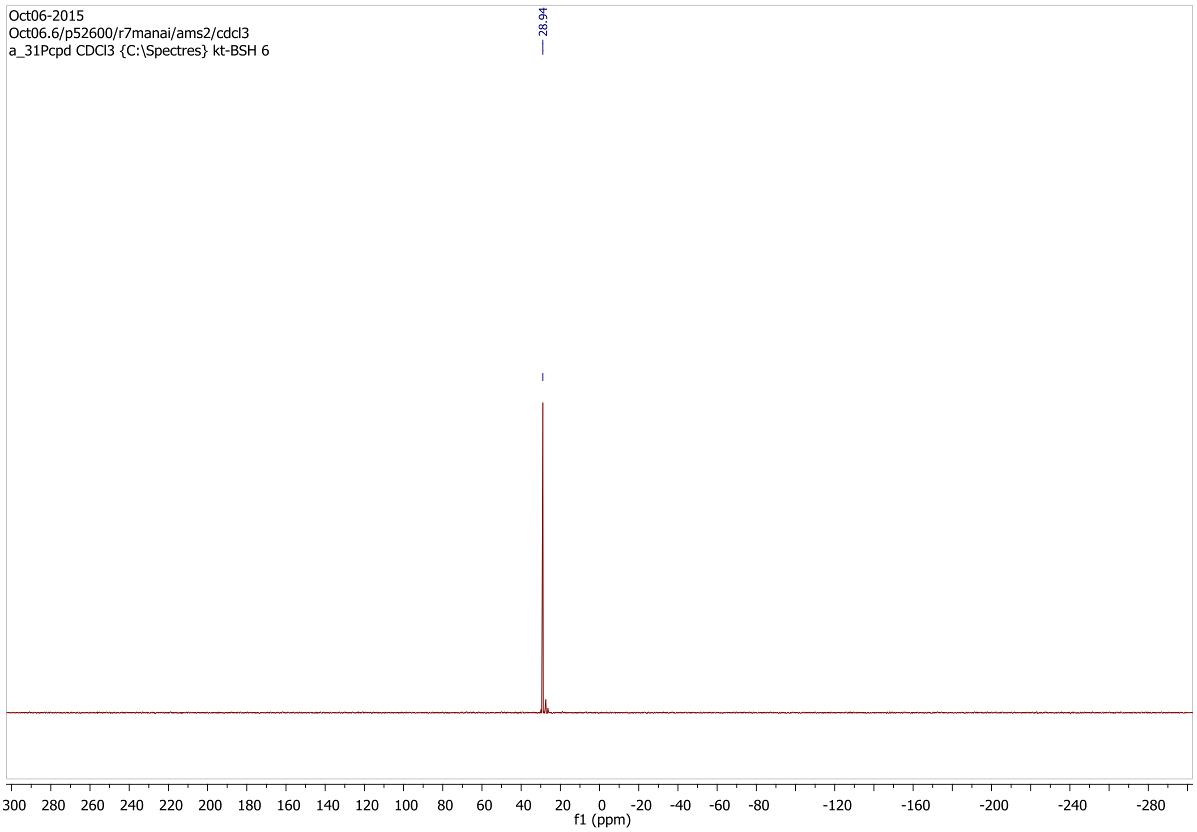

IR (FT-IR):

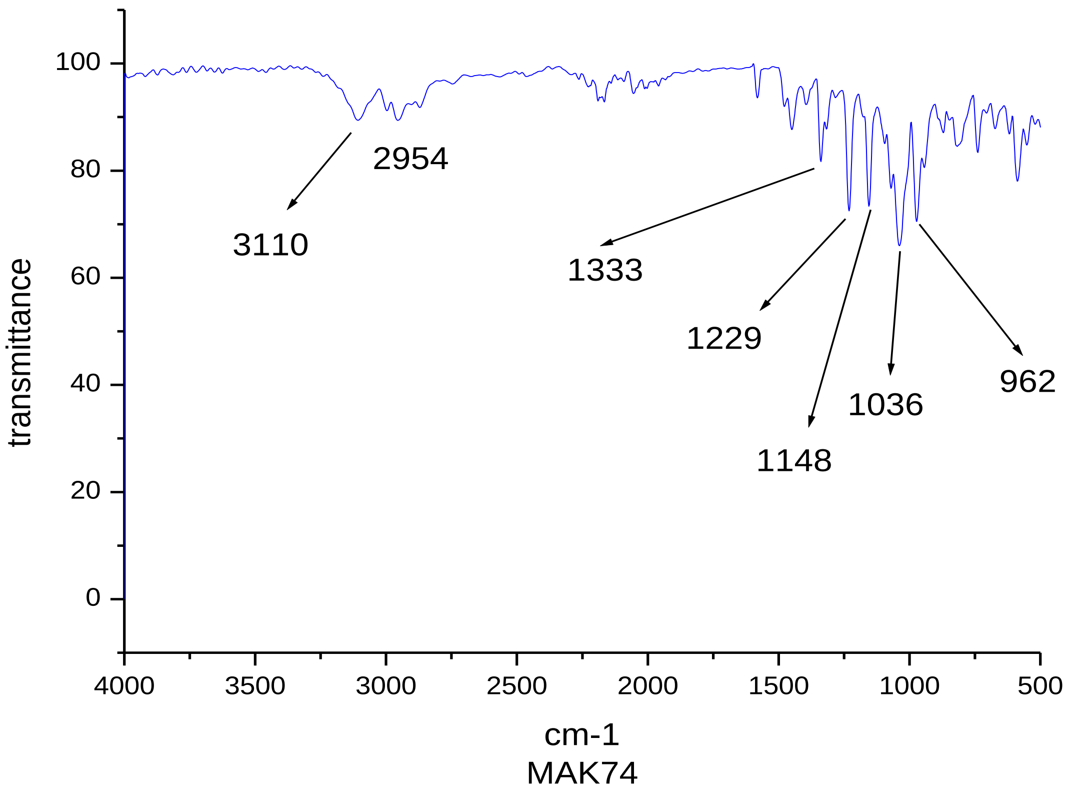

HRMS (ESI+):

Elemental Composition Report **Page 1**

Single Mass Analysis
Tolerance = 1.0 PPM / DBE: min = -10.0, max = 50.0
Element prediction: Off
Number of isotope peaks used for i-FIT = 3

Monoisotopic Mass, Even Electron Ions
982 formula(e) evaluated with 2 results within limits (up to 50 best isotopic matches for each mass)
Elements Used:
C: 0-100 H: 0-100 N: 0-20 O: 0-20 S: 1-1 Br: 1-1 P: 1-1

SYNAPT G2-S#UEB205 MAK74 17-May-2016
Y-JOM16051707 3 (0.141) Cm (3) 1: TOF MS ES+
1.30e+006

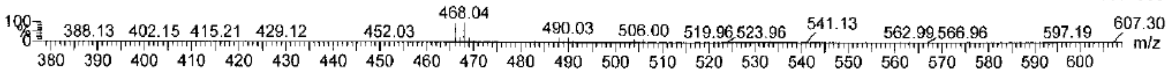

Minimum:				-10.0				
Maximum:		1.0	1.0	50.0				
Mass	Calc. Mass	mDa	PPM	DBE	i-FIT	Norm	Conf(%)	Formula
466.0455	466.0453	0.2	0.4	5.5	1810.1	0.000	100.00	C17 H26 N O5 S Br P
	466.0458	-0.3	-0.6	-1.5	1829.0	18.913	0.00	C2 H22 N13 O6 S Br P

Diethyl((6-(*N*,4-dimethylphenylsulfonamido)cyclohex-1-en-1-yl)methyl)phosphonate (6d)

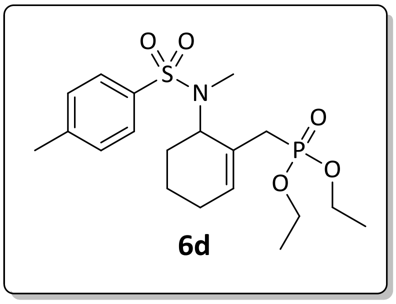

6d

^{1}H NMR (300 MHz, $CDCl_3$):

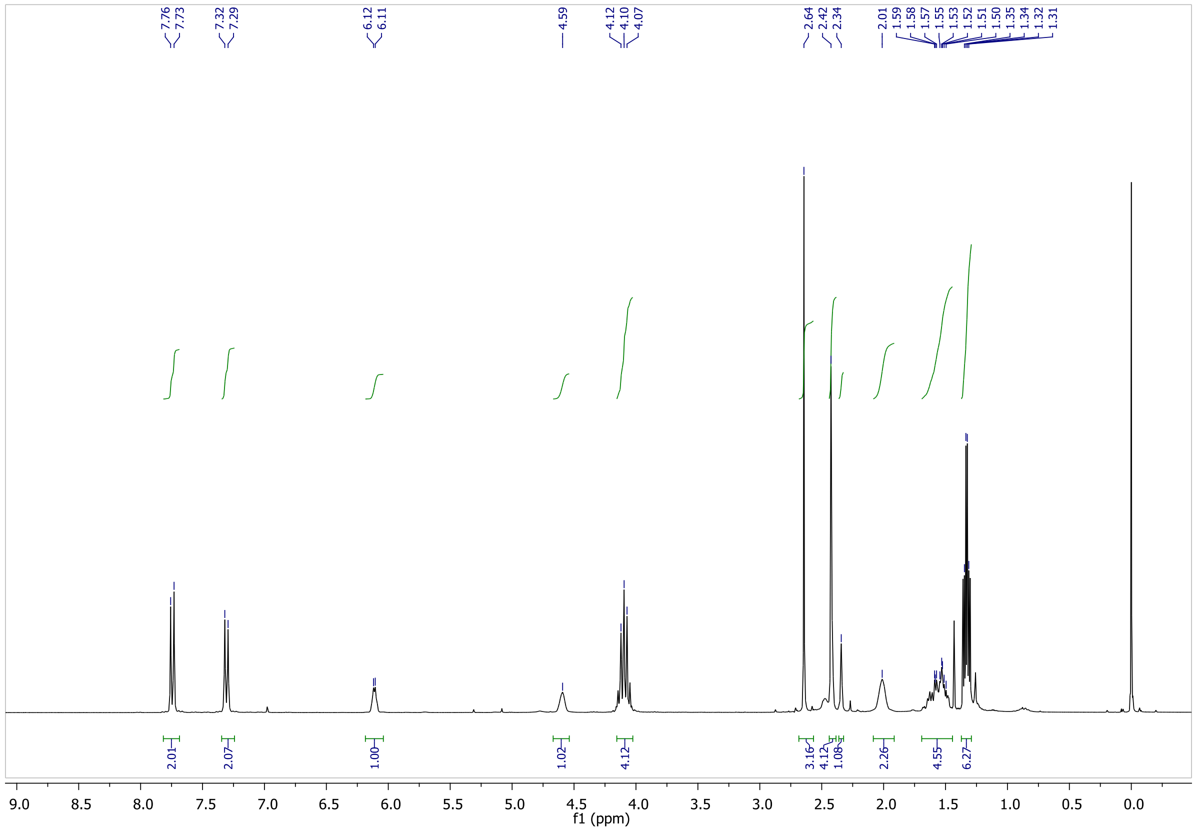

^{13}C NMR (75 MHz, $CDCl_3$):

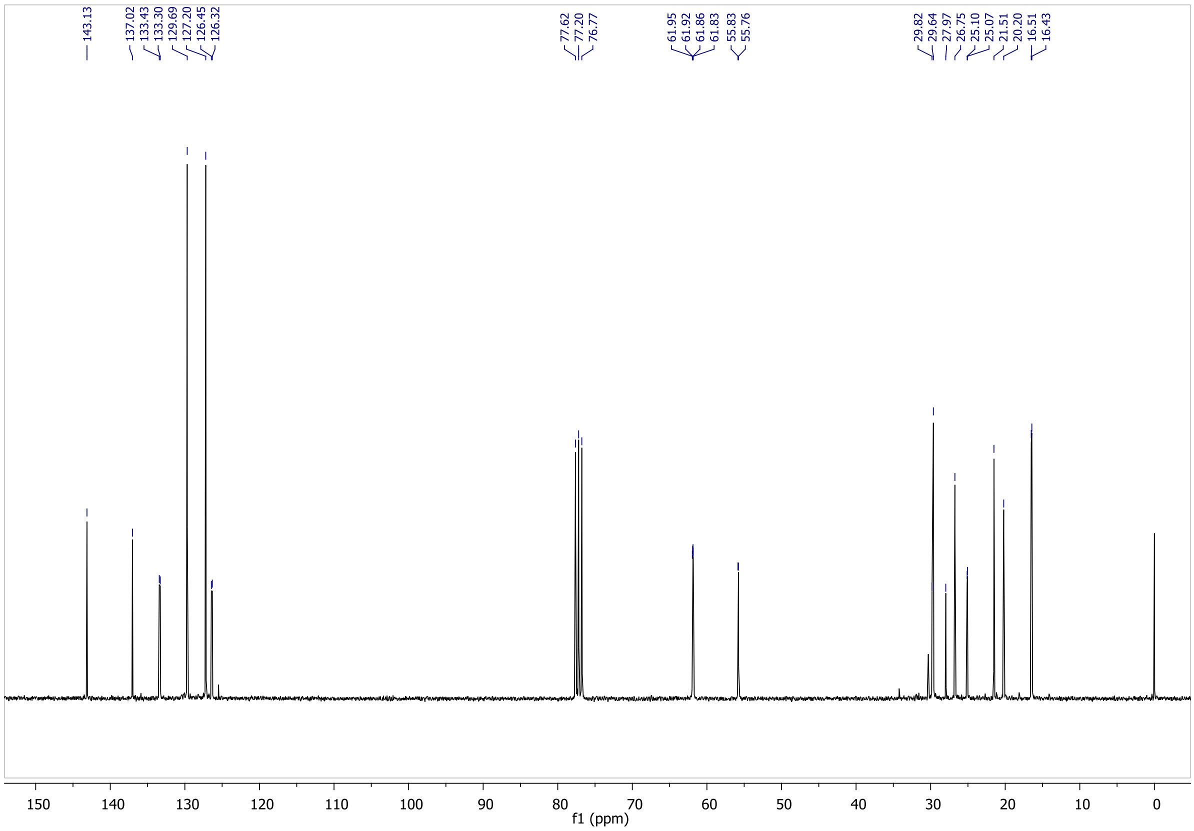

^{31}P NMR (121 MHz, $CDCl_3$):

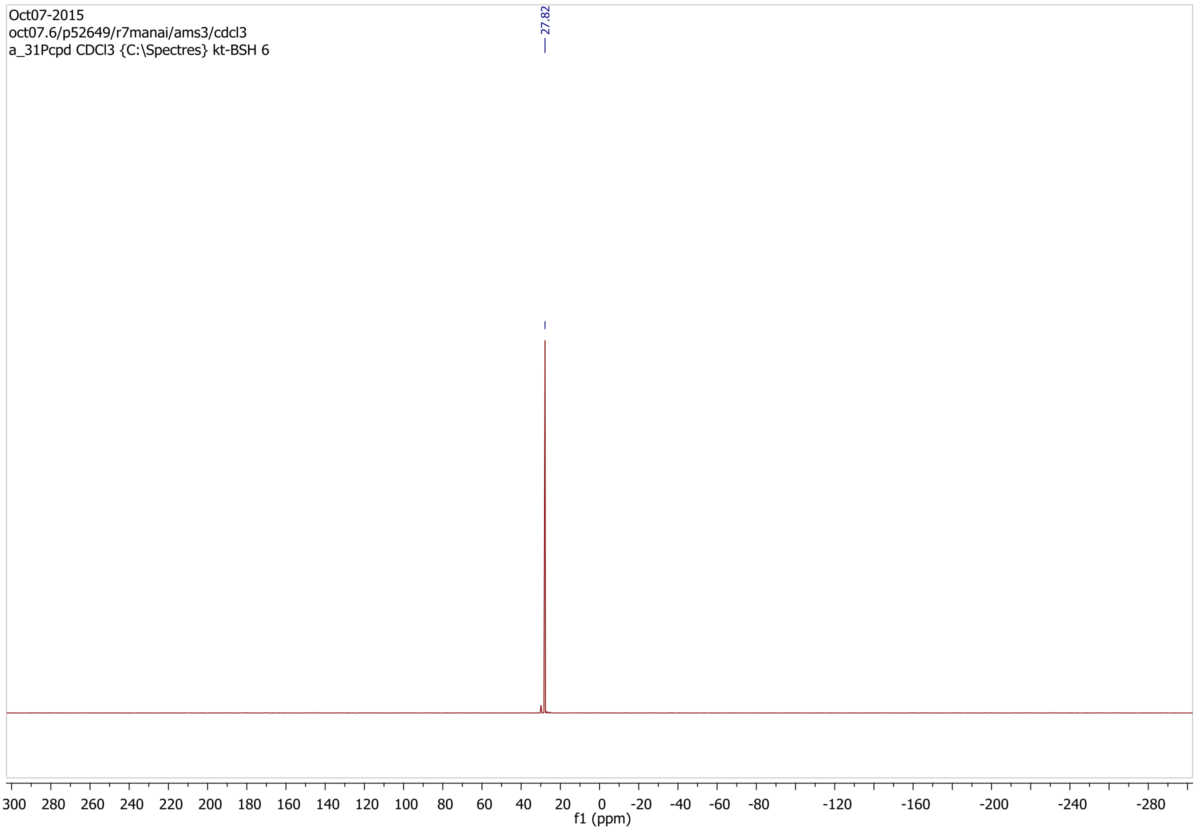

IR (FT-IR):

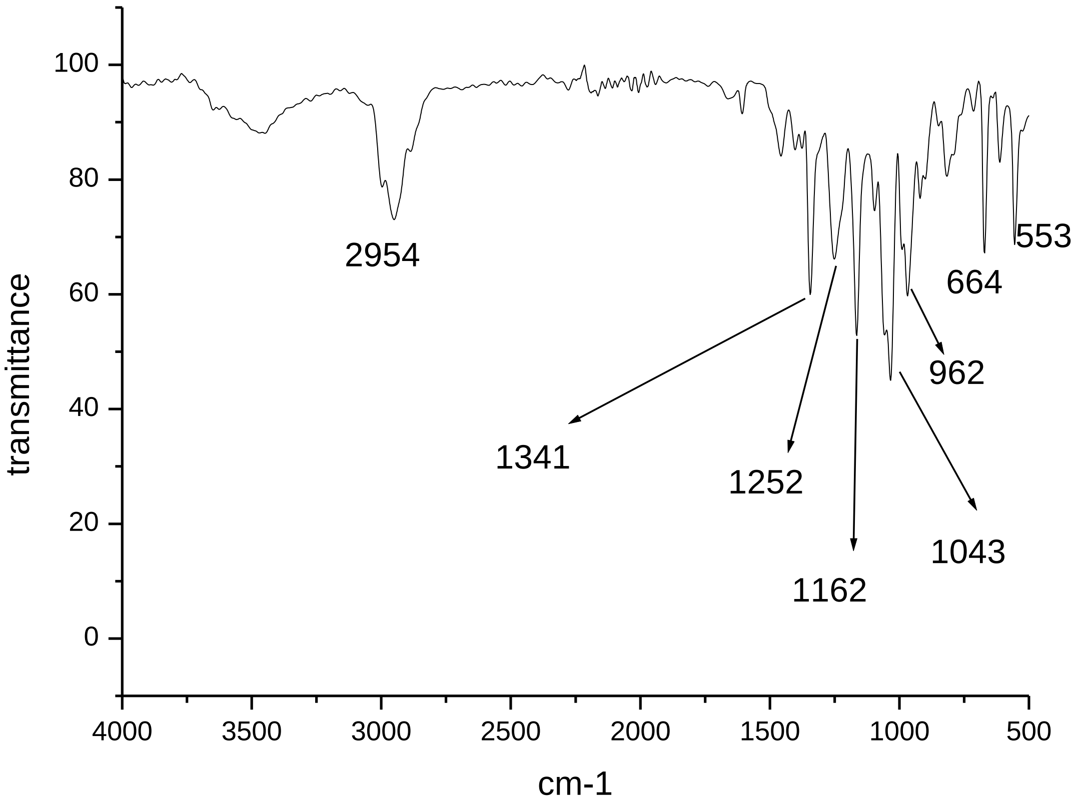

MAK73

HRMS (ESI+):

Elemental Composition Report **Page 1**

Single Mass Analysis
Tolerance = 1.0 PPM / DBE: min = -10.0, max = 50.0
Element prediction: Off
Number of isotope peaks used for i-FIT = 3

Monoisotopic Mass, Even Electron Ions
1220 formula(e) evaluated with 1 results within limits (up to 50 best isotopic matches for each mass)
Elements Used:
C: 0-100 H: 0-100 N: 0-20 O: 0-20 S: 1-1 P: 1-1

SYNAPT G2-S#UEB205 MAK73 17-May-2016
Y-JOM16051702 3 (0.141) Cm (3) 1: TOF MS ES+
9.99e+005

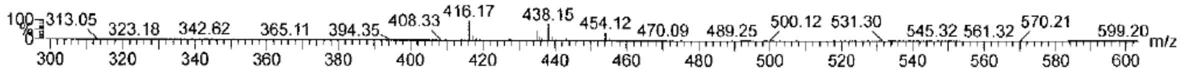

Mass	Calc. Mass	mDa	PPM	DBE	i-FIT	Norm	Conf(%)	Formula
Minimum:				-10.0				
Maximum:		1.0	1.0	50.0				
416.1661	416.1661	0.0	0.0	5.5	1735.6	n/a	n/a	C19 H31 N O5 S P

2-((Diethoxyphosphoryl)methyl)cyclohex-2-en-1-yl acetate (7)

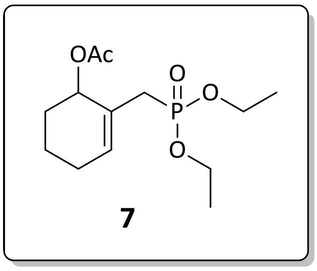

^{1}H NMR (300 MHz, $CDCl_3$):

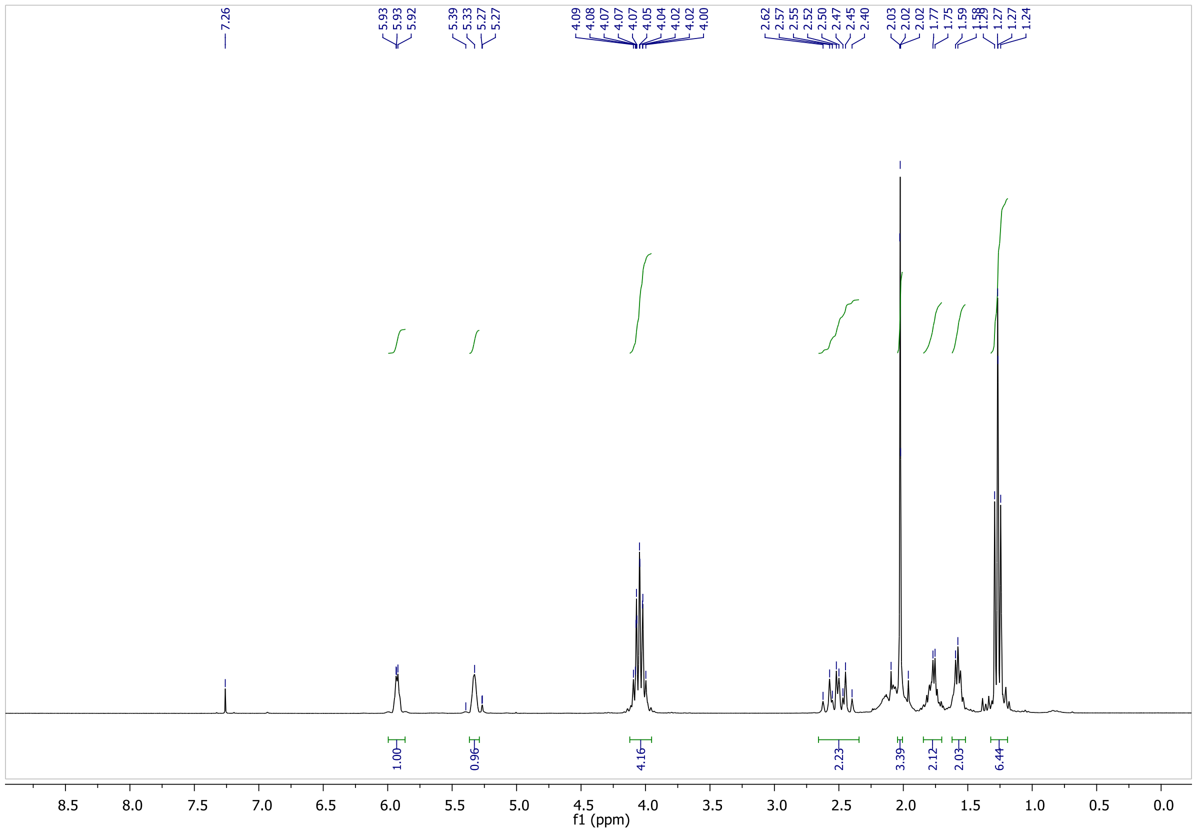

^{13}C NMR (75 MHz, $CDCl_3$):

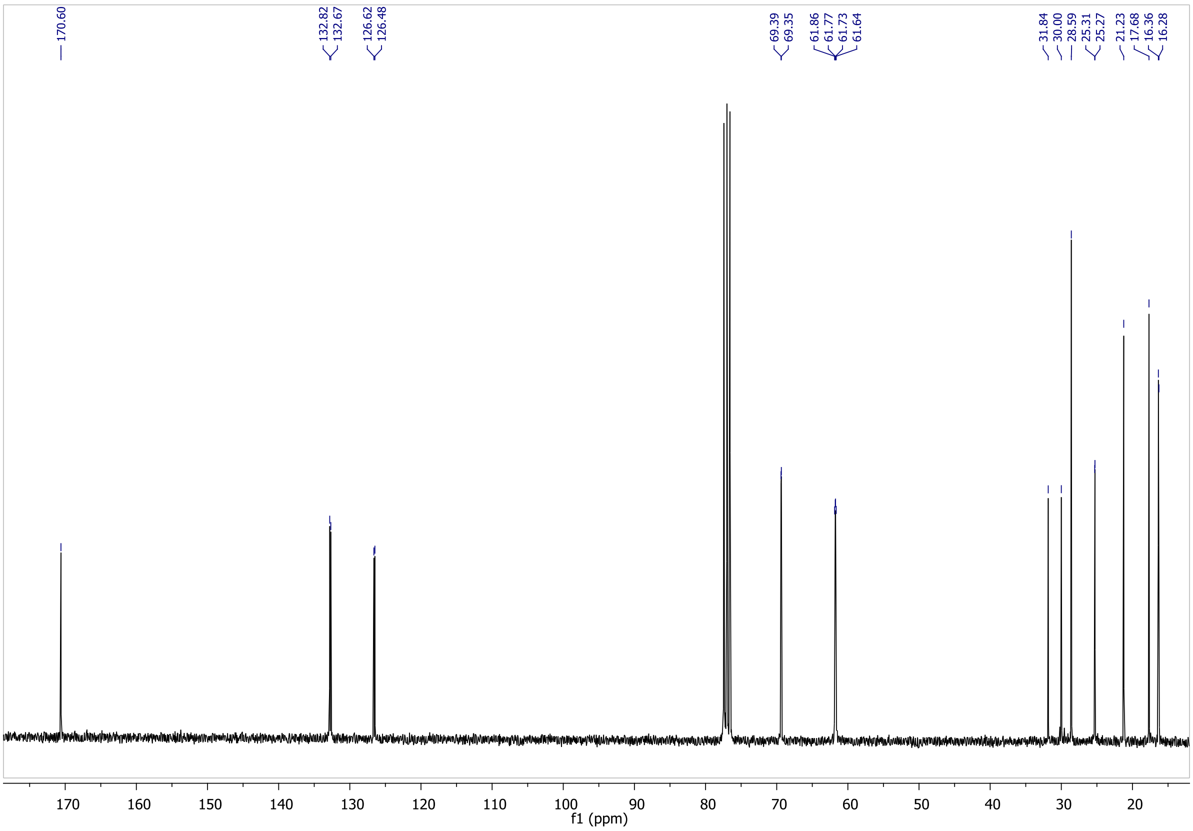

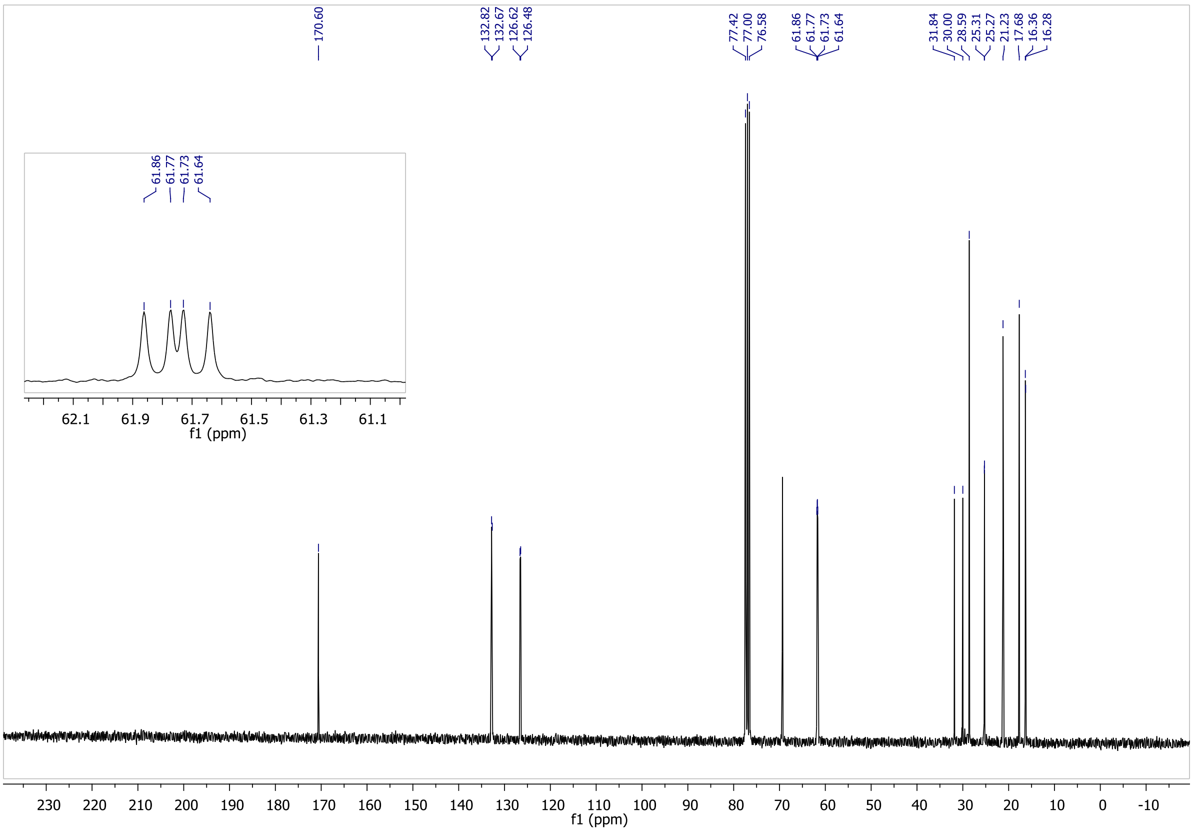

^{31}P NMR (121 MHz, $CDCl_3$):

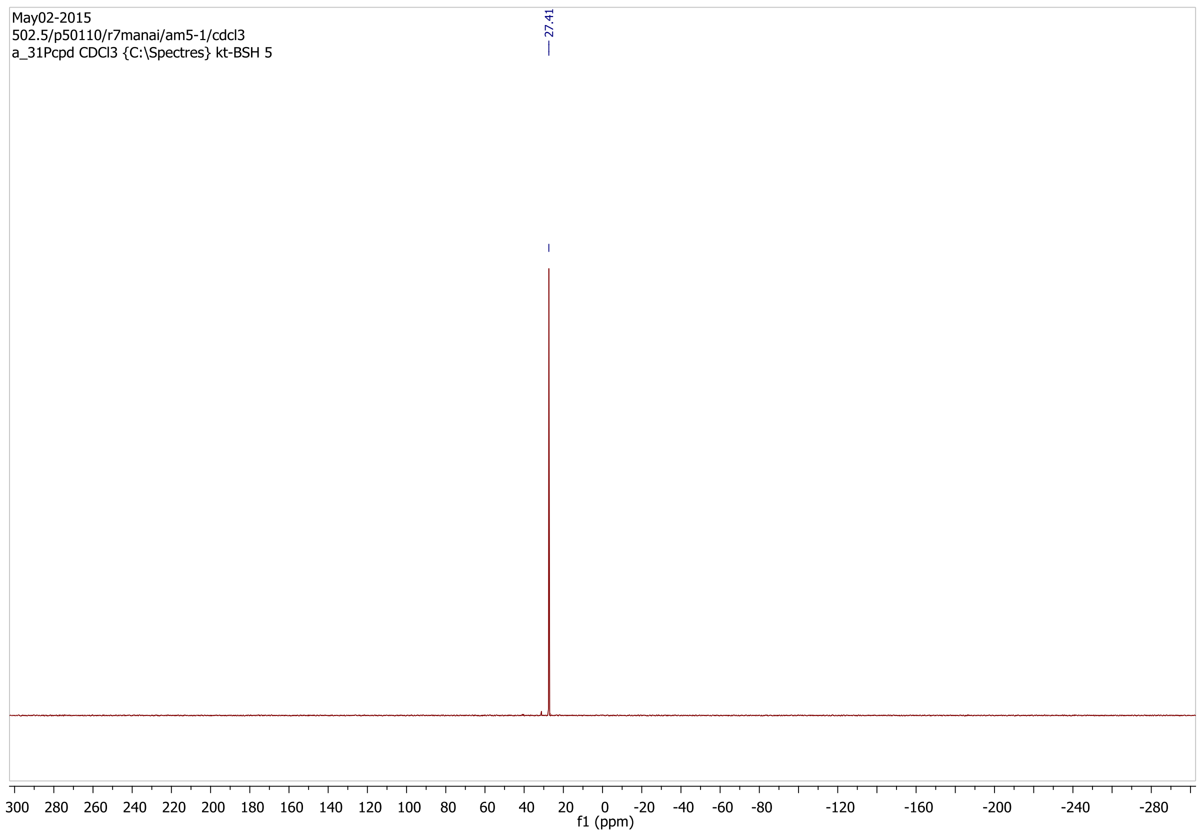

IR (FT-IR):

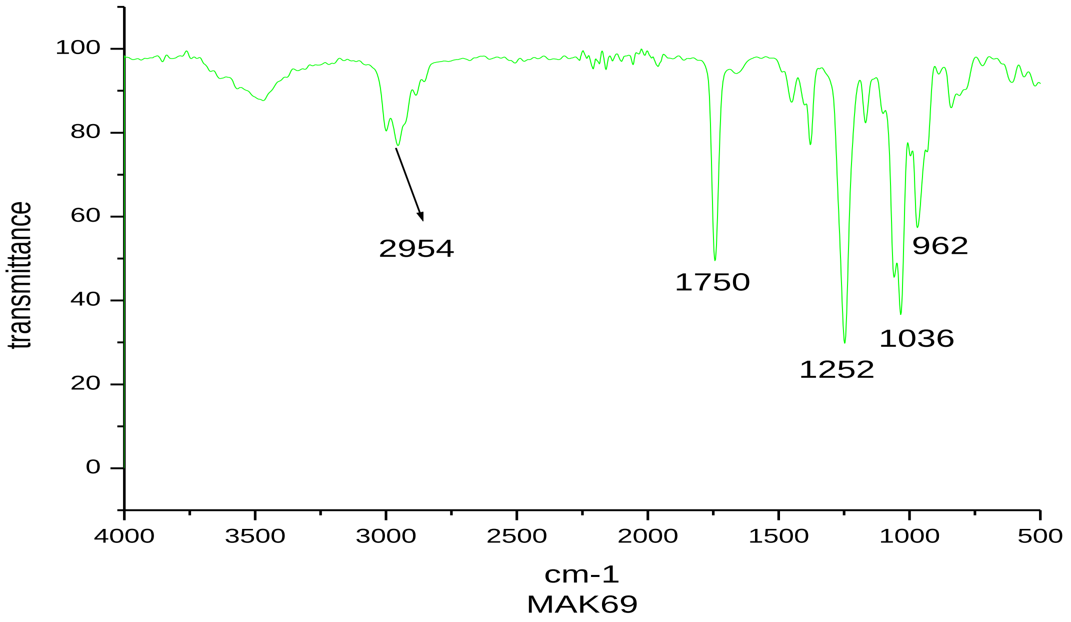

HRMS (ESI+):

Elemental Composition Report **Page 1**

Single Mass Analysis
Tolerance = 1.0 PPM / DBE: min = -10.0, max = 50.0
Element prediction: Off
Number of isotope peaks used for i-FIT = 3

Monoisotopic Mass, Even Electron Ions
1317 formula(e) evaluated with 2 results within limits (up to 50 best isotopic matches for each mass)
Elements Used:
C: 0-100 H: 0-100 N: 0-20 O: 0-20 P: 1-1 Na: 0-1

SYNAPT G2-S#UEB205 MAK69 17-May-2016
Y-JOM16051704 10 (0.438) Cm (10) 1: TOF MS ES+
5.31e+005

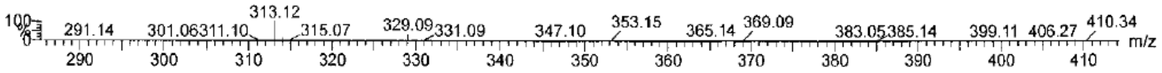

Minimum:				-10.0				
Maximum:		1.0	1.0	50.0				
Mass	Calc. Mass	mDa	PPM	DBE	i-FIT	Norm	Conf(%)	Formula
313.1179	313.1181	-0.2	-0.6	2.5	1859.2	0.038	96.31	C13 H23 O5 P Na
	313.1178	0.1	0.3	6.5	1862.4	3.300	3.69	C11 H18 N6 O3 P

Diethyl (6-(phenylamino)cyclohex-1-en-1-yl)methylphosphonate (8a)

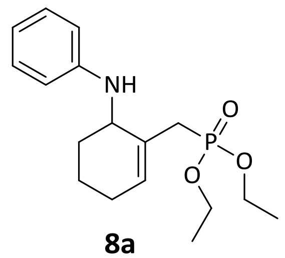

8a

^{1}H NMR (300 MHz, $CDCl_3$):

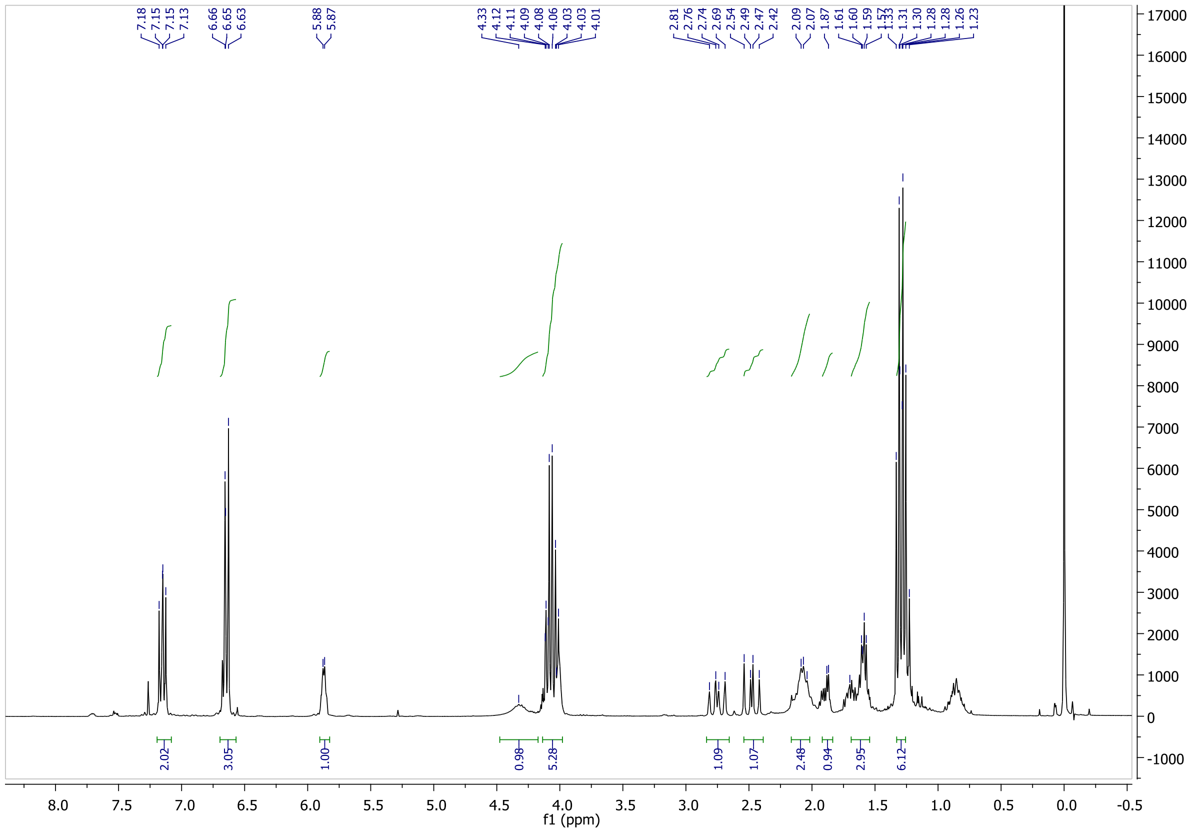

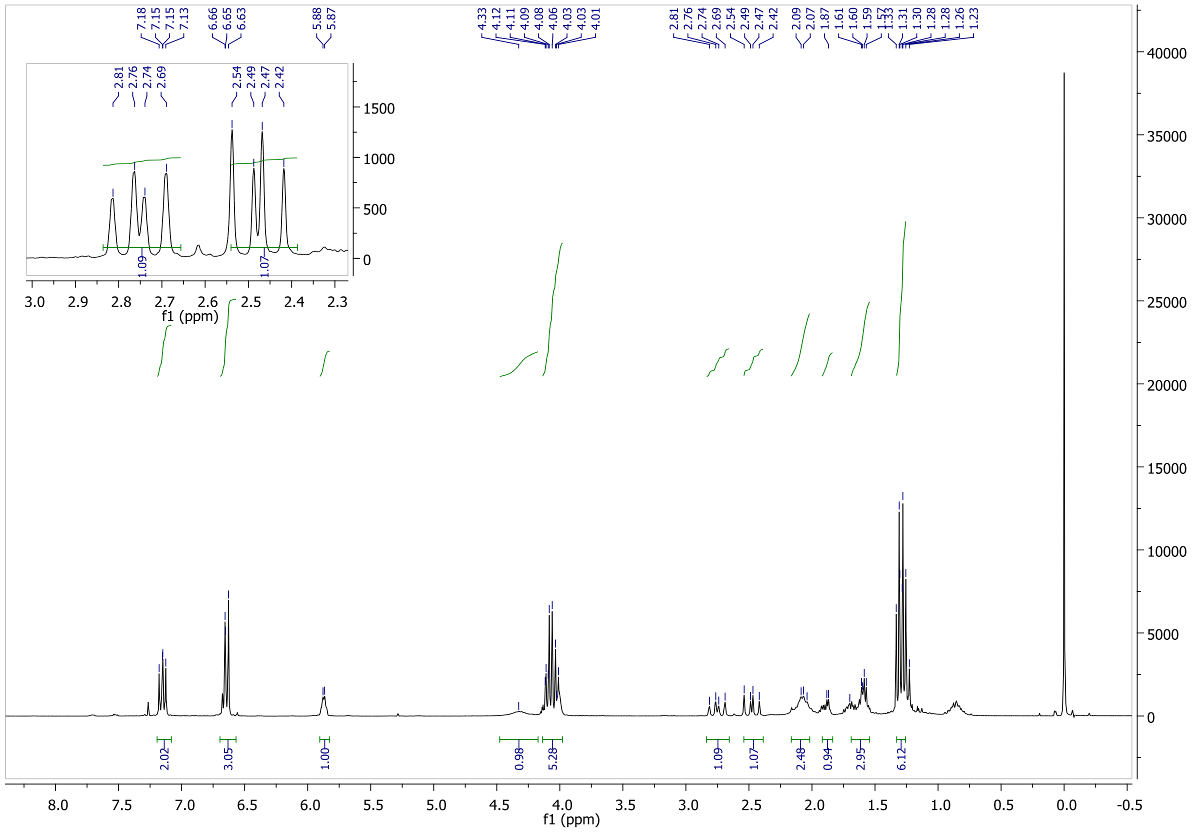

^{13}C NMR (75 MHz, $CDCl_3$):

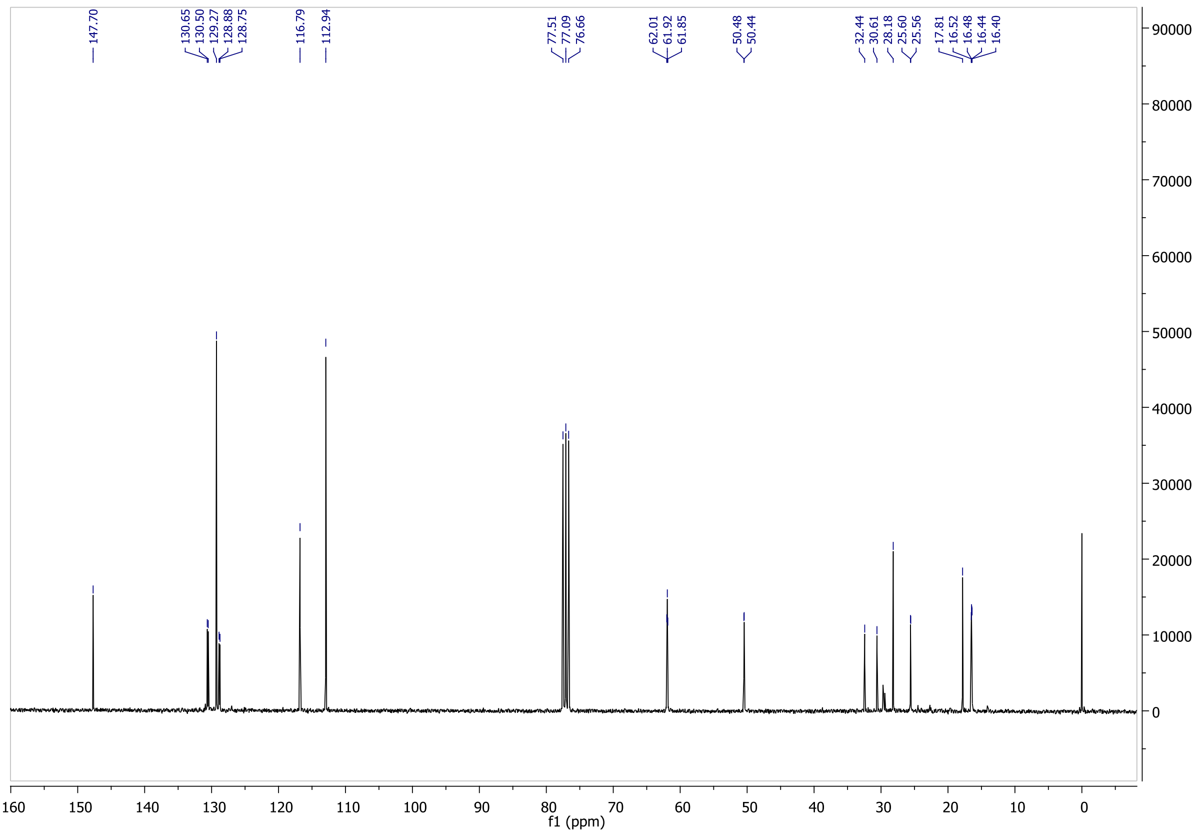

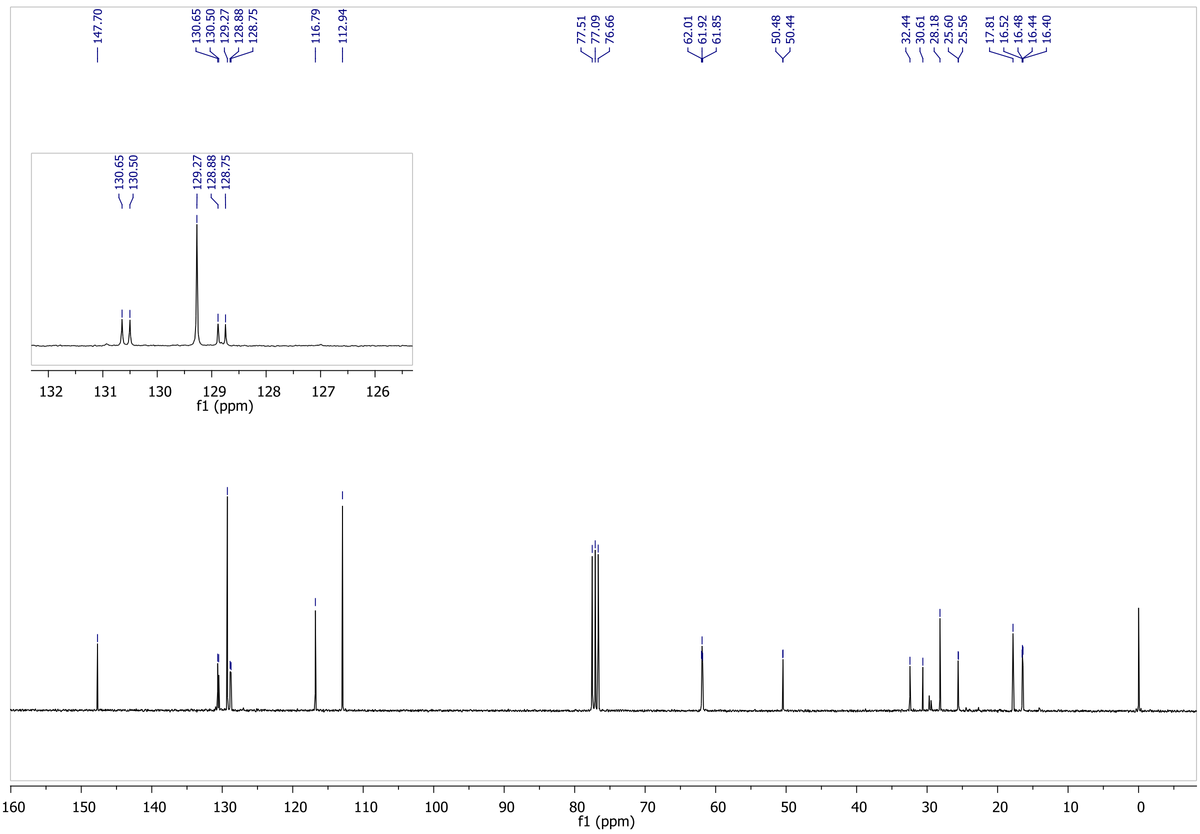

^{31}P NMR (121 MHz, $CDCl_3$):

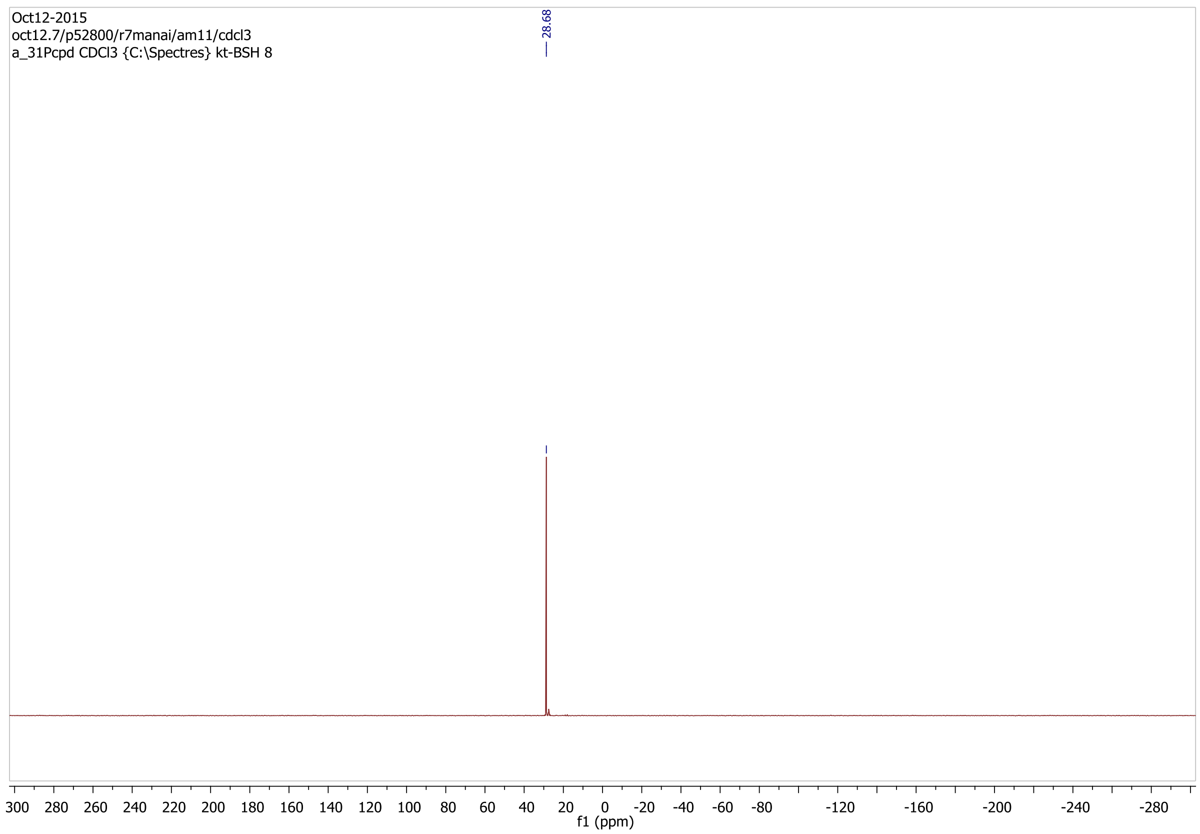

IR (FT-IR):

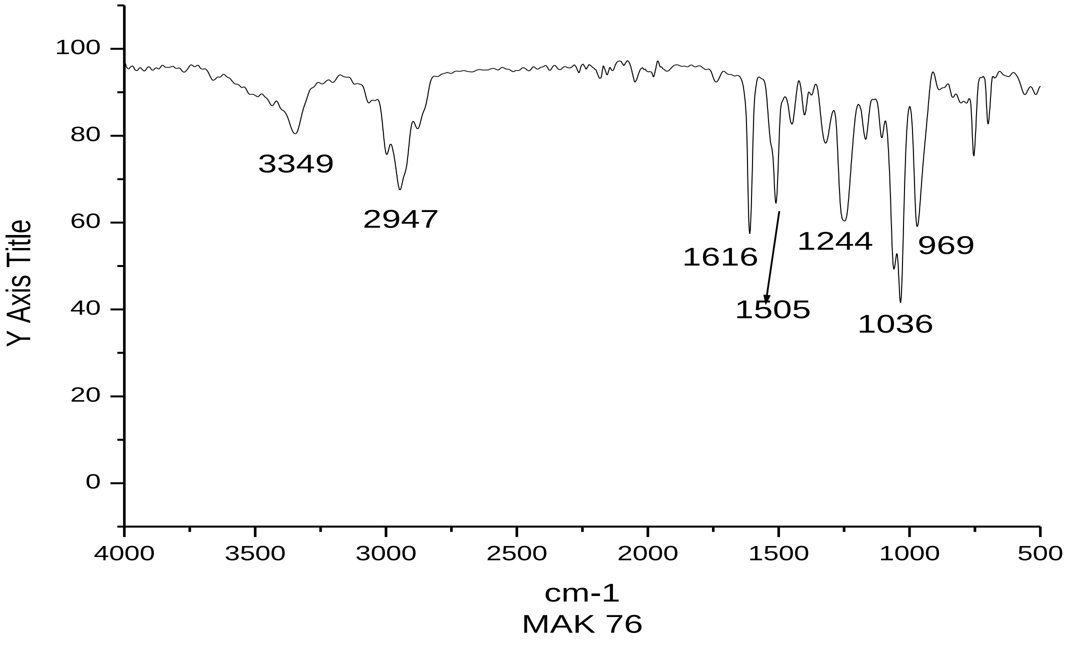

HRMS (ESI+):

Elemental Composition Report **Page 1**

Single Mass Analysis
Tolerance = 1.0 mDa / DBE: min = -50.0, max = 100.0
Element prediction: Off
Number of isotope peaks used for i-FIT = 3

Monoisotopic Mass, Even Electron Ions
3713 formula(e) evaluated with 3 results within limits (all results (up to 1000) for each mass)
Elements Used:
C: 0-100 H: 0-100 N: 0-10 O: 0-10 S: 0-1 P: 0-1

SYNAPT G2-S#UEB205 MAK76 19-May-2016
Y-JOM16051903 3 (0.141) Cm (3) 1: TOF MS ES+
8.91e+006

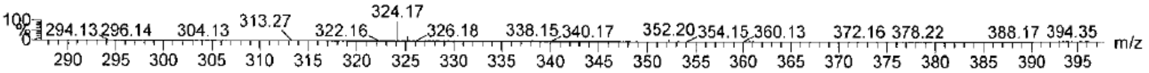

Minimum:				-50.0				
Maximum:		1.0	1.0	100.0				
Mass	Calc. Mass	mDa	PPM	DBE	i-FIT	Norm	Conf(%)	Formula
324.1733	324.1729	0.4	1.2	5.5	1704.8	0.000	100.00	C17 H27 N O3 P
	324.1731	0.2	0.6	-2.5	1716.5	11.726	0.00	C7 H26 N5 O9
	324.1735	-0.2	-0.6	1.5	1724.6	19.843	0.00	C10 H27 N7 O S P

Diethyl (6-((4-nitrophenyl)amino)cyclohex-1-en-1-yl)methylphosphonate (8b)

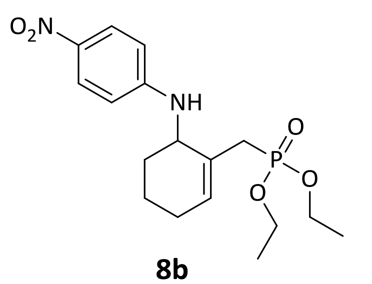

8b

^{1}H NMR (300 MHz, $CDCl_3$):

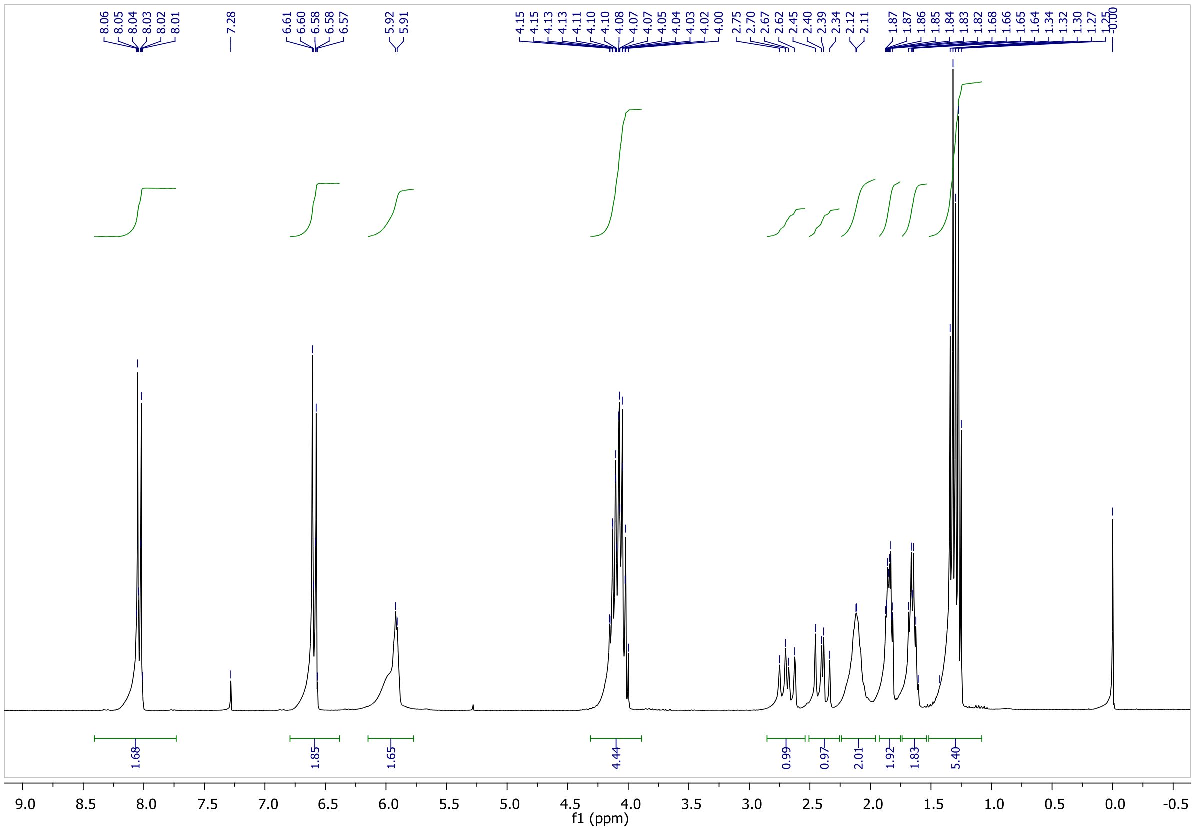

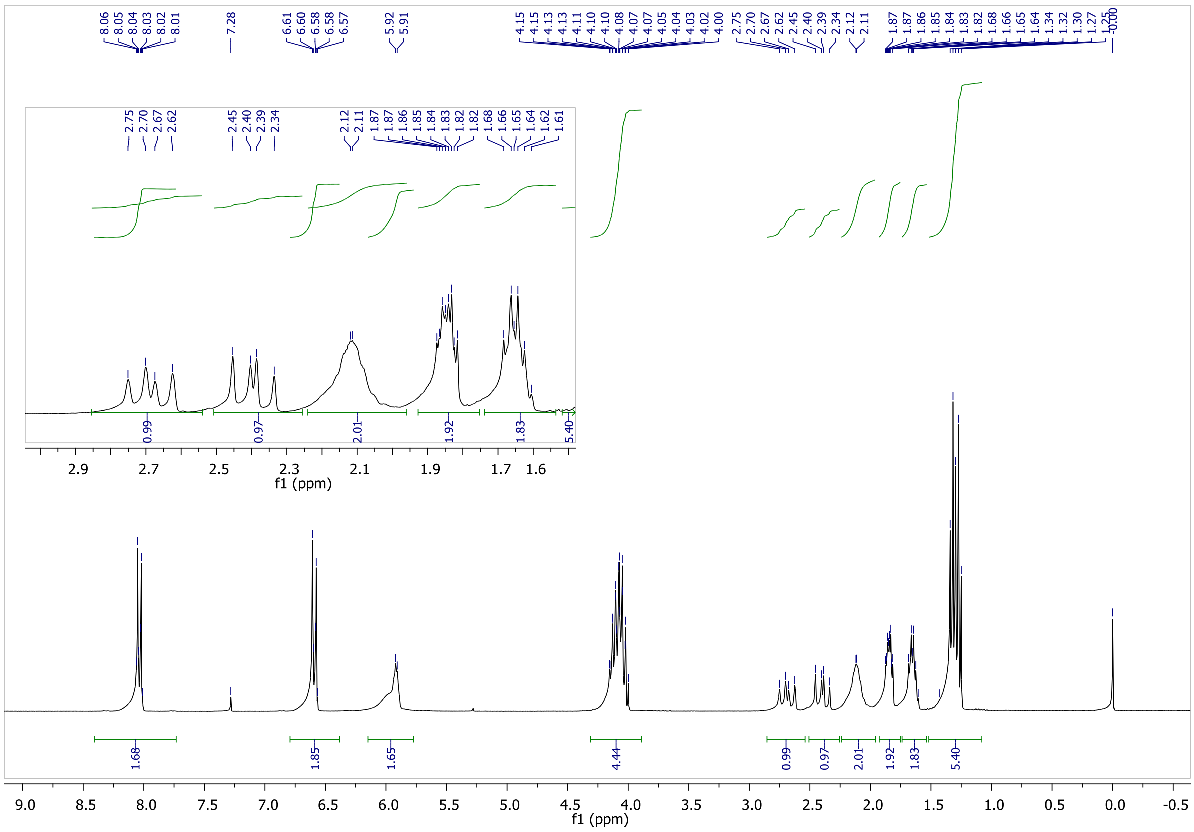

^{13}C NMR (75 MHz, $CDCl_3$):

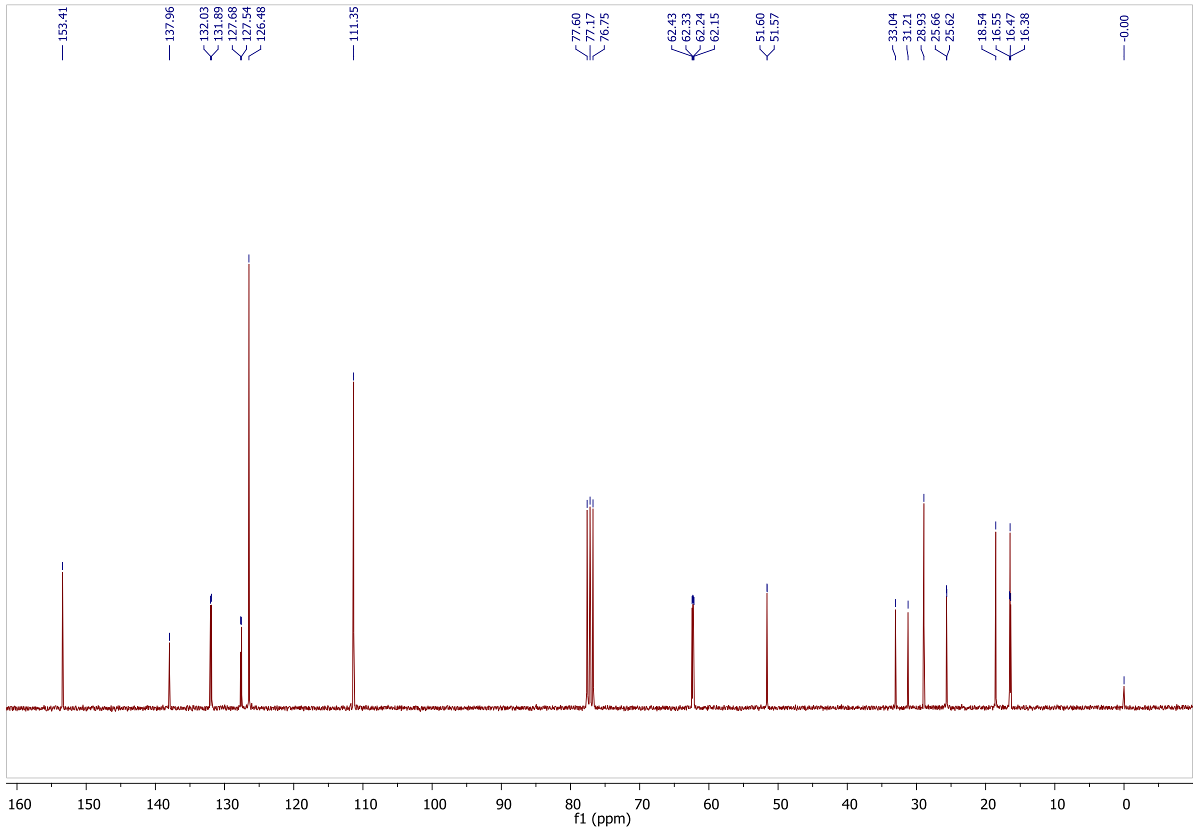

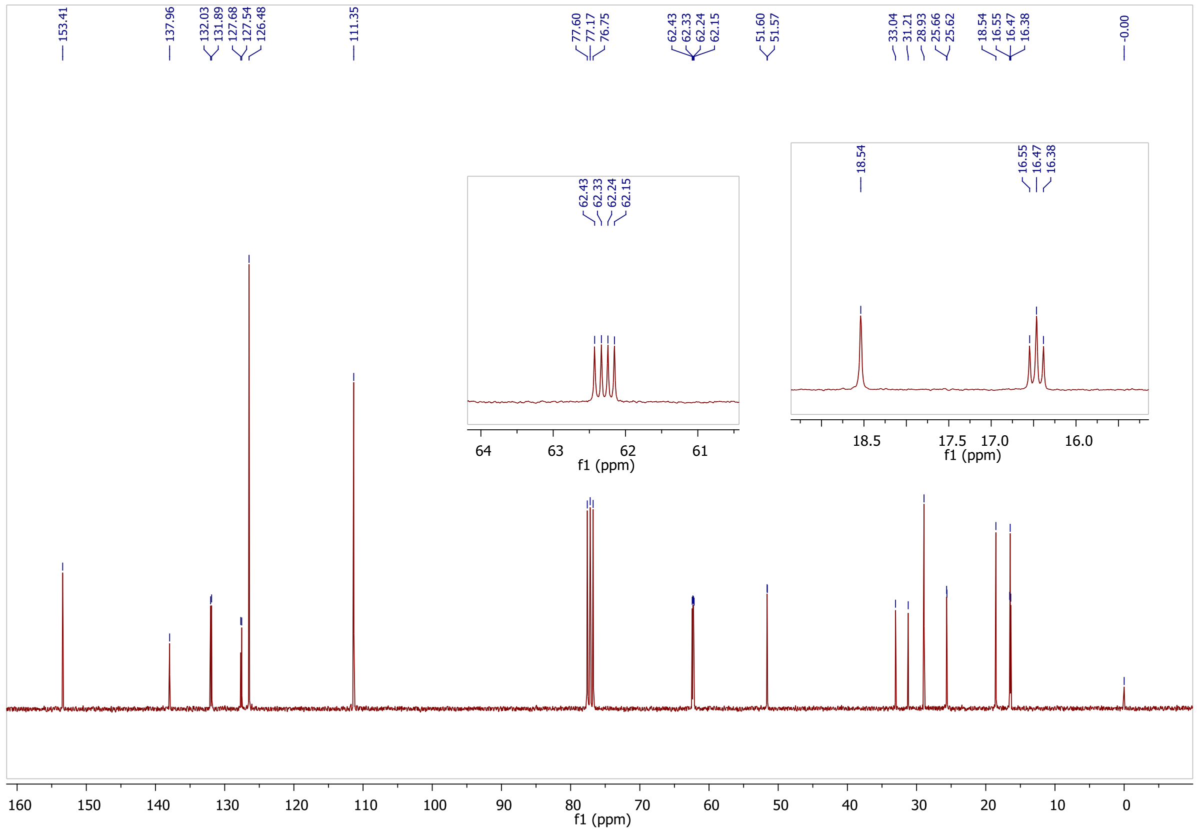

^{31}P NMR (121 MHz, $CDCl_3$):

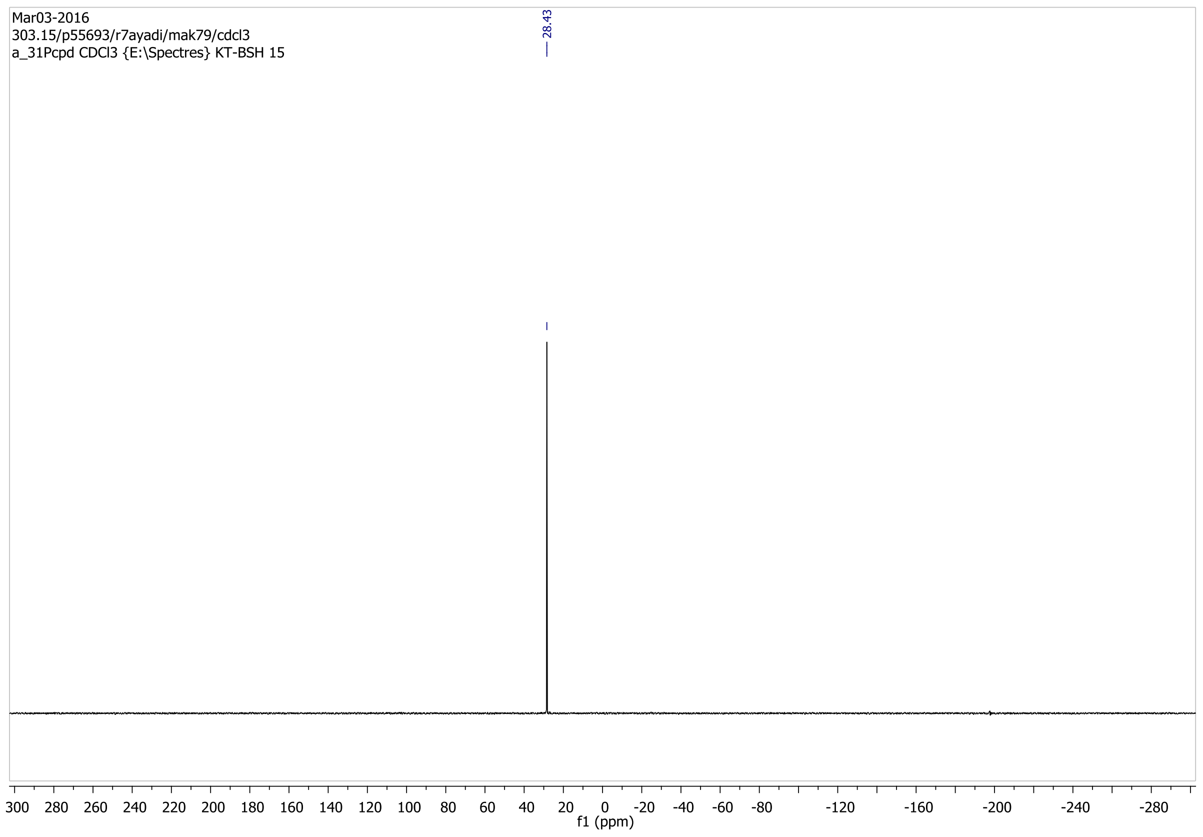

IR (FT-IR):

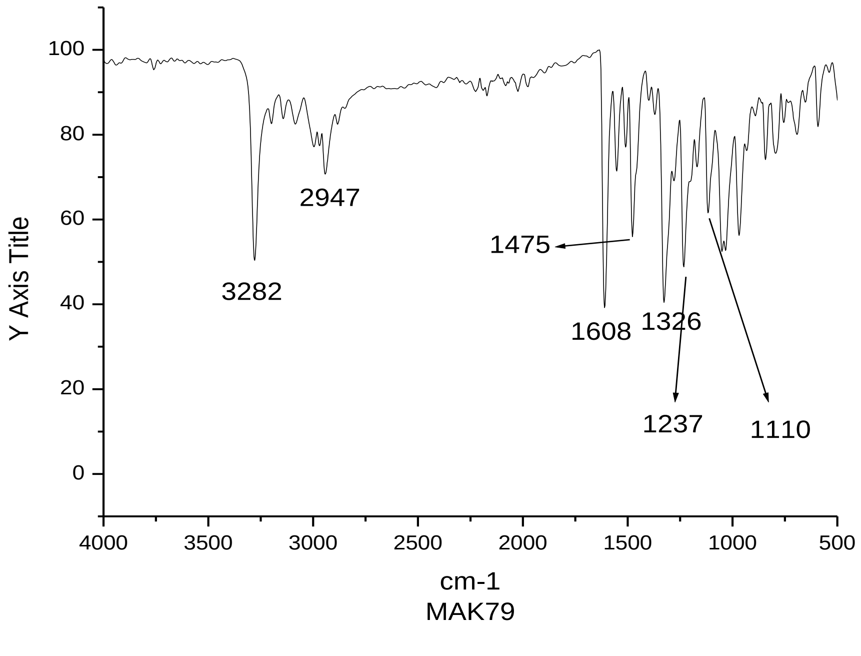

HRMS (ESI+):

Elemental Composition Report **Page 1**

Single Mass Analysis
Tolerance = 1.0 PPM / DBE: min = -10.0, max = 50.0
Element prediction: Off
Number of isotope peaks used for i-FIT = 3

Monoisotopic Mass, Even Electron Ions
2027 formula(e) evaluated with 2 results within limits (up to 50 best isotopic matches for each mass)
Elements Used:
C: 0-100 H: 0-100 N: 0-20 O: 0-20 P: 1-1 Na: 0-1

SYNAPT G2-S#UEB205 MAK79 17-May-2016
Y-JOM16051706 3 (0.141) Cm (3:4) 1: TOF MS ES+
3.03e+006

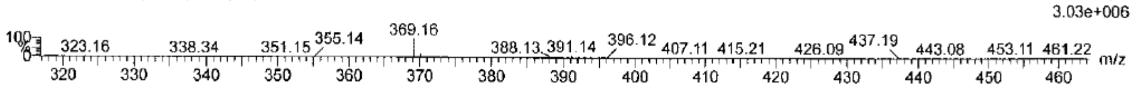

Minimum:				-10.0				
Maximum:		1.0	1.0	50.0				
Mass	Calc. Mass	mDa	PPM	DBE	i-FIT	Norm	Conf(%)	Formula
369.1582	369.1579	0.3	0.8	6.5	2564.3	0.000	100.00	C17 H26 N2 O5 P
	369.1584	-0.2	-0.5	-0.5	2588.2	23.879	0.00	C2 H22 N14 O6 P

Diethyl (6-((4-fluorophenyl)amino)cyclohex-1-en-1-yl)methylphosphonate (8c)

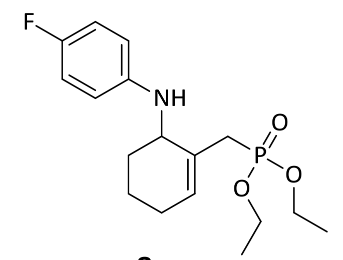

8c

^{1}H NMR (300 MHz, $CDCl_3$) :

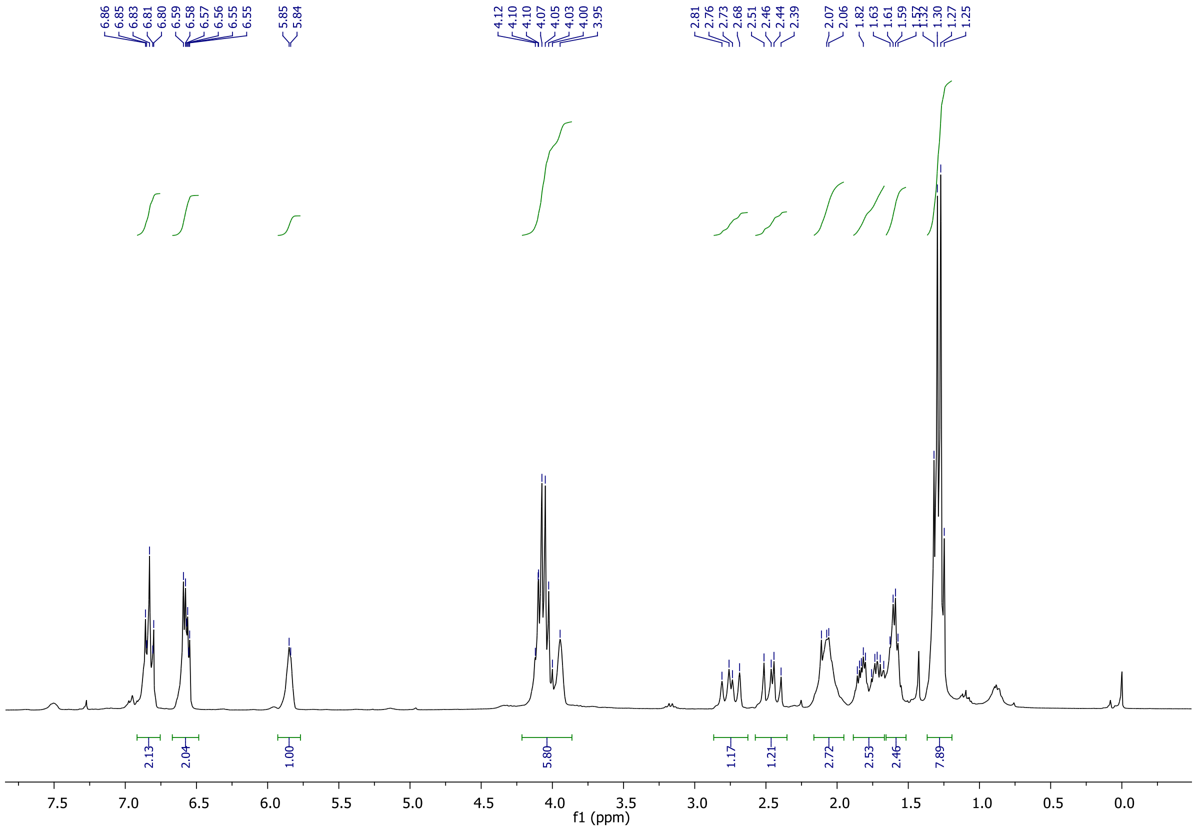

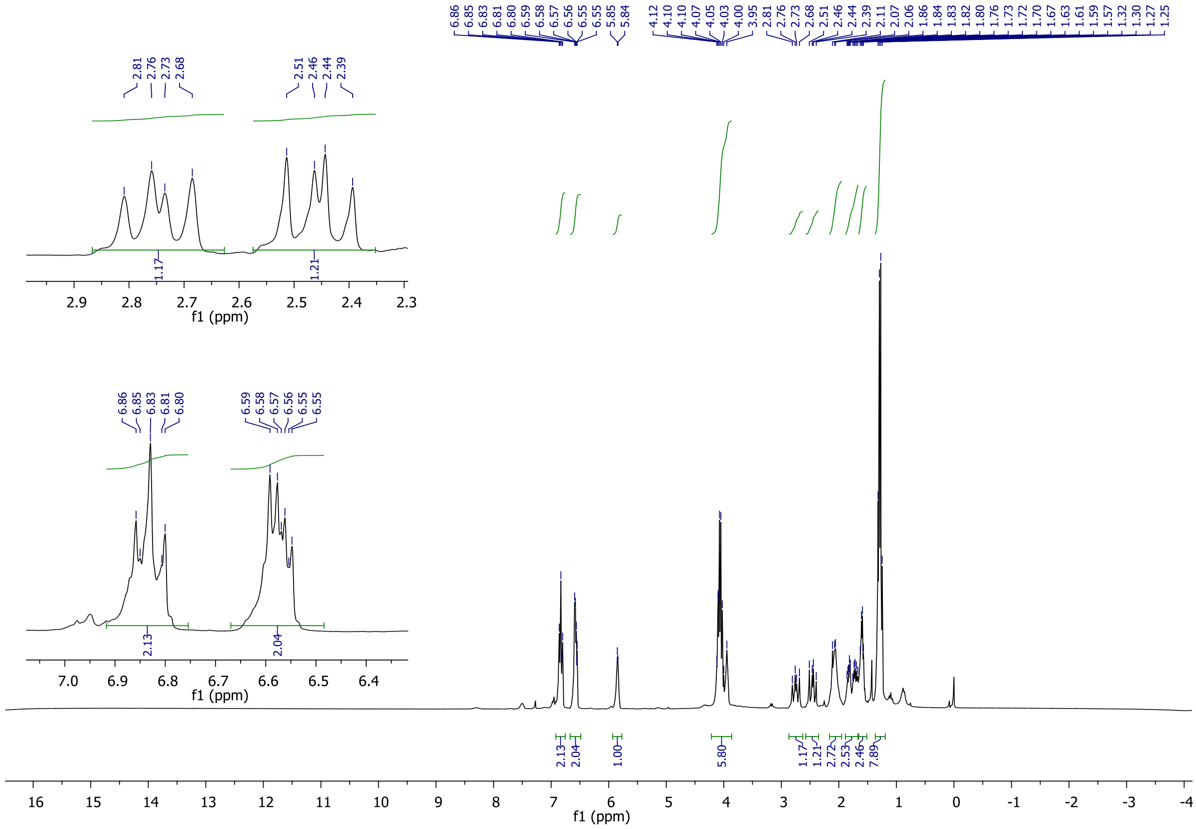

^{13}C NMR (75MHz , $CDCl_3$) :

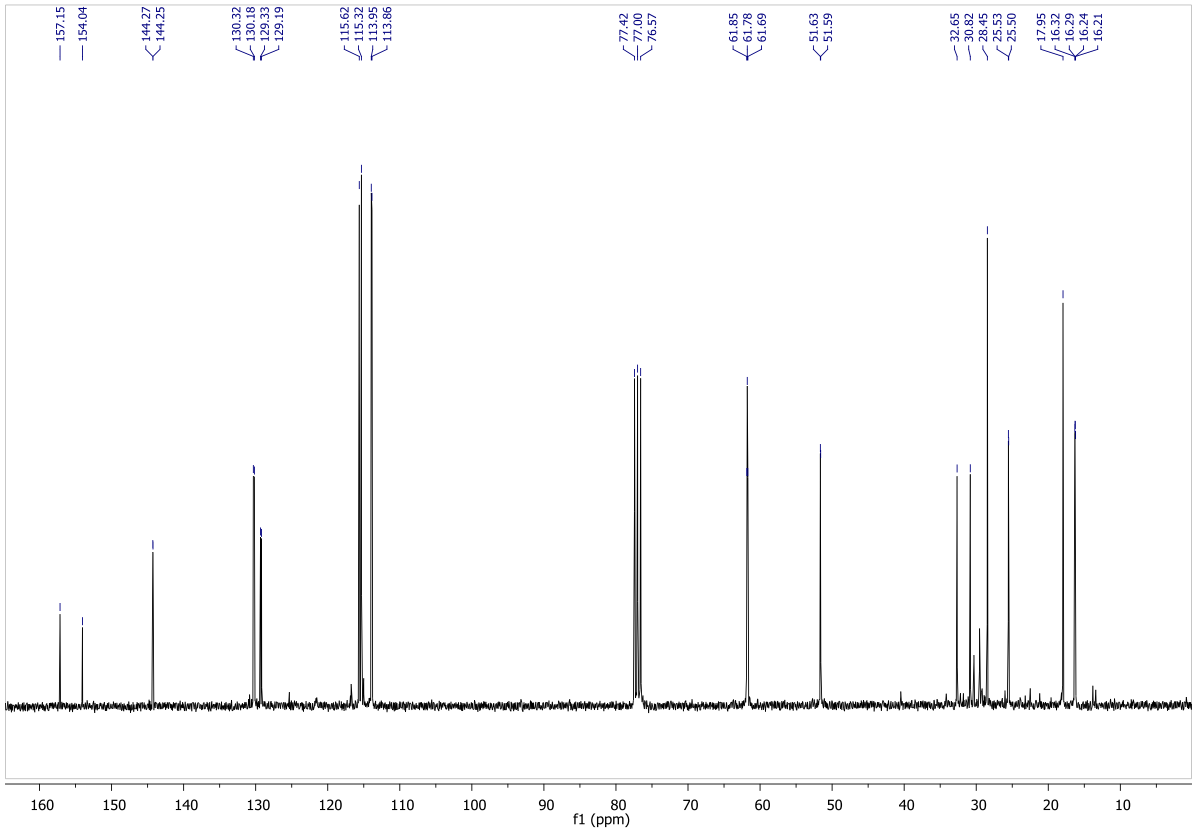

^{19}F NMR (300 MHz, $CDCl_3$):

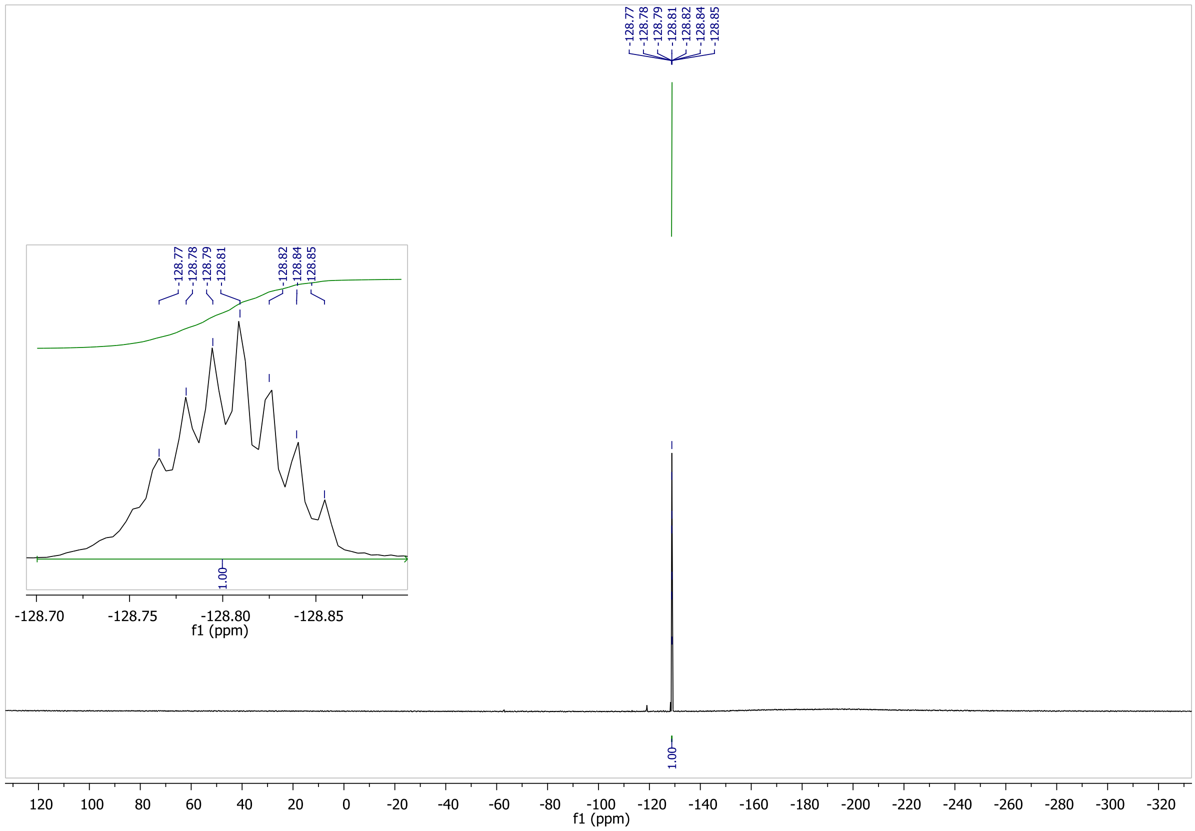

^{31}P NMR (121 MHz, $CDCl_3$):

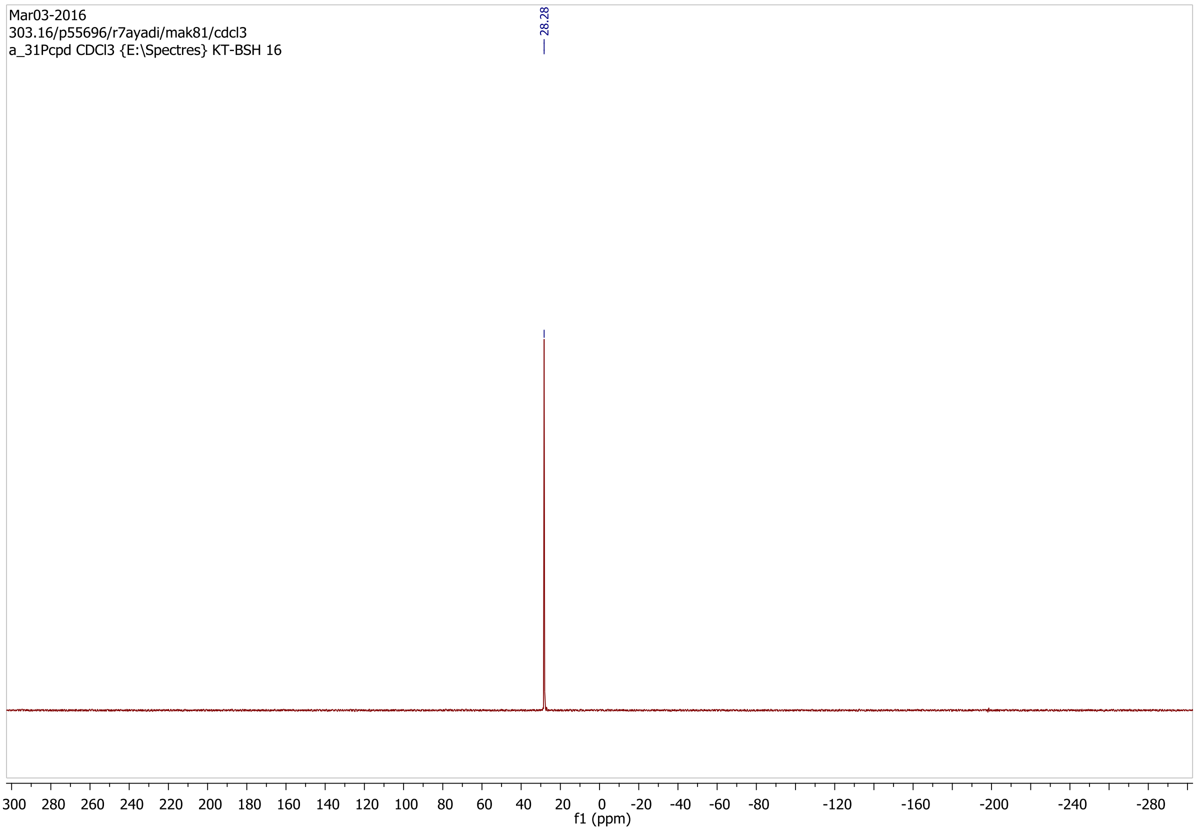

IR (FT-IR):

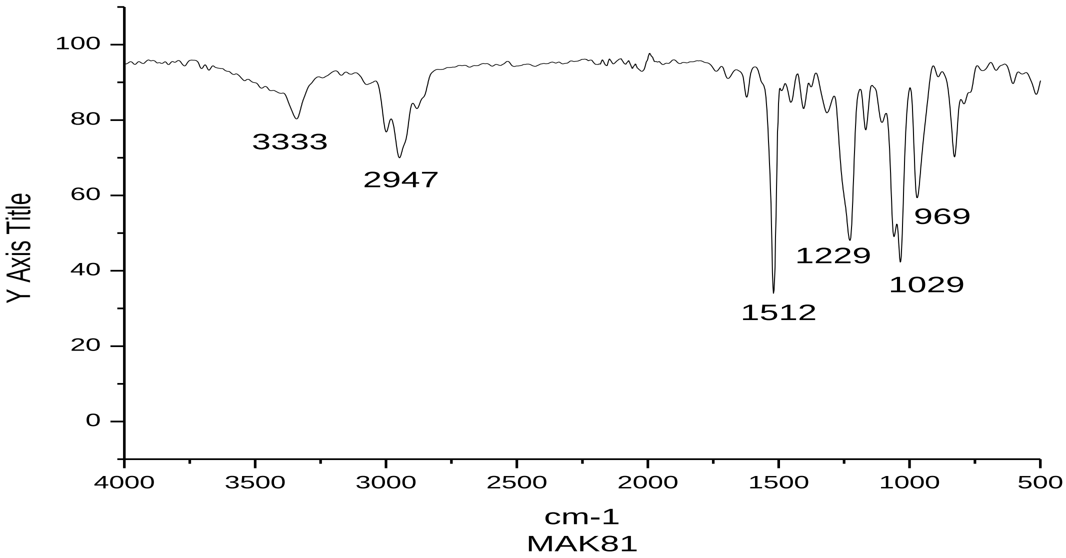

HRMS (ESI+):

Elemental Composition Report **Page 1**

Single Mass Analysis
Tolerance = 1.0 mDa / DBE: min = -50.0, max = 100.0
Element prediction: Off
Number of isotope peaks used for i-FIT = 3

Monoisotopic Mass, Even Electron Ions
3933 formula(e) evaluated with 4 results within limits (all results (up to 1000) for each mass)
Elements Used:
C: 0-100 H: 0-100 N: 0-10 O: 0-10 P: 0-1 F: 0-1

SYNAPT G2-S#UEB205 MAK81 19-May-2016
Y-JOM16051904 10 (0.437) Cm (10) 1: TOF MS ES+
3.42e+005

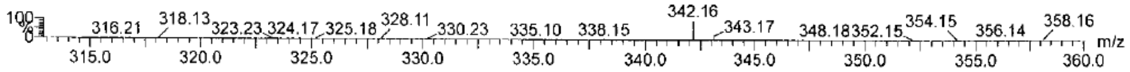

		mDa	PPM	DBE				
Minimum:				-50.0				
Maximum:		1.0	1.0	100.0				

Mass	Calc. Mass	mDa	PPM	DBE	i-FIT	Norm	Conf(%)	Formula
342.1636	342.1634	0.2	0.6	5.5	1628.2	0.000	100.00	C17 H26 N O3 P F
	342.1638	-0.2	-0.6	6.5	1638.7	10.507	0.00	C11 H20 N9 O4
	342.1641	-0.5	-1.5	-3.5	1640.4	12.199	0.00	C8 H29 N3 O9 P
	342.1636	0.0	0.0	-2.5	1641.7	13.503	0.00	C7 H25 N5 O9 F

Diethyl (6-(naphthalen-2-ylamino)cyclohex-1-en-1-yl)methylphosphonate (8d)

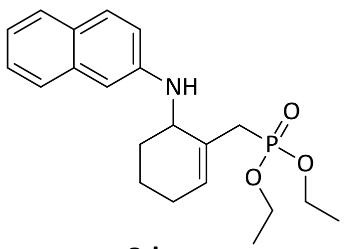

8d

^{1}H NMR (300 MHz, $CDCl_3$) :

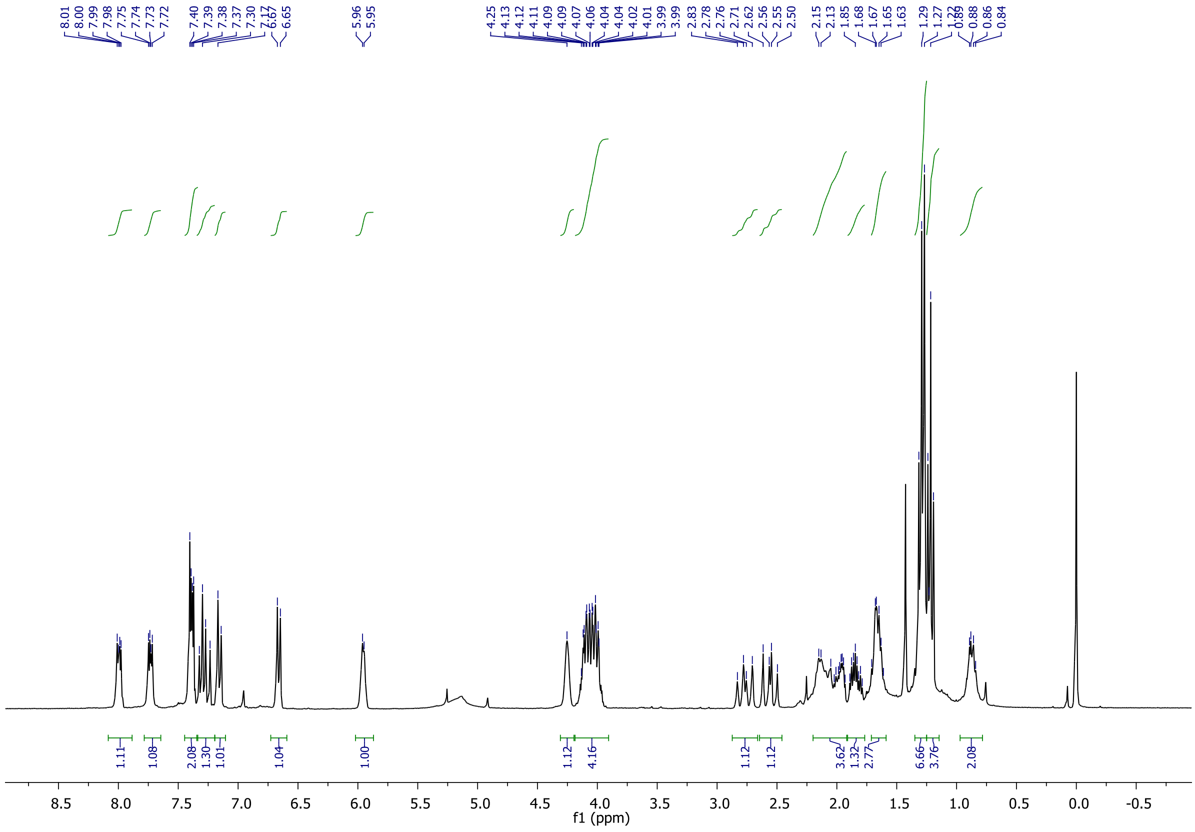

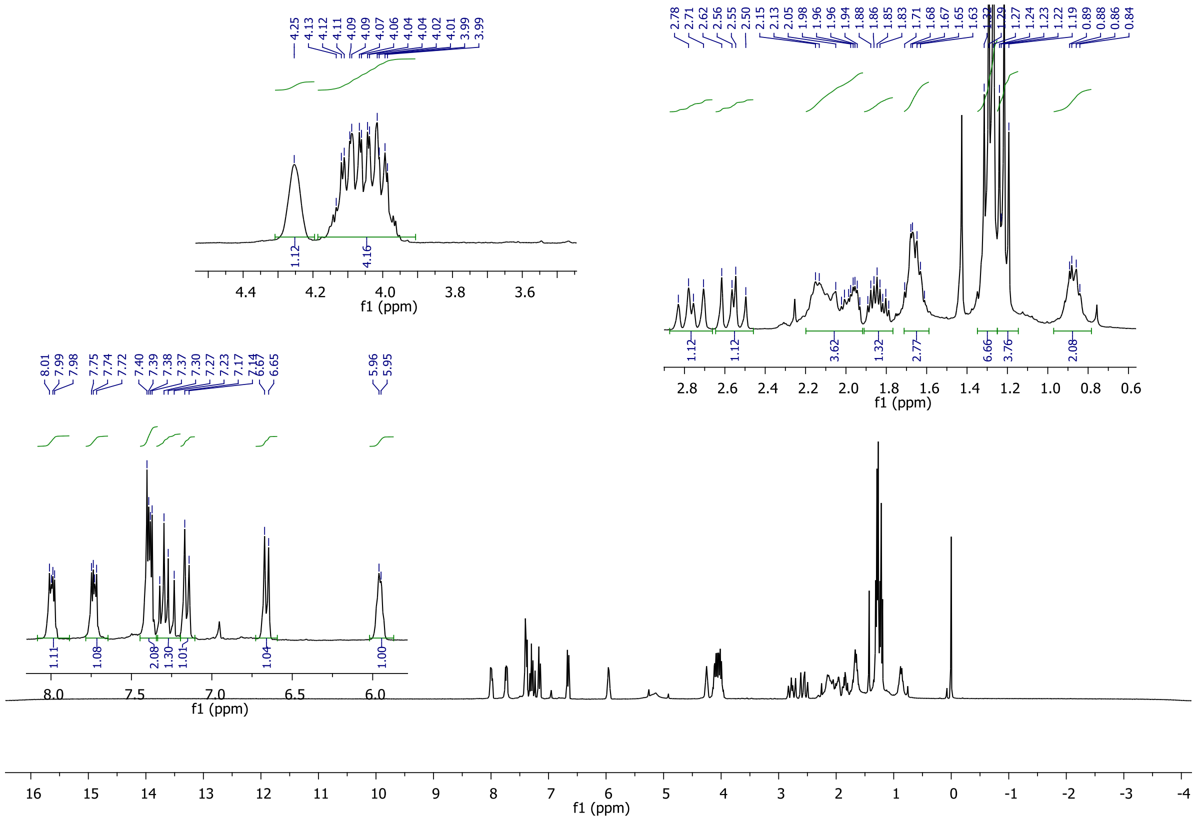

^{13}C NMR (75MHz , $CDCl_3$) :

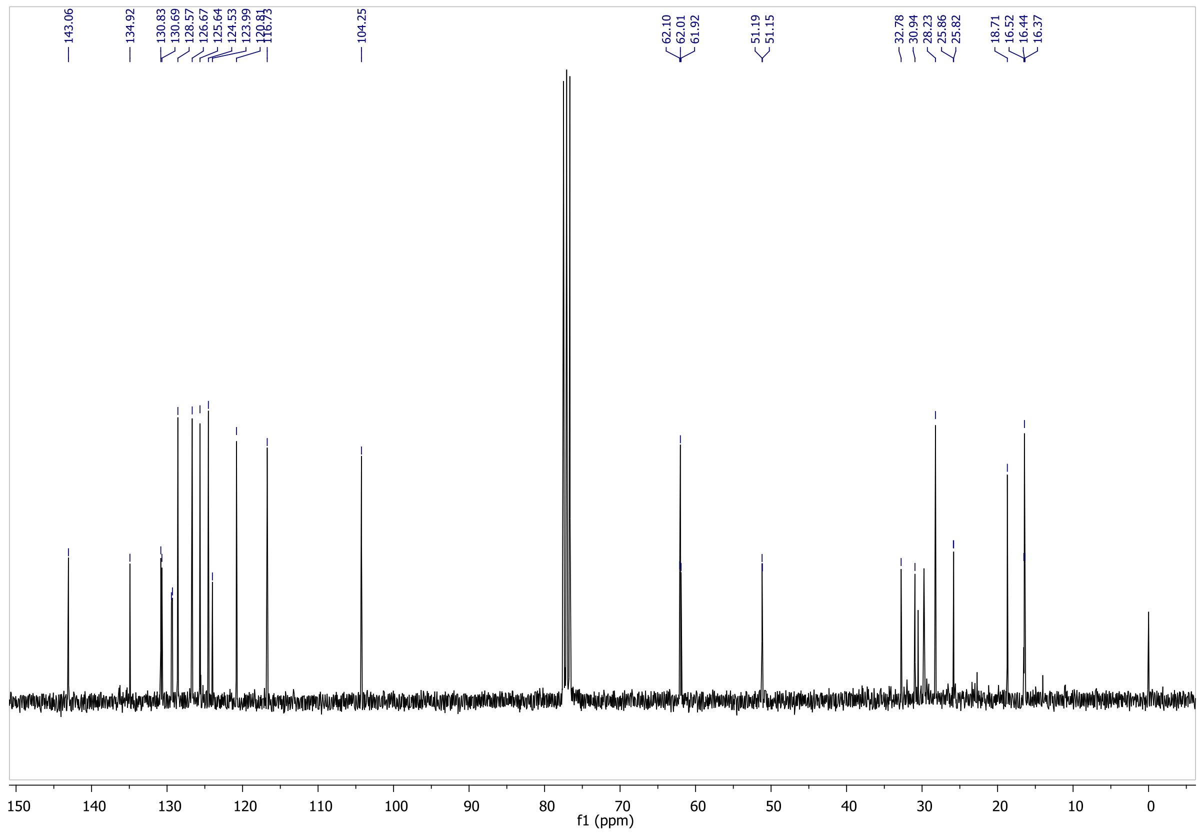

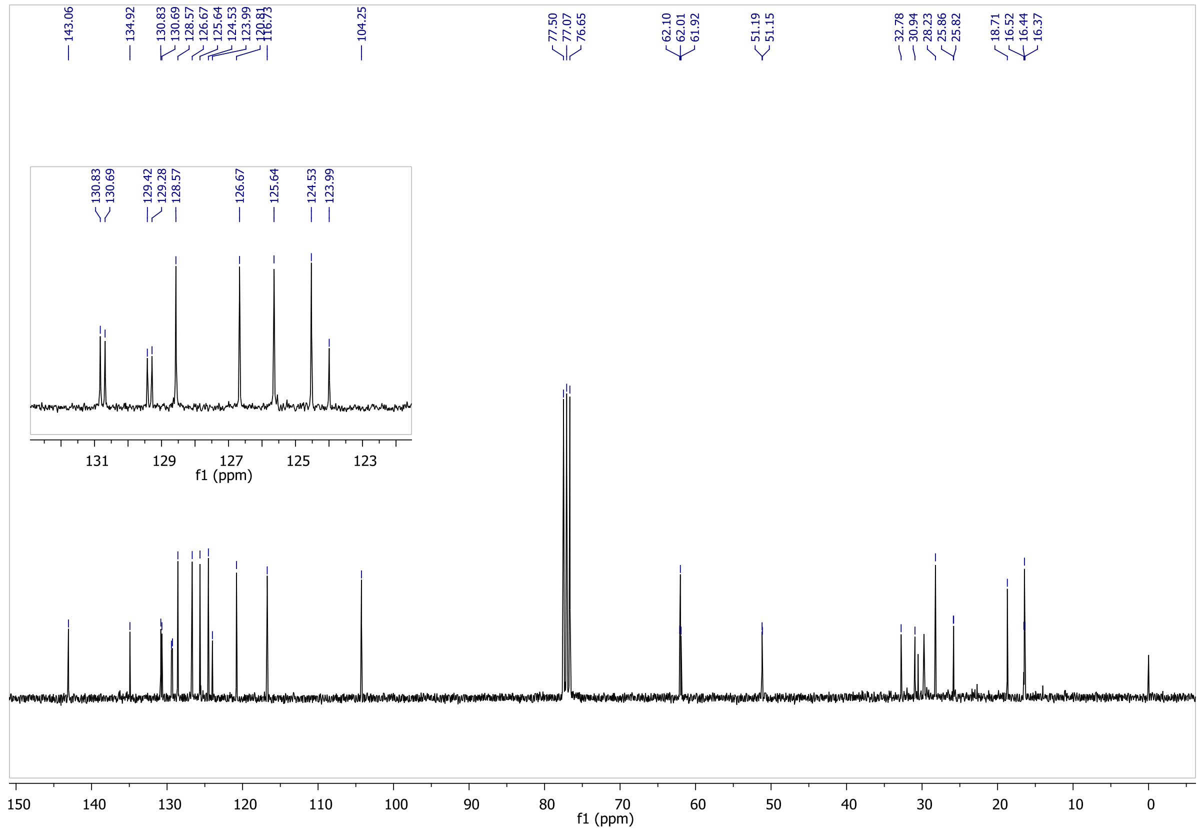

^{31}P NMR (121 MHz, $CDCl_3$):

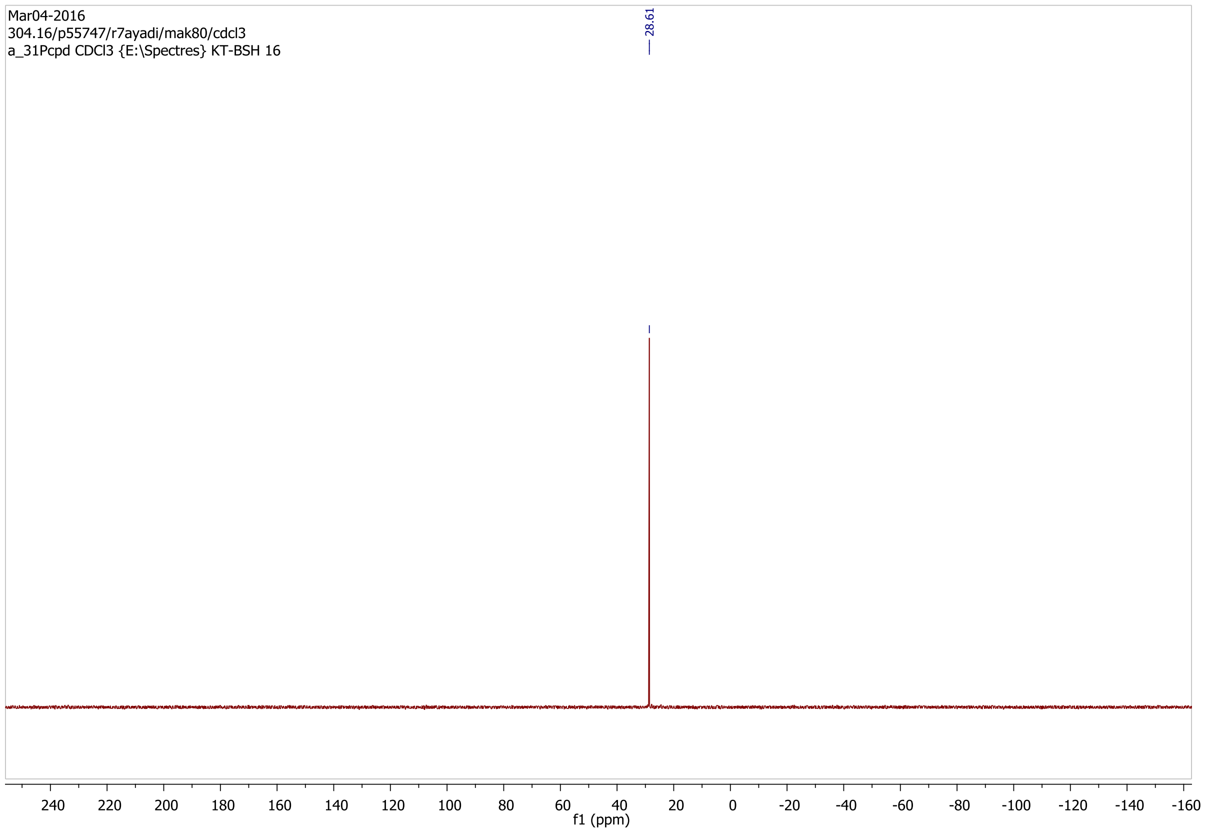

IR (FT-IR):

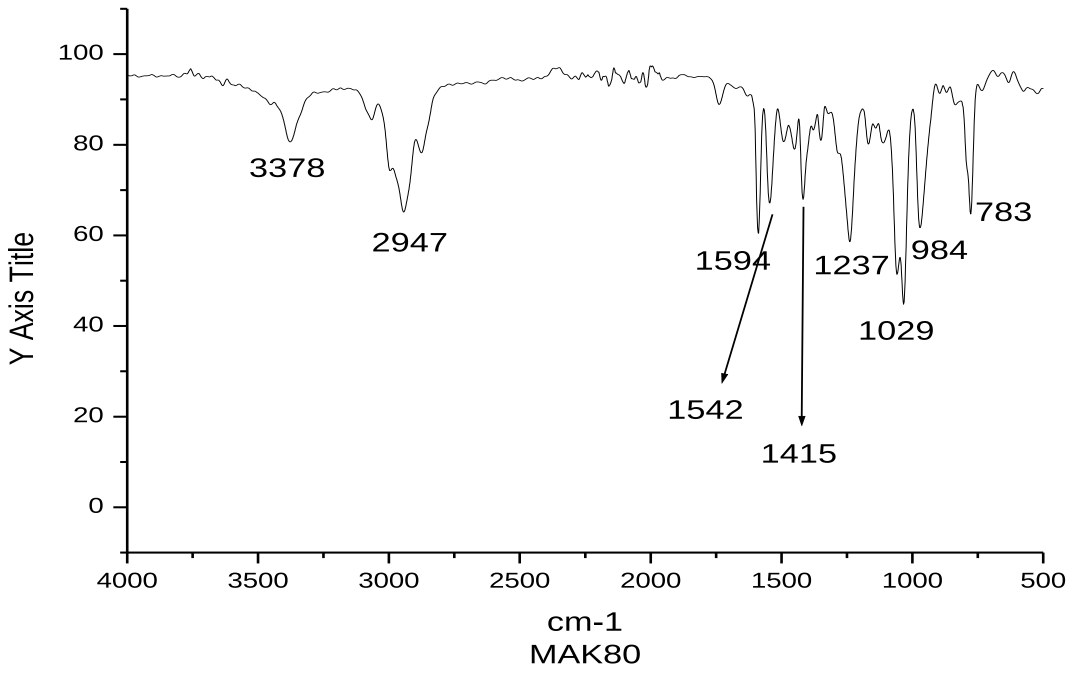

HRMS (ESI+):

Elemental Composition Report **Page 1**

Single Mass Analysis
Tolerance = 1.0 PPM / DBE: min = -10.0, max = 50.0
Element prediction: Off
Number of isotope peaks used for i-FIT = 3

Monoisotopic Mass, Even Electron Ions
2469 formula(e) evaluated with 1 results within limits (up to 50 best isotopic matches for each mass)
Elements Used:
C: 0-100 H: 0-100 N: 0-20 O: 0-20 P: 0-1

SYNAPT G2-S#UEB205 MAK80 17-May-2016
Y-JOM16051701 3 (0.141) Cm (3) 1: TOF MS ES+
3.25e+006

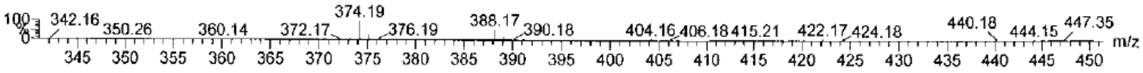

Minimum:				-10.0				
Maximum:		1.0	1.0	50.0				
Mass	Calc. Mass	mDa	PPM	DBE	i-FIT	Norm	Conf(%)	Formula
374.1883	374.1885	-0.2	-0.5	8.5	2108.2	n/a	n/a	C21 H29 N O3 P
